# The blood–brain barrier and the neurovascular unit in subarachnoid hemorrhage: molecular events and potential treatments

**DOI:** 10.1186/s12987-022-00312-4

**Published:** 2022-04-11

**Authors:** Peter Solár, Alemeh Zamani, Klaudia Lakatosová, Marek Joukal

**Affiliations:** 1 Department of Anatomy, Cellular and Molecular Neurobiology Research Group, Faculty of Medicine, Masaryk University, 625 00 Brno, Czech Republic; 2 Department of Neurosurgery, Faculty of Medicine, Masaryk University and St. Anne’s University Hospital Brno, Pekařská 53, 656 91 Brno, Czech Republic

**Keywords:** Subarachnoid hemorrhage, Blood–brain barrier, Subarachnoid hemorrhage treatment, Neuronal injury, Neurovascular unit, Neuroinflammation

## Abstract

The response of the blood–brain barrier (BBB) following a stroke, including subarachnoid hemorrhage (SAH), has been studied extensively. The main components of this reaction are endothelial cells, pericytes, and astrocytes that affect microglia, neurons, and vascular smooth muscle cells. SAH induces alterations in individual BBB cells, leading to brain homeostasis disruption. Recent experiments have uncovered many pathophysiological cascades affecting the BBB following SAH. Targeting some of these pathways is important for restoring brain function following SAH. BBB injury occurs immediately after SAH and has long-lasting consequences, but most changes in the pathophysiological cascades occur in the first few days following SAH. These changes determine the development of early brain injury as well as delayed cerebral ischemia. SAH-induced neuroprotection also plays an important role and weakens the negative impact of SAH. Supporting some of these beneficial cascades while attenuating the major pathophysiological pathways might be decisive in inhibiting the negative impact of bleeding in the subarachnoid space. In this review, we attempt a comprehensive overview of the current knowledge on the molecular and cellular changes in the BBB following SAH and their possible modulation by various drugs and substances.

## Introduction

Subarachnoid hemorrhage (SAH), a life-threatening emergency condition, occurs mainly due to the rupture of a cerebral artery aneurysm. SAH remains a major cause of mortality with a poor prognosis as therapeutics are elusive [1]. Pharmacological treatment is limited to nimodipine, which should be administered to all patients following aneurysmal SAH as recommended in the 2012 guidelines [2]. Nevertheless, continuous intra-arterial nimodipine infusion is associated with side effects such as higher intracranial pressure (ICP), reduction of systolic and diastolic blood pressure, more frequent infectious complications, and reduced motility of the gastrointestinal tract [3, 4]. Therefore, it is necessary to focus on finding other possible pharmacological treatments for SAH, and in order to successfully do that, we need to understand the pathophysiological cascades leading to the consequences of SAH. Currently, experimental studies are increasingly focused on the cellular and molecular mechanisms of pathophysiological cascades following SAH. The cerebrovascular system constituting the blood–brain barrier (BBB) is composed of various interacting cells, including neurons, astrocytes, microglia, pericytes, endothelial cells, and vascular smooth muscle cells (VSMC). Several advances have been made in understanding the responses of individual cells as well as their interactions with other cells following SAH. Many pathophysiological cascades are currently known from experimental studies, and these cascades have been experimentally targeted by various natural and synthetic substances. The beneficial effects of some of these drugs have been tested in clinical trials. However, the complexity of SAH-induced reactions makes it difficult to find an effective drug or drug combination that would positively affect patient outcome following SAH. We, therefore, set out to summarize the current knowledge on the pathophysiological interactions between neurons, astrocytes, microglia, pericytes, endothelial cells, and VSMC induced by SAH. We also present a list of potential drugs for SAH treatment.

We performed a comprehensive review of the literature indexed in PubMed, Medline, ResearchGate, ScienceDirect, Elsevier, Wiley Online Library, EMBASE, Oxford journals, Cambridge journals and SAGE journals databases. The search terms were subarachnoid hemorrhage and endothelial cells or pericytes or astrocytes or microglia or neurons or vascular smooth muscle cells. Articles for this review were selected based on publications published from 2000 to the present in journals with impact factors; it was further based on the number of citations and the significance of their contribution to the understanding of the pathophysiological mechanisms induced by SAH. Articles not related to or not focused primarily on SAH were excluded as were those not published in English. Disputations and disagreements were resolved by means of discussion to arrive at a consensus among all participating authors.

## Anatomy of the blood–brain barrier and the neurovascular unit

### Endothelial cells and junction proteins

Endothelial cells (ECs) are the main component of the BBB. These cells are held together by proteinaceous junctional complexes such as tight junctions, adherent junctions, and gap junction proteins [5, 6].

The molecular complexity of tight junctions (TJs) modulates BBB integrity by creating an electrical resistance (1500–2000 Ω/cm^2^) that depends on extracellular calcium concentration [7].

TJs are situated on the apical membrane of ECs and consist of transmembrane proteins [such as claudin, occludin, and junctional adhesion molecule (JAM)] and cytoplasmic proteins that connect transmembrane proteins with the cytoskeleton [7, 8].

Claudins belong to a group of more than 20 proteins that contain four transmembrane domains and two extracellular loops. They are connected through cis- or trans-interactions with the plasma membrane forming dimers or polymers [9, 10]. The typical claudins that form the TJs of ECs are claudin-1, -3, -5, and -12.

Permeability of molecules of a certain size is controlled by different claudins [8]. For instance, Claudin-5 has a direct effect on BBB permeability to small molecules (< 0.8 kDa). In addition, it has been described that baicalin application upregulates claudin-5 in the ECs, leading to decreased BBB permeability and inhibition of toxic free radicals damage in the brain, consequently reducing brain edema following stroke [11]. Interestingly, this protein is degraded following an ischemic insult [8]. Claudins play different functional roles in barrier formation due to their structural differences. Particularly, claudin-1, -3 and -5 form stronger cell–cell contact, compared with claudin-12 [10].

Occludin was the first TJ protein that was discovered [12], and it plays an important role in the maintenance of BBB rather than in developing the barrier [7]. Its function is to limit small molecules from passing through BBB [10]. Thus, its deficiency can influence paracellular permeability [13, 14].

Another member of the TJ protein complex is the junctional adhesion molecule (JAM)-A, -B, and -C. These single-transmembrane proteins occur extensively in the central nervous system (CNS) endothelial cells, especially JAM-A [15]. JAM-A communicates with scaffolding proteins and is important for TJ function. It acts as a barrier against molecules larger than 4 kDa and can maintain BBB permeability even when claudin proteins are deficient [10, 16–18]. JAMs control integrins and can affect them indirectly by changing their expression. During inflammatory processes, they can influence leukocyte trafficking and impact the immune system [19–21].

TJ transmembrane proteins are connected with the cell cytoskeleton by cytoplasmic proteins—the peripheral membrane-associated guanylate kinase (MAGUK) family of proteins, namely, zonula occludens (ZO)-1, -2, -3 and, cingulins [22, 23]. They have a special effect on the correction of the spatial supply of claudins [21]. It was provided experimentally that decreased production of ZO-1 and occludin increased BBB permeability [22].

The barrier function of the TJs is not associated only with the expression of claudins and occludin bridging the intercellular gaps, it is also affected by the protein organization and their interactions in the barrier, as well as a number of other cell types present in the region (e.g., pericytes and astrocytes) [24]. The manifestation of occludin and adherent junctions has also an effect on TJs function [25].

Adherent junctions located below the TJs and closer to the basolateral membrane, have a similar organization as TJ proteins. Adherent junction proteins. They comprise cadherins (transmembrane glycoproteins) and cytoplasmic proteins such as catenin (α, β, and γ). The interactions between cadherins are Ca^2+^- dependent. Vascular endothelial cadherin (VE-cadherin) plays a crucial role in vascular organization. It is important not only for EC adhesion but also for decreasing cell permeability [7, 26, 27].

Adherent junctions strengthen the connections between the endothelial cells and regulate paracellular permeability [7]. They play a crucial role in the mechanical support for cells and are fundamental for TJ functionality [28].

Gap junctions (GJs) are formed by transmembrane isomers—connexins (CX). GJs between brain ECs express CX37, CX40, and CX43. These junctions form channels between ECs and help maintain TJ integrity [27]. GJs have an important role in intracellular communication. For example, ions and small molecules can pass through these junctions [8].

### Basement membrane, astrocytes, and pericytes

ECs are surrounded by a layer comprising pericytes and astrocyte endfeet and are separated from them by a basement membrane [29, 30]. These cells, along with the basement membrane, together reinforce BBB structure [21].

As a sheet-like component of the extracellular matrix, the basement membrane acts as structural support for ECs. The basement membrane contains protein complexes made of collagen IV, laminins, nidogen, and perlecan. Collagen IV interacts with ECs, growth factors, and other basement membrane components. Laminins are a large group of extracellular matrix glycoproteins with a trimeric structure that consists of three α, β, and γ chains and are essential for the organization of the basement membrane [31].

The structural composition of the basement membrane—mainly due to adhesion receptors, which have supporting functions—plays a vital role in the manifestation of BBB properties [25, 32]. These adhesion receptors are integrins α1β1, α3β1, α6β1, and αvβ1/αvβ3, and dystroglycan [25]. Integrins are a group of heterodimeric transmembrane receptors regulating cell activity and the connection between matrix and cytoskeleton. Dystroglycan is a single heterodimeric transmembrane receptor connecting the cytoskeleton with the matrix [32].

Both pericytes and brain ECs are anchored to the same basement membrane. Pericytes surround ECs with their cytoplasmic projections—surrounding from 30 to 70% of the endothelial walls depending on the type of microvessel [33]. The most common distance between ECs and pericytes is 20 nm [34], and different types of connections are distinguishable between these cell types. The intracellular connection is secured by gap junctions, TJs, and adherent junction proteins [27, 33]. The main function of pericytes is to maintain vessel stability through growth factors and angiogenic molecules [35, 36], but they also affect brain microcirculation, thanks to their synapse-like peg-socket contact [21]. In vitro experiments suggest that pericytes reinforce BBB permeability, support vascular integrity, and participate in the development of the BBB [37].

Astrocytes are a group of glial cells that surround brain ECs with their endfeet and are responsible for homeostasis in the brain microenvironment [38]. They are also responsible for regulating immune reactions and supporting BBB integrity [21, 39, 40]. In vitro experiments suggest that the establishment of TJs during brain development is more efficient if astrocytes are present [41].

### Neurovascular unit—the communicative networking of the BBB

Pericytes located between ECs and basement membrane, neurons, astrocytic endfeet, and microglia—all together form a neurovascular unit (NVU) [21, 42, 43]. All NVU components contribute to maintaining a stable and functional BBB, while receptors, transporters, and ectoenzymes regulate transmission through the BBB at the molecular level. NVU components interact and enable the establishment in the CNS of different ionic microenvironments, thus ensuring stable neuronal function. These functions include specialized roles in the neurotransmitter pool, maintaining a low protein concentration to reduce cell proliferation, protecting CNS from exposure to toxins and consequent neuronal damage, and avoiding inflammatory processes by regulating the passage of inflammatory cells through the barrier [43].

BBB endothelial cells sitting on the walls of blood vessels possess a series of highly specialized properties that strictly limit the passage of molecules, ions, and immune cells between the blood and brain parenchyma. Nevertheless, the crosstalk among endothelial, vascular, glial, neural, and immune cells is essential for the integrity and the dynamic properties of BBB. Recently, Banks et al. used an in vitro model to examine the interactions of NVU elements in relation to BBB integrity and cytokine secretion. They showed that only four cytokines [granulocyte colony-stimulating factor (G-CSF), keratinocytes-derived chemokine, monocyte chemoattractant protein-1 (MCP-1), and RANTES] were released from EC monocultures in response to stimuli, while tri-cultures of pericyte/astrocyte/ECs accumulate a higher level of these cytokines along with five other cytokines—interleukin (IL)-6, IL-13, MIP-1 α, MIP-1 ß, and TNF—that could significantly alter BBB integrity [44]. It is worth mentioning that EC properties are modulated not only by signaling molecules from pericytes, astrocytes, and neurons, but EC-induced signaling molecules are also necessary for the proper activity of neurons, astrocytes, and pericytes [45]. For instance, brain-derived neurotrophic factor (BDNF), a neuroprotective agent, is secreted in large amounts by cerebral ECs. Interestingly, ECs, astrocytes, and neurons all express the receptors tropomyosin receptor kinase B (TrkB)-FL, TrkB-T1, and pan75 neurotrophin receptor (p75NTR)—all of whom are recognized by BDNF [46]. Gue et al. showed that cerebral ECs could protect neurons via upstream TrkB and protein kinase B (Akt) signaling and downstream caspase suppression [47]. Furthermore, it was reported that disabled-1 expressed by brain ECs regulates the communication of vessels with the astrocytes and plays a key role in both neuronal migration and NVU function [48]. Moreover, there are indications that the differentiation of astrocytes is supported by EC-induced leukaemia inhibitory factor-1 [49].

Similar to other blood vessels, the luminal BBB surface is covered by a glycocalyx layer that acts as a primary barrier. At the abluminal surface of the ECs, pericytes are embedded in the basement membrane and closely interact with ECs [50]. Pericytes have the actin-myosin system (including alpha-smooth muscle actin (α-SMA), tropomyosin, and myosin proteins) that is associated with cell contraction are involved in controlling capillary diameter [51–53]. In vitro studies have revealed that constriction/dilatation of pericytes is regulated by receptors and the signaling machinery of pericytes that can respond to endothelium-derived vasoactive mediators [such as endothelin-1 (ET-1) and nitric oxide (NO)] and neurotransmitters (including serotonin, histamine, and noradrenaline) [54].

Reports have demonstrated that pericytes of the BBB play key roles to limit transcytosis as well as expression of leukocyte adhesion molecules (LAMs), resulting in lowered leukocyte infiltration. Particularly, pericyte deficiency has been shown to alter the expression of occludin, claudin-5, and ZO-1 and increase the bulk-flow transcytosis of BBB [55]. Moreover, it was shown that inhibition of pericyte-derived transforming growth factor-β1 (TGF-β1) induced by cyclosporin A could alter BBB integrity through P-glycoprotein (P-gP) dysfunction [56]. Further, it was reported that astrocyte-EC interaction could also be affected by cyclosporin A, resulting in a misregulated BBB [57, 58].

Astrocytic endfeet connect ECs and pericytes to surrounding neurons. Evidently, changes in neural activity can influence pericyte or EC function. Also, water homeostasis at the NVU is regulated by astrocytes via aquaporin (AQP)-4, and Kir4.1 expressed in astrocytic endfeet [49]. Astrocytes can also regulate the expression of TJ proteins and EC transporters, as well as promoting the EC response to inflammatory stimuli.

Moreover, loss of contact between ECs and astrocytic endfeet can result in enlarged vessels [59]. In line with this, it has been reported that the gap junctions between astrocytes can upregulate cytokine expression and hence increase leukocyte trafficking across BBB [60, 61]. The role of astrocytes in BBB maintenance has been defined as necessary and nonredundant. Using a mouse model, astrocyte ablation has been shown to damage BBB to varying extents [62].

A recent review focused on the role of G protein-coupled receptors (GPCRs) in BBB development and function discussed intercellular signaling mediated by GPCRs in the NVU [63]. Intercellular interactions between neurons and ECs are modulated via Wnt/Frizzled signaling, a member of the GPCR family, astrocytes communicate with ECs via the Shh/SMO signaling pathway, and finally, pericyte-EC interaction is regulated by sphingosine 1-phosphate (S1P)/S1PR signaling.

Microglia are immune cells that originate from leptomeningeal mesenchymal cells and are activated during inflammatory reactions in BBB. Ramified microglia are transformed into ameboids and finally to phagocytic microglia [64]. During these processes, TJs can be disrupted due to the influence of cytokines [65]. In summary, we can conclude that NVU components and their function are closely linked and are therefore essential for BBB physiology.

### The Virchow-Robin space

The Virchow-Robin space (VRS) originally identified by Virchow and Robin is the space that surrounds blood vessels (arterioles and venules) penetrating from the subarachnoid space into the brain [66–68]. The artery entering the brain loses the outermost tunica adventitia and is encased in a layer of pia mater and the adjacent glia limitans formed by astrocytic endfeet processes. However, there is no empty VRS between the artery and glia limitans, instead, compact layers of cell processes and pial-glial basement membrane are formed partly by the pia mater and partly by glia limitans (membrana limitans gliae perivascularis). The brain VRS gradually narrows as we move from the surface of the brain deeper into the brain parenchyma. As the artery enters deeper into brain tissue and divides into capillaries, the pia mater, as well as the tunica media, are lost. At the level of capillaries, the glia limitans is in contact with the capillary wall. The capillary wall is formed from two components—the endothelium and the basement membrane. On the capillary is the basement membrane, derived from endothelial cells, and on the other side from astrocytes of the glia limitans. The capillary basement membrane encapsulates the pericytes that lie between the basement membrane of the glia limitans and the endothelium [69, 70]. Cerebrospinal fluid (CSF) with solutes passes through the pia mater and flows along the penetrating arteries towards the capillary basement membrane, and mixes with the interstitial fluid. Fluid with waste solutes then passes through similar channels along venous capillaries and reaches the subarachnoid space. This paravascular or “glymphatic” pathway is dependent on trans-astrocytic water movement mediated by AQP-4 [71–74]. Periarterial, intramural or lymphatic drainage channels drain interstitial fluid and solutes from brain parenchyma through the basement membrane between adjacent smooth muscle cells in the tunica media of the artery and reach cervical lymph nodes. The motive force for solute drainage from brain parenchyma in the direction opposite to that of blood flow probably depends on vascular pulsation [75–77].

### Transporter system of the BBB

Although traffic across the BBB is regulated by a complex system of transporters and receptors present on BBB ECs [apart from the control exerted by physical properties of the barrier (e.g., by junction protein complexes)], small lipophilic molecules and a few gases such as O_2_ and CO_2_ can freely cross the BBB into and out of the brain parenchyma. In particular, molecular trafficking between blood and the brain is tightly controlled by efflux transporters, nutrient transporters, and ion channels that maintain a stable chemical environment in the CNS. The expression of transporters is not identical in the luminal and abluminal surfaces of the BBB endothelial cells, resulting in the polarized features of this barrier, which is crucial for its function. Understanding the transport system of the BBB is not only essential in terms of misregulated BBB but also enables the development of new drug delivery strategies where BBB acts as a formidable obstacle in therapy [78].

Active efflux transporters expressed mainly at the luminal side of ECs utilize ATP to move drugs, xenobiotics, drug conjugates, and nucleosides up their concentration gradients from ECs into the blood [79]. The most abundant ATP-binding cassette (ABC) transporters of the BBB are MDR1, also known as P-glycoprotein (P-gP), and breast cancer resistance protein (BCRP). Impaired P-gP-mediated efflux can cause neuronal cell death [80].

Nutrient transporters facilitate the entry of carbohydrates, amino acids, hormones, fatty acids, nucleotides, organic anions, cations, and vitamins into the brain. Specific types of nutrient transporters can also remove excess molecules and deliver them into circulation.

Glucose, the key energy source for the brain, is transported via glucose transporter (GLUT)-1/3 and SGLT-1, members of solute carrier-mediated transporter (CMT). The expression of glucose transporter 1 (GLUT-1) is regulated by Wnt-signalling, and although it is enriched on the abluminal side of the endothelial membrane [52, 83], glucose is transported in both directions. Na^+^/myo-inositol transporter (SMIT) and H^+^/myo-inositol symporter (HMIT) provide the brain with myo-inositol—one of the most abundant metabolites of the brain [27, 55].

Organic anion transporting polypeptide transporters (OATP) can transport organic anions and thyroxine in both directions [5]. OATP-2 has been shown to transport valproic acid, the most common antiepileptic drug [84]. One study has confirmed that the functional expression of OATP-1a4 is sex-specific in rats, being upregulated in female rats compared to males [85].

CMTs can also transport amino acids (AA) across the BBB. Glutamine and small neutral AAs are removed from the brain via the sodium-coupled neutral AA transporter (SNAT)-1-3, while SNAT-5 transports glutamine bidirectionally. To limit the toxic effects of excitatory AAs on neurons, sodium-dependent excitatory AA transporters (EAAT)-1-3 transport glutamate and aspartate out of the brain. Sodium-dependent transporters of AAs have been shown to be expressed only on the abluminal membrane of the ECs [27, 83].

The primary substrates for DNA and RNA synthesis (nucleotides and nucleobases) are supplied to the brain by sodium-independent equilibrated nucleoside transporter (ENT)-1 and -2 and are returned to the blood via sodium-independent concentrative nucleoside transporter (CNT)-2. Choline is transported bidirectionally via choline transporter-like protein 1 (CTL-1) [81, 86].

In addition to CMTs that facilitate the transport of regulatory proteins and hormones, the trafficking of some proteins is mediated at a slower rate than CMT transport by receptor-mediated transporters (RMT). Transferrin, insulin, and leptin cross the BBB into the brain by transferrin receptor (TfR), insulin receptor (IR), and leptin receptor (LEP-R), respectively. Bidirectional transport of arginine-vasopressin is mediated via the V1 vasopressinergic receptor. Lipoprotein receptor-related protein (LRP)-1 is expressed on the abluminal surface of the ECs and mediates the clearance of amyloid-ß and apolipoprotein E (ApoE)-2 and -3 from the brain. LRP2 also participates in the efflux of amyloid-ß 42 into the blood. Receptor for advanced glycation end products (RAGE) expressed on the luminal side of the ECs, transports amyloid-ß into the brain [52, 81, 86].

Moreover, the major facilitator superfamily domain-containing protein (Mfsd2a) expressed exclusively in brain ECs, transports docosahexaenoic acid (DHA)—an essential omega-3 fatty acid into the brain. It has been shown that Mfsd2a plays a crucial role in BBB functional integrity [86, 87].

Finally, the ion balance required for proper CNS function is mainly maintained by ion transporters in the BBB [21, 27, 52]. Intracellular endothelial pH is regulated by the Na^+^H^+^-exchanger (NHE), which imports sodium and transports protons into the blood. Sodium is also pumped into the brain via the sodium pump (Na^+^K^+^-ATPase) expressed on the abluminal side of the ECs, ensuring the proper function of sodium-dependent transport [83], Na^+^K^+^-ATPase also regulates the efflux of potassium from the brain. On the luminal side, the Na^+^K^+^Cl^−^-cotransporter (NKCC1) transports Na^+^, K^+^, and Cl^−^ into the brain. Efflux of Na^+^ and HCO_3_^−^ from the ECs into the brain is mediated by Na^+^HCO_3_^−^-exchangers in a Cl^−^-dependent (via NDCBE) or Cl^−^-independent (via NBCe1 and NBCn1) manner [88]. The low intracellular calcium level in microvascular endothelium is maintained by Na^+^Ca^2+^-exchanger (NCX) that also pump out Ca^2+^ from the brain and can reverse function under pathological conditions [89]. Calcium influx into ECs is regulated by the transient receptor potential (TRP) channels expressed on ECs abluminal membrane [90]. The voltage-gated K^+^ channel Kv1 and the inward-rectifying K^+^ channel (Kir)-2 transport potassium outwards, resulting in EC hyperpolarization and blood flow regulation due to vasodilation [91].

Apart from the highly specialized limited transport of molecules modulated by the polarized nature of ECs, slow transcellular movement of molecules can also occur through transcytosis. However, pathological conditions can increase the number of vesicles, leading to BBB hyper-permeability [92]. It was recently shown that increased transcytosis and BBB-permeability could be exclusively dependent on caveolin-1 in cortical spreading depolarizations [93].

The vulnerability of BBB during pathology has also been explained by the activation of matrix metalloproteinase (MMP), a zinc-dependent protease expressed in ECs. Activation of MMPs can promote the degradation of BBB extracellular matrix and TJ proteins, resulting in the BBB-rupture. It has been reported that the consequent production of NO in response to cerebral ischemia can downregulate caveolin-1 and thus activate MMP [94]. In line with this, therapeutics such as glucocorticoids that target the tissue inhibitor of metalloproteinases TIMP-3 has been shown to enhance BBB integrity and promote the expression of claudin-5 and occludin [95–98]. Moreover, it is known that pathology can promote the entry of leukocytes into the CNS by increasing the expression of leukocyte adhesion molecules in ECs [52].

### BBB and Neurotransmitters

Administration of catecholamines, such as dopamine, norepinephrine, and epinephrine, can alter the expression level of TJ and adherent junction proteins, thus increasing BBB permeability [99, 100]. An in vitro model of ischemia has shown that activation of the β2-adrenergic receptor, a receptor for norepinephrine, can induce occludin down-regulation and BBB damage [101]. It was demonstrated that hypoxia-inducible factor-1 alpha (HIF-1α) was upregulated in ischemic neurons, resulting in neuronal MMP-2 secretion and vascular endothelial growth factor-A (VEGF-A) up-regulation. This result suggests that degradation of occludin in the ECs is mediated by the interaction between neurons and ECs rather than the direct effects of HIF-1α on ECs.

Besides, bEnd.3 cells, an in vitro BBB model, exhibit a high level of acetylcholine receptor (AchR) expression [102]. It was shown that in this cell line, the cellular uptake of a dopamine derivative molecule (BPD) is mediated by AchR. Abbruscato et al. have shown that in another in vitro BBB model (BBMEC), nicotinic AchR mediates the down-regulation of ZO-1 and BBB hyperpermeability in response to stroke. These cells were exposed to nicotine prior to the stroke [103].

## Subarachnoid hemorrhage

Neuronal cells, as well as glial, endothelial, and vascular smooth muscle cells, are the main components of the recently proposed concepts such as that of the NVU. An extension of the NVU is the so-called vasculo-neuronal-glia triad model that includes neurons, astrocytes, capillary endothelial cells, pericytes, smooth muscle cells, noncapillary endothelial cells, perivascular nerves, smooth muscle progenitor cells, and veins—in short, all the components required to maintain brain function [104–106].

The prevention and treatment of non-traumatic subarachnoid hemorrhage (SAH) has remained a challenge for decades. The worldwide incidence of SAH shows a declining trend with large regional differences [107]. Despite up-to-date treatment of SAH, the median case fatality remains high—varying between 27 and 44% for individual regions [108]. The leading cause of SAH is the rupture of an intracranial aneurysm which accounts for about 80% of cases. The extravasation of blood following SAH into subarachnoid spaces filled with CSF initiates a complex cascade leading to CNS damage [109, 110]. The two main consequences after SAH are an early phase called early brain injury (EBI), and a later phase termed delayed cerebral ischemia (DCI). EBI is defined as a pathophysiological cascade in the first 72 h after SAH, including rapid changes in intracranial pressure, cerebral perfusion pressure, cerebral blood flow, ionic changes, cortical spreading depolarization, impaired calcium homeostasis in cerebral vessels, increased extracellular glutamate, mechanical stress, etc. [111, 112]. On the other hand, DCI develops 3–14 days after the initial bleeding. Most authors define DCI as symptomatic vasospasm, cerebral infarction attributable to vasospasm, or both [113–115].

**Fig. 1:**
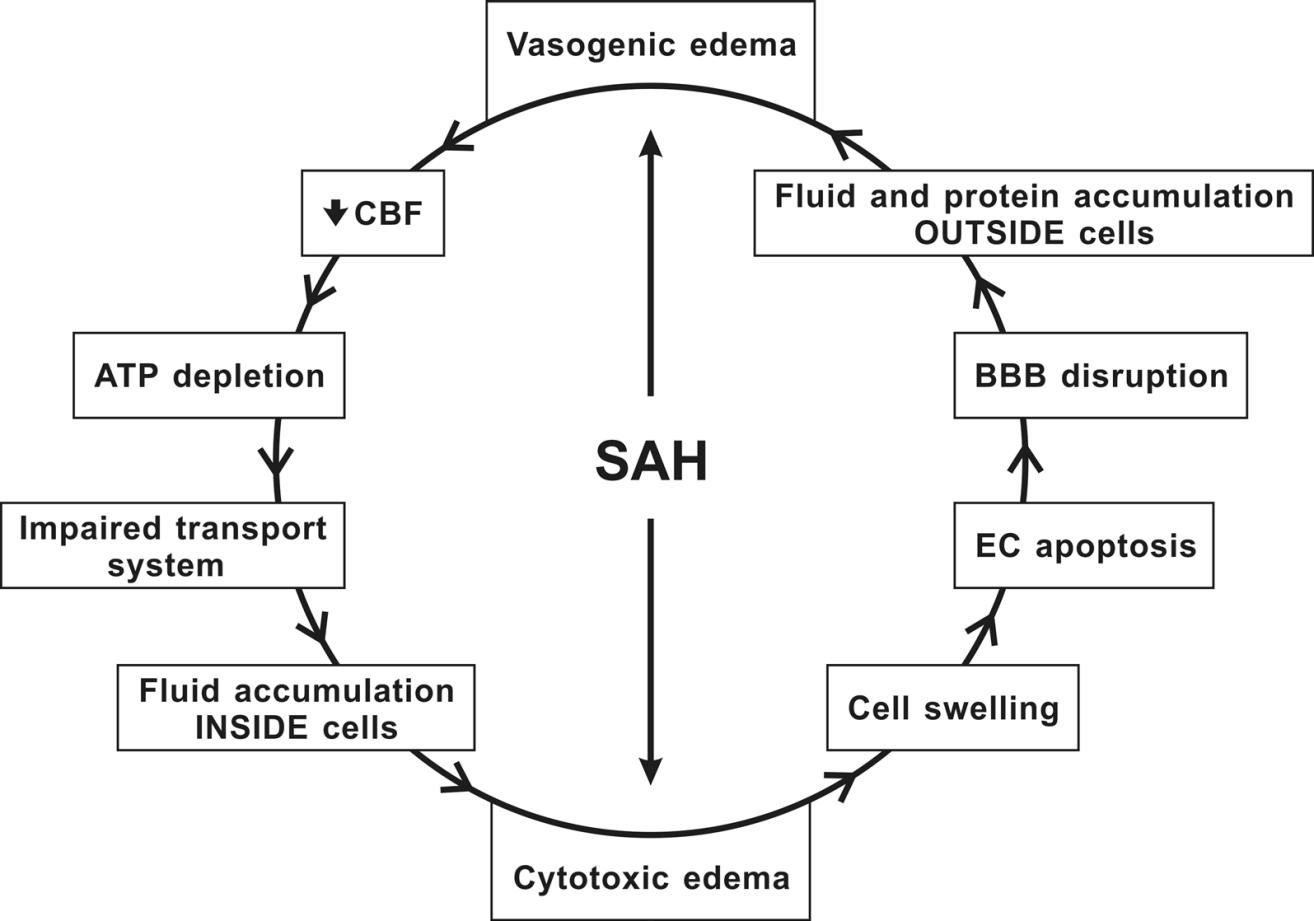
Pathophysiology of brain edema during subarachnoid hemorrhage. Intracranial pressure (ICP), one of the immediate responses to subarachnoid hemorrhage (SAH), can cause both vasogenic and cytotoxic edema. Cytotoxic edema, characterized by cell swelling and apoptosis of endothelial cells (ECs), results in disruption of BBB, which ends up with an abnormal accumulation of fluid in brain cells and, eventually, vasogenic edema. Vasogenic edema leads to increased cerebral blood flow (CBF), ATP depletion, and disturbances in cell membrane transport systems leading to abnormal accumulation of fluid in brain cells, which can cause cytotoxic edema

**Fig. 2:**
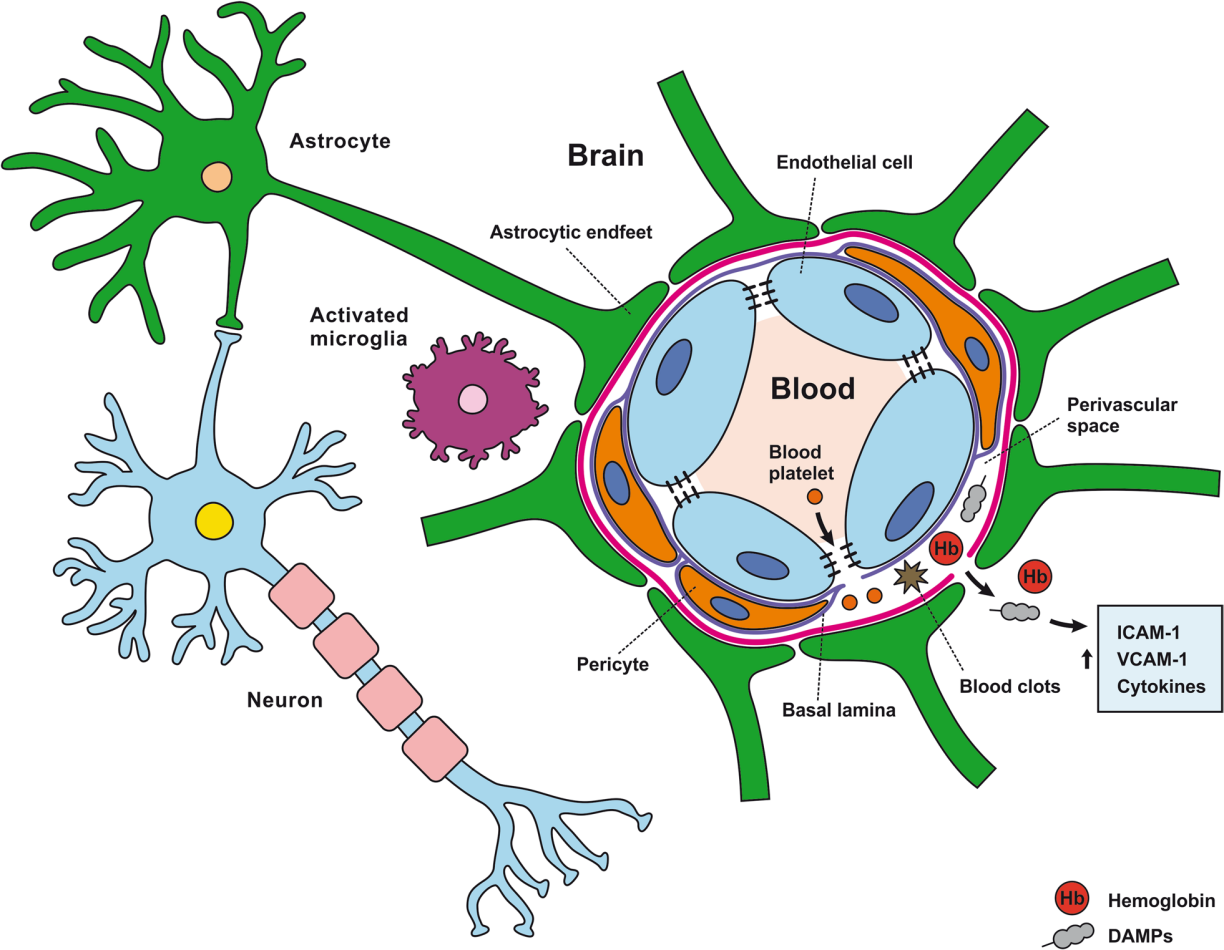
Reaction of the components of the neurovascular unit to subarachnoid hemorrhage. All components of the NVU play vital roles in BBB plasticity and integrity. Research and clinical evidence show that NVU impairment contributes to the development of brain edema in SAH. This includes BBB breakdown, allowing blood to enter into the CNS. As shown, cell swelling, tight junctions, and basal lamina degradation allow the passage of blood components into the brain. When hemoglobin (Hb), damage-associated molecular patterns (DAMP), blood platelets, and clots cross through the barrier, they elicit increased levels of intercellular adhesion molecule-1 (ICAM-1), vascular cell adhesion protein (VCAM)-1, and inflammatory cytokines

It seems that both EBI and DCI are connected and have common mechanisms (Fig. 1) [116, 117]. Moreover, some studies have suggested that EBI and DCI are not different entities, but ischemic brain injury is probably a late manifestation of EBI after SAH [109, 118–120]. Brain edema is one of the major components of EBI following SAH [121–124]. In literature, brain edema is mainly classified into vasogenic and cytotoxic. Vasogenic edema is caused by the extravasation of plasma proteins and the accumulation of fluid in the brain interstitium [125]. It is associated with the degradation of TJ proteins, transcellular channels, and endothelial retractions, as well as with the accumulation of intravascular proteins outside the cells, which result in increased brain volume and ICP. In contrast, cytotoxic edema is characterized by cell swelling caused by ATP depletion and loss of energy for “pumps” like the Na^+^ K^+^-ATPase and Ca^2+^- ATPase. Consequently, secondary transporters such as ion channels and cotransporters are disrupted, including the Na^+^K^+^Cl^−^-cotransporter (NKCC1) and the Na^+^/ Ca^2+^ exchanger. Alteration of cell membrane transport systems leads to abnormal accumulation of fluid in the brain cells [125, 126]. In humans, significant BBB alteration was found as early as 24–48 h following SAH (Fig. 2). Early identification of BBB disruption seen on MRI was associated with disease progression and worse outcomes in patients after SAH [127]. In general, increased BBB permeability is considered to be a negative prognostic factor leading to the development of ischemic complications following SAH [128, 129].

The most immediate event following the rupture of an intracranial aneurysm is sudden increase of the ICP and intracranial circulation arrest. The ICP subsequently decreases over several minutes but remains higher than normal [130]. Sudden decrease in cerebral blood flow (CBF) due to increased ICP is the first step in the pathological cascade leading to development of cytotoxic edema formation, apoptosis of endothelial cells, and BBB disruption, resulting in vasogenic edema and further reduction of CBF [121]. This phenomenon is confirmed by cellular swelling on apparent diffusion coefficient (ADC) maps calculated using MRI with diffusion-weighted imaging (DWI). A sharp decline of ADC observed within 2 min following SAH probably reflects ischemia due to the overall reduction of cerebral blood flow and localized vasospasm. Moreover, decreased ADC values was also observed to a lesser extent in the contralateral hemisphere and with a delay of 1 min in nonheparinized and 3 min in heparinized animals compared to the ipsilateral side [131]. These findings demonstrate development of global cerebral edema in the first minutes following SAH.

Immediately after SAH, several other changes such as increase in ICP, reduction of nitric oxide (NO), release of vasoactive molecules from platelet aggregation, and perivascular glial swelling contribute to disruption of BBB [132, 133]. ICP increase in the first minutes after bleeding into the subarachnoid space leads to a decrease in cerebral blood flow resulting in the reduction of cerebral perfusion pressure (CPP). This initial ischemic insult is probably responsible for the swelling of neurons, astrocytes, and endothelial cells (cytotoxic edema) and creates conditions amenable for aggregation of blood components leading to a non-reflow phenomenon that contributes to acute ischemia after SAH [134]. It was proposed that this non-reflow phenomenon plays a role in the pathophysiology of post-ischemic injury following SAH. Several mechanisms have been found to contribute to the development of the no-reflow phenomenon, including platelet activation, fibrin formation, leukocyte adhesion, or persistent pericyte contraction [135, 136].

Despite the finding of acute ischemic injury, increased permeability of BBB to platelets passing across or around the endothelium and platelet-sized holes (approximately 2–3 µm in diameter) in the basal lamina were found as early as 10 min after SAH [137, 138]. However, there is evidence that following bleeding, blood components spread not only through direct trans-endothelial transfer but also in a paravascular fashion.

Although blood elements in the subarachnoid space are in direct contact with larger vessels, it seems that some blood components such as erythrocytes and damage-associated molecular patterns (DAMPs) like hemoglobin (Hb) may reach BBB through the Virchow-Robin space (VRS) and paravascular spaces surrounding arterioles, capillaries, and venules [139]. The CSF in VRS is pumped into the paravascular space toward the capillary basement membrane completely covered by astrocyte end-feet equipped with AQP-4. CSF/interstitial fluid (ISF) exchange occurs at the level of BBB, and CSF-ISF flows through the paravenous spaces toward the CSF or venous blood [140]. Blood components, as well as serum proteins, quickly diffuse and invade the paravascular space, leading to perivascular glial activation, neuroinflammation, dysfunction in microcirculation resulting in microinfarctions throughout the brain [141].

CSF circulation in the paravascular spaces is impaired following SAH. It was found that aggregation of blood cells and formation of blood clots within the paravascular space block CSF flow as early as 2 min after SAH [141]. This impairment is associated with a decreased ability to clear interstitial solutes from brain [142]. Alteration and occlusion of cerebral paravascular space by coagulated blood may exacerbate edema after SAH [140].

However, blood clots and red blood cells in the subarachnoid space undergo lysis and cell-free Hb distributed in VRS crosses the glial limiting membrane, entering deep into the brain [143]. Larger molecules are trapped in the paravascular space and cannot pass into the cortex because the gap between the astrocytic end-feet constitutes a physical barrier (gap width ~ 20–30 nm). Small molecules from 0.8 to 70 kDa can penetrate the glial limiting membrane to various degrees, while larger molecules from 150 to 2000 kDa are retained in the paravascular spaces [144]. Free Hb (molecular weight of 62.6 kDa) and other DAMPs enter the paravascular spaces and induce recruitment of monocytes [139, 145]. High concentrations of Hb and other vasoactive substances, as well as DAMPs in the paravascular spaces, remain in contact with pericytes [146].

## Reaction to SAH of neurovascular unit cells

### Reaction of endothelial cells to SAH

#### SAH induces apoptosis in endothelial cells

**Fig. 3:**
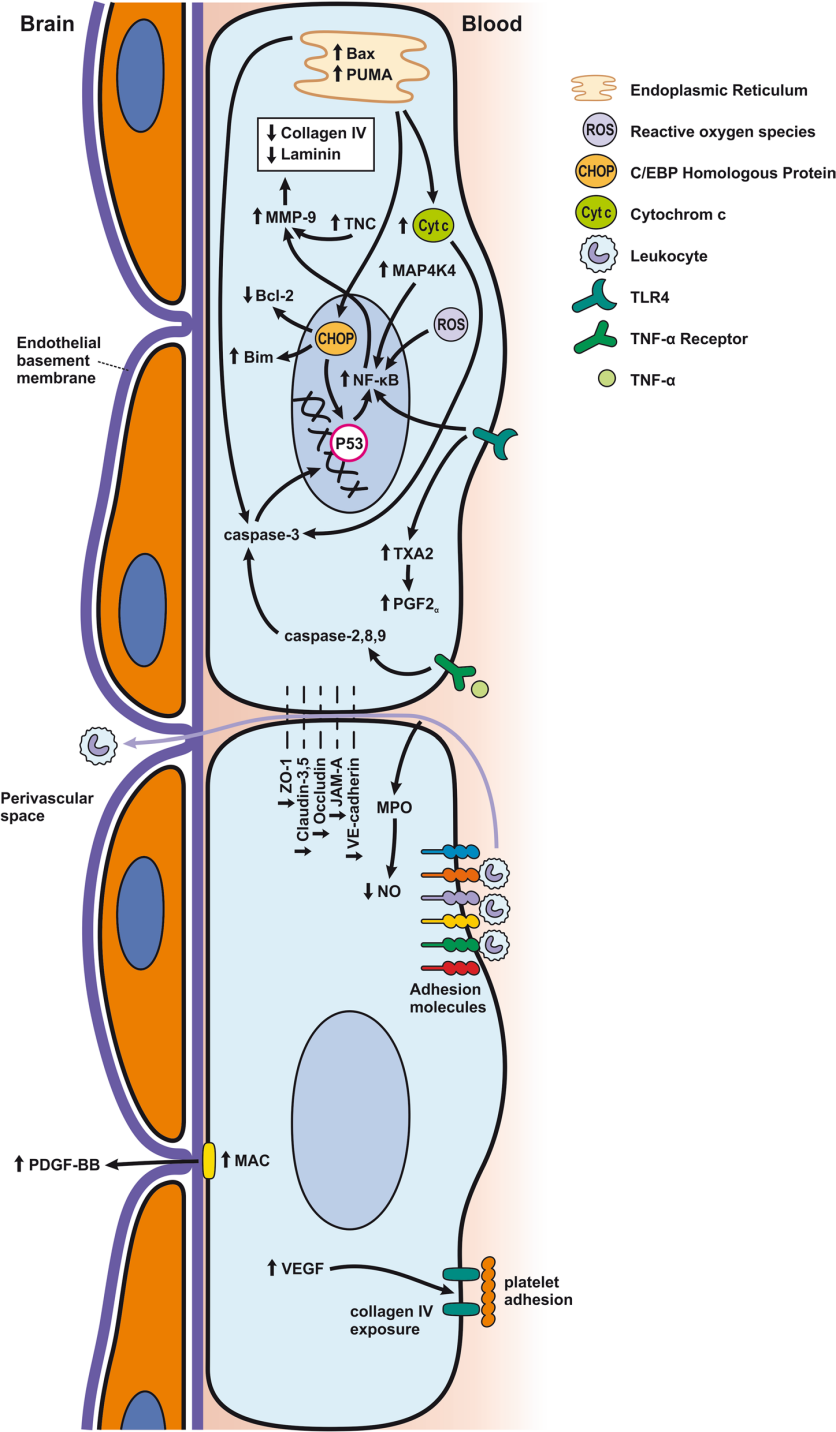

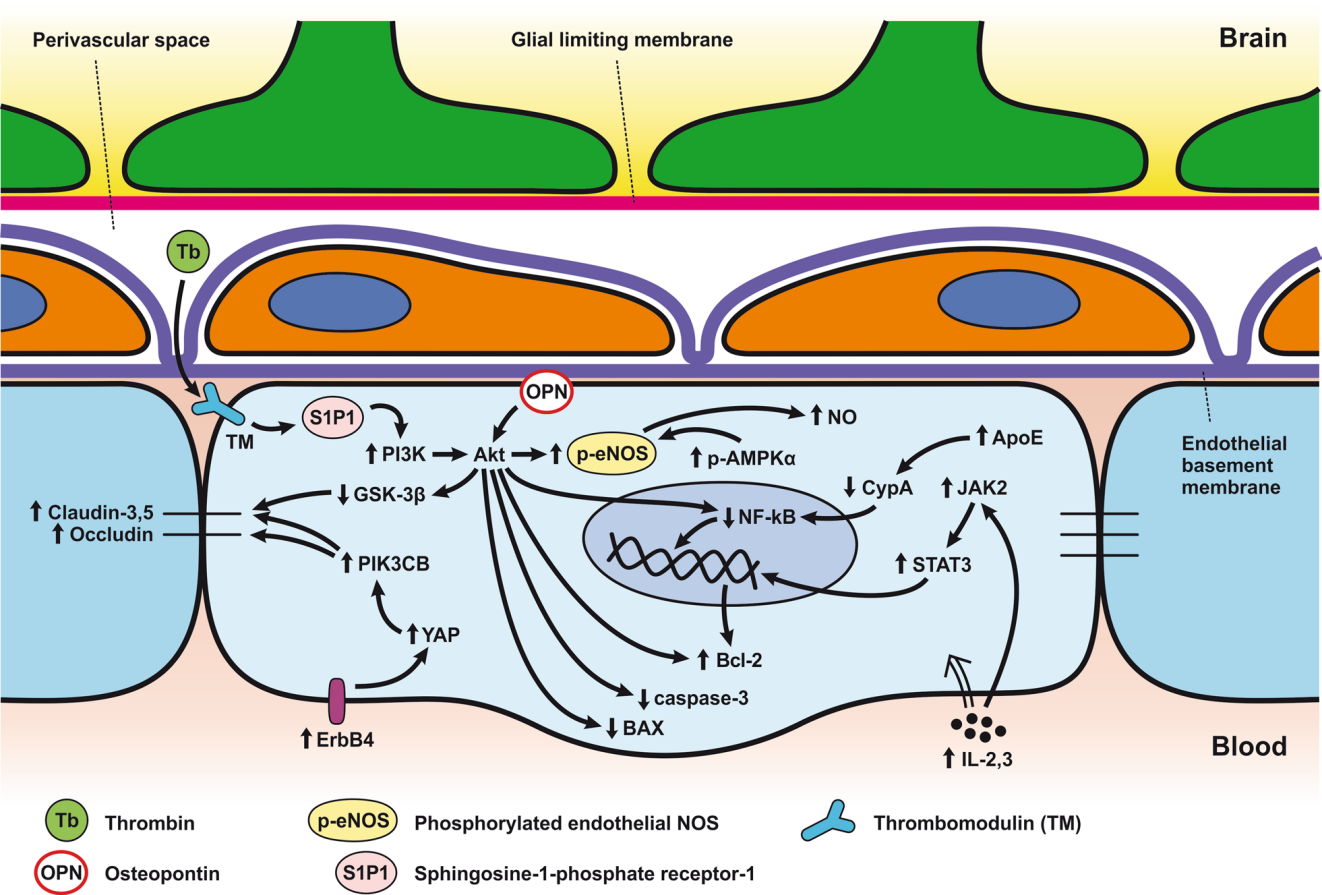
Reaction of endothelial cells to subarachnoid hemorrhage. **a** *ECs disruption after SAH.* BBB dysfunction facilitates the passage of blood components (Hb, Tb and, serum proteins) into the perivascular space. In response to TLR4 activation, p53 and NF-κB are activated, levels of MAP4K4 and ROS are increased, and CHOP is upregulated, resulting in the downregulation of ZO, claudins, JAM, and VE-cadherin, that together increase BBB permeability. ER stress caused by Bax and PUMA upregulation activates caspase-3 and causes DNA fragmentation and cell apoptosis. Caspase-3 activation also accrues via caspase-8 signaling triggered by the TNF-α receptor. CHOP upregulation decreases Bcl-2 expression and upregulates Bim. MMP-9 upregulation reduces collagen IV and laminin proteins in the basal lamina, thus increasing BBB permeability. The upregulation of adhesion molecules promotes leukocyte infiltration, which decreases NO via myeloperoxidase. Cyt c upregulation causes cell death; VSMC contraction is regulated by PGF2α upregulation in response to upregulation of TXA2 and TLR4 activation. ECs are stimulated by MAC, upregulating PDGF-BB production and affecting VSMC. VEGF upregulation leads to collagen IV exposure and thus to platelet adhesion. **b** *ECs protection mechanisms*. Bcl-2 upregulation caused by S1P1/PI3K/Akt and JAK2/STAT3 pathways is due to TM activation and anti-inflammatory cytokine production, respectively. Bcl-2 and STAT-3 upregulation suppress cell apoptosis. Upregulation of OPN activates Akt, decreasing GSK3β expression and TJ protein upregulation. AMPKα upregulation and Akt activation can also increase phosphorylated eNOS, resulting in increased NO and VSMC dilatation. Downregulation of NF-κB, caspase-3, and BAX results from Akt activation. NF-κB is also downregulated by ApoE upregulation and decreased expression of CypA. TJs are upregulated by activation of the ErbB4 receptor, increased Yap, and PIK3CB

The response of endothelial cells to SAH promotes the disruption of BBB and contributes to the development of EBI and cerebral vasospasm (Fig. 3a; Table 1) [147, 148]. Degradation products of erythrocytes such as oxyhemoglobin (OxyHb), excess iron, and oxidative stress contribute to endothelial cell apoptosis that can be observed 24 h after SAH induction [149, 150]. Oxidative stress induces the production of free radicals that cause cellular damage by promoting lipid peroxidation, protein breakdown, and DNA fragmentation. Such changes lead to pathological changes such as vacuolization, breakdown of tight junctions, irregular and flat extensions inside and between endothelial cells, widening of inter-endothelial spaces, cellular apoptosis, necrosis, subendothelial fibrosis, and increased BBB permeability [150–155]. Transmission electron microscopy revealed that the largest openings in the BBB can be seen at 3 and 72 h after SAH which correlates with decreased expression of TJ proteins, ZO-1, and occludin [156]. Severe damage to endothelial cells, including detachment from the basal lamina and cerebral vasospasm (visible by angiography) together, indicate that morphological changes play a key role not only in development of EBI but also in ischemic injury after SAH [157]. These morphological changes, as well as the number of endothelial cells undergoing apoptosis, increase with time following SAH. These changes have been reported to reach a peak on day 5 and 7 after bleeding, which correlates with the development of cerebral vasospasm [158–160]. The number of apoptotic endothelial cells is quite high after SAH [161].

**Table 1:**
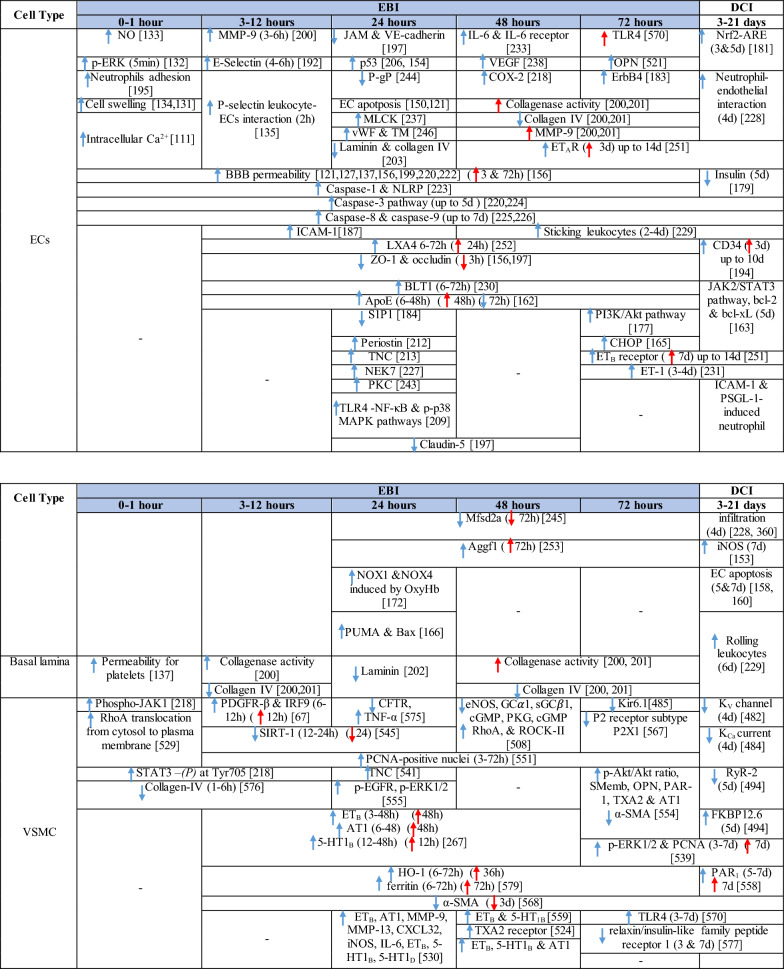

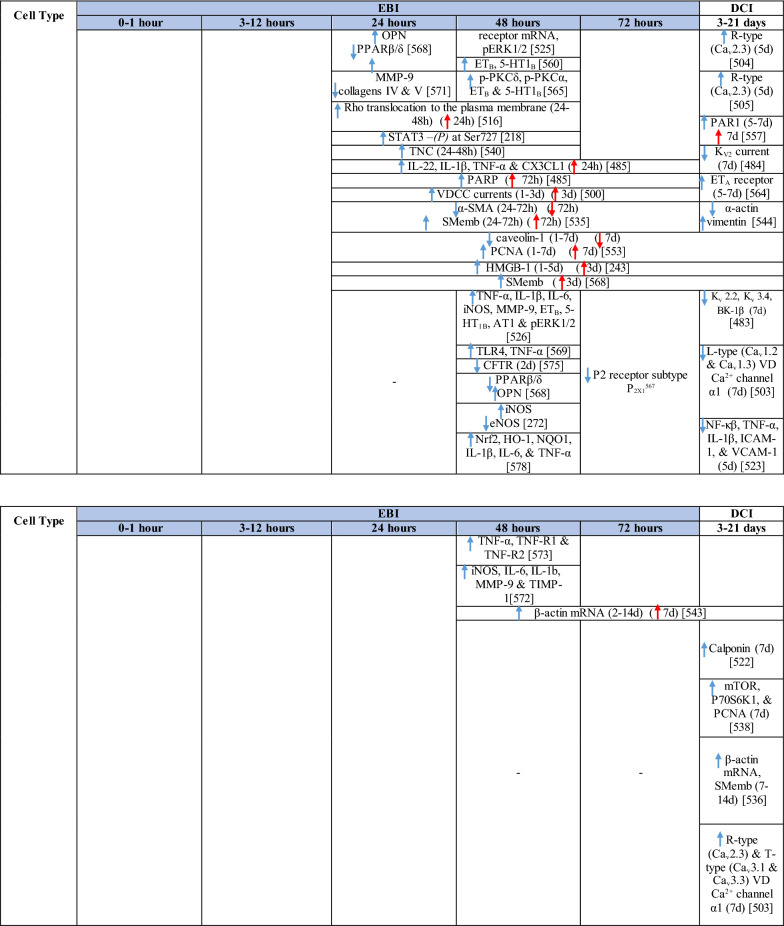

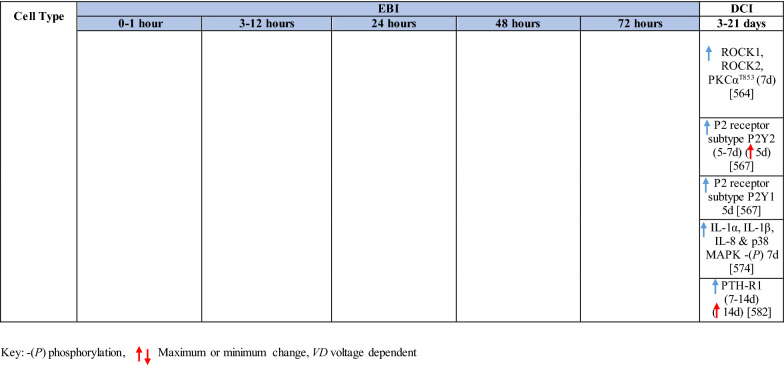
Reaction of endothelial cells, basal lamina and vascular smooth muscle cells to SAH

On the other hand, there are also mechanisms that inhibit cell death in endothelial cells. Levels of ApoE were elevated as early as 6 h following SAH, and this was associated with EBI inhibition; ApoE levels peaked at 48 h and returned to basal levels at 72 h after initial bleeding. ApoE can potentially control BBB integrity by suppressing the inflammatory cyclophilin A (CypA)-NF-κB-MMP-9 pathway [162]. The janus kinase 2 (JAK2)/STAT3 signaling pathway can partially modulate endothelial cell apoptosis as SAH-induced cytokines such as IL-2, IL-3, or IL-6 activate the JAK2/STAT3 cascade leading to increased expression of anti-apoptotic genes like (B-cell lymphoma 2) Bcl-2 and Bcl-xL [163]. JAK2 phosphorylation and activation is initiated early after SAH, peaking on day 3 and gradually decreasing to reach control levels at the 7-day time point [164].

Apoptosis in endothelial cells is orchestrated by endoplasmic reticulum (ER) stress-induced activation of C/EBP homologous protein (CHOP). SAH induces increased CHOP levels, which leads to downregulation of the anti-apoptotic Bcl-2 protein and induction of Bcl-2 interacting mediator of cell death (Bim) [165]. Moreover, increased levels of key pro-apoptotic proteins like p53 upregulated modulator of apoptosis (PUMA) and Bcl-2-associated X protein (Bax) were found in endothelial cells 24 h after SAH. PUMA and Bax were co-localized with glucose-regulated protein 78 (GRP78), a molecular chaperone located in the endoplasmic reticulum (ER) lumen, suggesting that ER stress plays a crucial role in endothelial cell apoptosis. ER affected by PUMA activates the recruitment to the mitochondrial membrane of DRP1, a dynamin-related GTPase, leading to cytochrome c release that results in endothelial cell death [166]. In addition to cytochrome c-induced cell death, PUMA could induce cleaved caspase-3 proteins and thus contribute to apoptosis of endothelial cells after cerebral aneurysm rupture [167]. In support of this, p53 regulated apoptosis-inducing protein 1 (p53AIP1), and cytochrome c were identified on day 7 after SAH [168]. Therefore, p53 seems to be one of the key factors in the control of endothelial cell apoptosis following SAH. Tumor necrosis factor alpha (TNF-α) also plays an important role in apoptosis of endothelial cells after SAH through the action of TNF-α-receptor that activates caspase-2, -3, -8, and -9. Caspase-8 activates caspase-3, which subsequently cleaves poly (ADP)-ribose polymerase (PARP), resulting in DNA fragmentation and cell death [169].

Endothelial cell damage may initiate a thrombogenic state that can worsen ischemia during the cerebral vasospasm following SAH. OxyHb, the superoxide, ferryl, and perferryl ions, along with the hydroxyl and peroxy radicals, may play a vital role in the pathophysiology of the thrombogenic state [170]. The function of xanthine dehydrogenase (XDH), an enzyme present in endothelial cells, is transformed to that of a xanthine oxidase (XO) following SAH. Although XO can produce free radicals like superoxide and hydrogen peroxide, it has been suggested that XO has no significant effect on free radical production following SAH [171]. The generation of oxygen free radicals is promoted by the NADPH oxidases NOX1 and NOX4. OxyHb induces increased levels of NOX1 and NOX4 in endothelial cells 24 h after exposure to OxyHb [172]. NO also plays an important role in free radicals production following SAH. Despite its known vasodilating effect, high NO levels can lead to oxidative injury, lipid peroxidation, inhibition of mitochondrial enzymes, and disruption of gene transcription.

NO production in endothelial cells following SAH Increased levels of inducible nitric oxide synthase (iNOS) were found in endothelial cells, VSMC, adventitial cells, activated microglia, and glial networks. The expression of iNOS corresponded to the distribution of the toxic NO reaction product peroxynitrite, suggesting that iNOS may be the main source of toxic NO products [153]. SAH leads to increased ferritin expression resulting in endothelial cell damage, which contributes to the production of the superoxide anion and acidosis [173]. Moreover, NO synthesized by iNOS increases nitrotyrosine, a marker of peroxynitrite in endothelial cells after SAH. There is evidence that NO produced by iNOS negatively affects the regulatory role of eNOS, decreases NO availability, and contributes to VSMC contraction [174]. Perivascular OxyHb induces the inactivation of Ca^2+^ channels, and the consequent drop in intracellular Ca^2+^ in endothelial cells leads to reduced eNOS expression. Type-V phosphodiesterase (PDE-V), an endogenous inhibitor of eNOS, is also elevated after SAH. It contributes to reduced NO level and thus to the development of vasospasm [175]. Taken together, decreased expression and inhibition of eNOS following SAH can result in reduced NO production, which subsequently contributes to the development of cerebral vasospasm [176]. Activation of the phosphoinositide 3-kinase (PI3K)/Akt pathway led to eNOS activation [177]. Inhibition of eNOS by asymmetric dimethylarginine (ADMA), a likely response to bilirubin oxidation products (BOXes) in the perivascular space, may contribute to the development of cerebral vasospasm. BOXes are eliminated in the later stages of vasospasm, and the decreased ADMA levels leads to increased NO production by endothelial cells [178]. Decreased expression of insulin receptors on endothelial cells probably also has a hand in the reduction of NO and development of cerebral vasospasm after SAH. With its receptors reduced, even insulin– a strong vasoactive molecule—cannot stimulate sufficient NO production in endothelial cells [179].

Osuka et al. found activation of eNOS at Ser^1177^ in the endothelium 1 to 2 days after SAH. Phosphorylation of eNOS was accompanied by increased expression of phosphorylated AMP-activated protein kinase α (p-AMPKα) in endothelial cells suggesting a protective mechanism against mild vasospasm [180].

#### Regulation of tight junctions and adhesion molecules in endothelial cells following SAH

Protective genes like nuclear factor-erythroid 2-related factor 2 (Nrf2) are involved in response to oxidative stress as well as inflammation following SAH. The Nrf2-ARE (antioxidant response element) pathway leads to the expression of several detoxifying enzymes and antioxidative proteins, and as such, is considered a key factor in cytoprotection. The Nrf2-ARE pathway was activated in endothelial cells and VSMC on day 3 and 5 after SAH [181, 182].

Promotion of endothelial cell survival under conditions of oxidative stress is important to preserve BBB integrity (Fig. 3b). SAH induced expression of v-erb-b2 avian erythroblastic leukemia viral oncogene homolog 4 (ErbB4), a kind of epidermal growth factor receptor (EGFR) kinase. Increased ErbB4 expression was found in endothelial cells 72 h after SAH. ErbB4 activates the yes-associated protein (YAP)/PIK3CB (phosphatidylinositol-4,5-Bisphosphate 3-Kinase Catalytic Subunit beta) signaling pathway that increases occludin and claudin-5 expression, reduces brain edema, and contributes to the maintenance of BBB integrity [183].

Sphingosine-1-phosphate receptor-1 (S1P1) proteins modulate the expression of TJ proteins such as claudin-3 and claudin-5. S1P1 activates the PI3K/Akt pathway that inhibits glycogen synthase kinase 3 β (GSK3β) and stabilizes β-catenin resulting in increased claudin-3 and -5 expression. However, S1P1 is mainly localized to endothelial cells and is downregulated 24 h after SAH, resulting in the alteration of TJ protein expression [184]. Thrombomodulin (TM) binds thrombin and catalyzes protein C into APC [185], and S1P1 can also be activated by PAR-1 through the action of endothelial protein C receptor (EPCR) and activated protein C (APC).

Blood in the subarachnoid space stimulates upregulation of adhesion molecules on the luminal surface of endothelial cells such as intercellular adhesion molecule-1 (ICAM-1), vascular cell adhesion protein (VCAM)-1, lymphocyte function-associated antigen-1 (LFA-1), macrophage antigen-1 (Mac-1) as well as endothelial (E)-selectin [186–190]. These molecules are involved in the interaction between endothelial cells and leukocytes that mediate the recruitment, adhesion, and transmigration of white blood cells to the site of hemorrhage [191–193]. CD34, a transmembrane glycoprotein, plays a key role in the attachment of leukocytes to the endothelial cells, as well as in the recruitment of monocytes and macrophages to the site of injury. Increased expression of CD34 was found in parallel with cerebral vasospasm, which peaks on day 3, and it decreased to values similar to controls on day 10 following SAH [194]. However, neutrophil adhesion on endothelial cells and neutrophil infiltration of the brain begins in the first 10 min after SAH. Early neutrophil infiltration correlates with decreased cerebral NO levels by the action of the neutrophil-derived enzyme myeloperoxidase, which degrades NO 10 min after SAH. Adherent and infiltrating neutrophils contribute to BBB damage after SAH by releasing reactive oxygen species (ROS), elastases, proteases, collagenase, and MMP-9 [195].

Activation of the NF-κB inflammatory pathway facilitates disruption of TJ between endothelial cells, which is considered to be the main cause of post-hemorrhagic vasogenic edema [196]. Experimental studies describing changes in the expression of TJ proteins as one of the causes of EBI have focused primarily on the first 24 h following SAH. Generally, experiments using endovascular perforation or direct injection of blood into CSF showed decreases in TJ protein ZO-1, occluding, claudin-5, JAM-A, and adherent junction protein VE-cadherin 24 to 48 h following SAH [185, 197]. Despite these findings, there is also some evidence of biphasic changes in ZO-1 and occludin expression with the lowest expression values at 3 h after SAH followed by a partial recovery and subsequent decrease 72 h after SAH. Moreover, decreased expression of TJ proteins was correlated with increased permeability peaking at 3 and 72 h after SAH [156]. The assumption that BBB permeability increases early after SAH is supported clinically as T2-weighted MRI hyperintensities can be seen 4 h after SAH induction [198]. However, experimental studies proved that increased microvascular permeability occurs already in the first few minutes after SAH [137, 199]. One of the pathophysiological cascades that lead to BBB disruption is perturbation in the microvascular basal lamina mediated by loss of collagen IV after SAH. While the greatest increase of MMP-9 and collagenase activity occurs 3 to 6 h after induction of SAH [189, 200], it appears that collagen IV expression decreases in two phases. The first decrease happens in 3–6 h as described above, and the second after 48 -72 h suggests delayed microvascular damage after SAH [200, 201]. This biphasic decrease of collagen IV expression is consistent with biphasic changes in the expression of the TJ proteins ZO-1 and occludin, as was described above [156]. Moreover, laminin, one of the main components of the basal lamina as well as the substrate for MMP-9, also decreases at 24 h after SAH [202, 203]. The combined reduction of laminin, occludin, and collagen IV correlates with the upregulation of MMP-9 in endothelial cells 24 h after SAH [154, 204]. Moreover, it was suggested that MMP-9 induced laminin degradation could play a role in the apoptosis of endothelial cells following SAH [203]. In addition, increased microvascular collagenase also contributes to the loss of collagen IV [200, 201]. The expression of JAM-A is decreased after SAH [205], and MMP‐9 has been reported to play an important role in JAM-A degradation [206]. Yan et al. suggested that the p53—NF-κB—MMP-9 molecular signaling pathway is involved in the pathophysiological cascades inside cerebral endothelial cells after SAH. Inflammation is an important factor in the progression of BBB disruption. This assumption is supported by increased expression of toll-like receptor (TLR)-4 and high-mobility group box 1 (HMGB1) following induction of SAH [207, 208]. Increased expression of p53 induced the up-regulation of MMP-9 via NF-κB and was recorded in brain endothelial cells 24 h after SAH, which leads to the degradation of occludin and disruption of basal lamina through the degradation of collagen IV and laminin [154, 206]. Inflammatory-induced degradation of TJ proteins contributes to vasogenic brain edema 24 h following SAH [209]. Cortical endothelial cells overexpress mitogen-activated protein kinase 4 (MAP4K4), whose upregulation leads to increased expression of phosphorylated NF-κB and MMP-9 and the subsequent degradation of ZO-1 and claudin-5, resulting in BBB disruption [210]. MicroRNA (miR)-630 may also play a role in the expression of adhesion molecules and TJ proteins. Low miR-630 expression was found in endothelial cells treated with arterial blood, indicating a crucial role for exosomal miR-630 in maintaining BBB integrity after SAH [211]. Periostin, one of the matricellular proteins, activates the MAPK signaling pathway through integrins and modulates downstream pathways such as MMP-9 after SAH. Following SAH, the level of periostin was increased in capillary endothelial cells 24 h after bleeding [212]. Tenascin-C (TNC), a member of the matricellular protein family, regulates mitogen-activated protein kinase (MAPK) activation in endothelial cells after SAH. Activation of MAPK leads to the induction of MMP-9, resulting in ZO-1 degradation. Expression of TNC was upregulated in endothelial cells 24 h after SAH [213]. Increased expression of osteopontin (OPN) was found in endothelial cells as well as in astrocytes. OPN induction peaked 72 h after SAH and was associated with the restoration of the BBB. OPN increases MAPK phosphatase-1 (MKP-1) acts as an inhibitor of VEGF-A, phospho- Jun N-terminal Kinase (JNK), phospho-p38, and phospho-extracellular signal-regulated kinase (ERK)-1/2, and thus contributes to the stabilization of the BBB [214]. OPN also induces the activation of p-Akt and inhibits apoptosis through reduced expression of cleaved caspase-3 and Bax while increasing the level of anti-apoptotic Bcl-2 [215]. The Rho-ROCK (Rho-associated protein kinase)/MAPK, as well as the tyrosine kinase cascades, are activated and lead to proliferation of VSMC and vascular contraction. Activation of the Rho-ROCK/MAPK pathway in VSMC occurs through the upregulation of platelet-derived growth factor β receptor (PDGFR-β) by prolonged contact with PDGF-BB. Endothelial cells are stimulated by the C5b–9 membrane attack complex (MAC) and upregulate the production of PDGF-BB that affects the VSMC after SAH [216]. TLR4 activation also upregulates cyclooxygenase-1 (COX-1) in endothelial cells after SAH, and the activation of COXs catalyzes the conversion of arachidonic acid to prostaglandin H2 and subsequent metabolites like thromboxane A (TXA2), prostaglandin F2α, and prostacyclin leading to VCMC contraction [217]. COX-2 expression in endothelial cells and VSMC also increased at 2 days after SAH. The pro-inflammatory cytokine interleukin (IL)-6 in the CSF activates the JAK-STAT signaling cascade and upregulates transcription of early genes, including COX-2 [218]. It was suggested that induction of COX-2 after SAH could lead to a synthetic shift from vasodilating prostaglandins (PGI_2_ and PGE_2_) to pro-constriction eicosanoids like PGH_2,_ PGF_2α,_ and TXA2 [151, 219].

The biochemical events associated with BBB injury occur in the first few minutes following SAH. These alterations include caspase-3 activation and collagen-IV depletion, which lead to endothelial cell damage and microvascular basal lamina interruptions [137, 220–222]. In addition, caspase-1 as well as leucine-rich repeat (LRR)-containing protein 3 (NLRP3) and apoptosis-associated speck-like protein containing a CARD (ASC) are increased in the endothelial cells in the first 3 days after SAH. Activation of NLRP3 leads to the maturation and secretion of proinflammatory molecules such as IL-1β and IL-18 [223]. More numerous cleaved caspase-3 positive endothelial cells were found as early as 10 min after SAH induction [220]. In addition, increased caspase-3 expression was found in endothelial cells up to 5 days after SAH, suggesting long-lasting damage to the BBB [224]. Moreover, caspase-8 and caspase-9 were elevated during the first few days following SAH, and this elevation lasted for 7 days. Higher caspase-8 and caspase-9 were accompanied by increased BBB permeability on day 7 after SAH [225, 226]. The serine/threonine protein kinase 7 (NEK7) has an essential role in the activation of the NLRP3 inflammasome. NEK7 induces neuronal apoptosis, and its expression was found mainly in endothelial cells as well as in microglia, peaking at 24 h after SAH [227]. The endothelium acts as a pathway for the transfer of proinflammatory cells resulting in the development of inflammatory reactions following SAH. The neutrophil-endothelial interaction manifests as spreading cerebral inflammation, starts shortly after SAH, with the highest extent around day 4 after SAH. Increased expression of adhesion molecules like P-selectin and intercellular adhesion molecule 1 (ICAM-1) is required for neutrophil-endothelial interaction and the development of intraparenchymal inflammation [228]. Higher numbers of rolling leukocytes were seen on day 6, as were higher numbers of adherent leukocytes between day 2 and day 4 after SAH, suggesting that neutrophils play an important role in the development of neuroinflammation in the first few days following SAH [229]. However, it seems that cerebrovascular inflammation mediated by the P-selectin leukocyte-endothelial cell interaction occurs as early as 2 h after SAH. A sudden increase in ICP might be among the most important factors initiating leukocyte-endothelial interactions and the inflammatory response following SAH [135]. The LTB4-BLT1-NF-κB axis resulting in up-regulation of adhesion molecules such as ICAM-1 and vascular cell adhesion protein 1 (VCAM-1) may play a role in the attachment of leukocytes to endothelial cells and their trans-endothelial migration. Immunostaining showed increased expression of the LTB4 receptor 1 (BLT1) in endothelial cells, neurons, and microglia starting at 6 h, peaking at 24 h, and lasting for 3 days after SAH [230]. ICAM-1 and VCAM-1 are upregulated by pro-inflammatory cytokines like TNF-α as well as IL-1, which activate NF-κB and activator protein 1 (AP-1), a transcription factor that initiates cytokine expression [231]. Higher levels of IL-6 in endothelial cells also induce a pro-inflammatory reaction [232], and overexpression of IL-6 and its receptor was found in BBB endothelial cells. Up-regulation of IL-6 is potentiated by an autocrine mechanism 2 days after induction of SAH [233]. Activation of NF-κB can be induced by Ca^2+^ oscillation between Ca^2+^ uptake and release through the action of ER Ca^2+^-ATPase and inositol trisphosphate (IP_3_)-dependent Ca^2+^ channels. Oscillation in intracellular Ca^2+^concentrations leads to increased VCAM-1 expression and endothelial cell shrinkage [234]. These pro-inflammatory cascades may play an important role in the development of the neurovascular inflammatory reaction following SAH leading to poor functional outcomes [235, 236].

#### Contribution of endothelial cells to EBI and vasospasms following SAH

The endothelial cytoskeleton may also play a critical role in BBB integrity. Upregulation of myosin light chain kinase (MLCK) leads to increased phosphorylation of myosin light chain (MLC), resulting in cytoskeletal rearrangement, reduced endothelial cell–cell contact, loss of BBB integrity, and the development of vasogenic brain edema following SAH [237]. Moreover, endothelial tight junctions prevent platelets from adhering to extracellular collagen, which helps maintain the hemostatic/thrombotic balance. This balance is disturbed by increased expression of endothelial vascular endothelial growth factor (VEGF) induced by hypoxia during vasospasm between 24 and 72 h after SAH [238–240]. The upregulation of VEGF leads to collagen IV exposure and binding to glycoprotein Ia-II located on platelets resulting in platelet adhesion and disruption of endothelial TJ in the acute phase of SAH [199, 241]. These changes lead to platelet penetration into the brain, which initiates neuroinflammation and EBI after SAH [137, 220, 222]. Moreover, altered NO production in endothelial cells is insufficient to inhibit platelet adhesion and aggregation, and this contributes to ischemic brain injury as one of the major complications after SAH [242].

Enhanced expression of protein kinase C (PKC) is considered to be one of the main mechanisms contributing to the development of vasospasms. The PKC family is classified based on differences in structure and substrate requirements into conventional or Ca^2+^ dependent PKCs (α, βI, βII and γ), novel or Ca^2+^ independent PKCs (δ, ε, η and θ) and atypical PKCs (ζ and ι/λ). The expression and location of PKCη correlate with the S100 calcium-binding protein B (S100B), and PKCβ is accompanied by the calcium-binding S100 protein A1 (S100A1). The co-expression of these S100 proteins suggests that these proteins indirectly activate PKC during cerebral vasospasm after SAH [243].

Transport mechanisms across endothelial cells are also altered after SAH. However, little is known about these mechanisms affecting endothelial cell transport following SAH. P-glycoprotein (P-gP), one of the major efflux transporters at the BBB endothelium, decreases after SAH [244]. Vesicular trafficking in the endothelial cells also plays a role in BBB permeability. Activation of Mfsd2a inhibits caveolae formation and subsequent transcytosis across the endothelial cell. Mfsd2a expression reaches its lowest level at 72 h after SAH and this, in addition to changes in TJ proteins, contributes to increased transport across the BBB after SAH [245].

Von Willebrand factor (vWF), and thrombomodulin (TM), and endothelin 1 (ET-1) were considered as the “gold standard” for evaluating BBB integrity. The increased expression of vWF, TM, as well, as ET-1 indicates a disrupted BBB following SAH [246]. Moreover, TM could protect endothelial TJ proteins following SAH by inhibiting the p38 MAPK-p53/NF-κB (p65) pathway [185].

Endothelial cells affect VSMC through increased expression of ET-1 following stimulation with OxyHb or erythrocyte lysate [247]. Elevated levels of ET-1 were associated with a degree of angiographic vasospasm after SAH. ET-1 binds to the ET_A_ receptor of vascular smooth muscle cells, activates the ERK1/2 pathway and the Kruppel-like transcription factor 4 (KLF4). Activation of KLF4 induces the transformation of VSMC from the contractile to the synthetic phenotype [248, 249]. Increased expression of ET-1 peaks 3–4 days after SAH, and its expression is followed by negative feedback via the activation of eNOS, resulting in vasodilatation [231]. Increased NO levels have an attenuating role and inhibit ET-1 production [250]. ET-1 binds to the ET_A_ receptor, which is increased in endothelial cells at 2 days, peaks at day 3, and remains elevated till day 14. Similarly, endothelin B (ET_B_) receptor increased on day 3, peaked at day 7, and remained elevated until day 14 following SAH [251].

One of the ways by which endothelial cells inhibit neutrophil infiltration and suppress the expression of pro-inflammatory cytokines is through lipoxin A4 (LXA4). However, the expression of LXA4 decreased in endothelial cells after SAH starting at 6 h, peaked at 24 h, and lasted for 3 days after bleeding. LXA4 inhibits the phosphorylation of ERK1/2 via FPR2, leading to the modulation of the NF-κB pathway and resulting in decreased levels of proinflammatory cytokines like TNF-α, IL-6, IL-1β, intercellular adhesion molecule 1 (ICAM-1), and neutrophil infiltration [252]. The number of phosphorylated ERK-positive endothelial cells increased after SAH [132]. The angiogenic factor with G patch and FHA domains 1 (Aggf1) may play an important role in regulating endothelial TJ proteins and proinflammatory cytokines after SAH. The expression of Aggf1 is upregulated mainly in endothelial cells, astrocytes, and microglia in the cerebral cortex over the first few days following SAH. Aggf1 activates PI3K/Akt pathway, which leads to decreased NF-κB p65 phosphorylation [253].

### The response of pericytes to SAH

#### SAH induces pericyte contraction

**Fig. 4:**
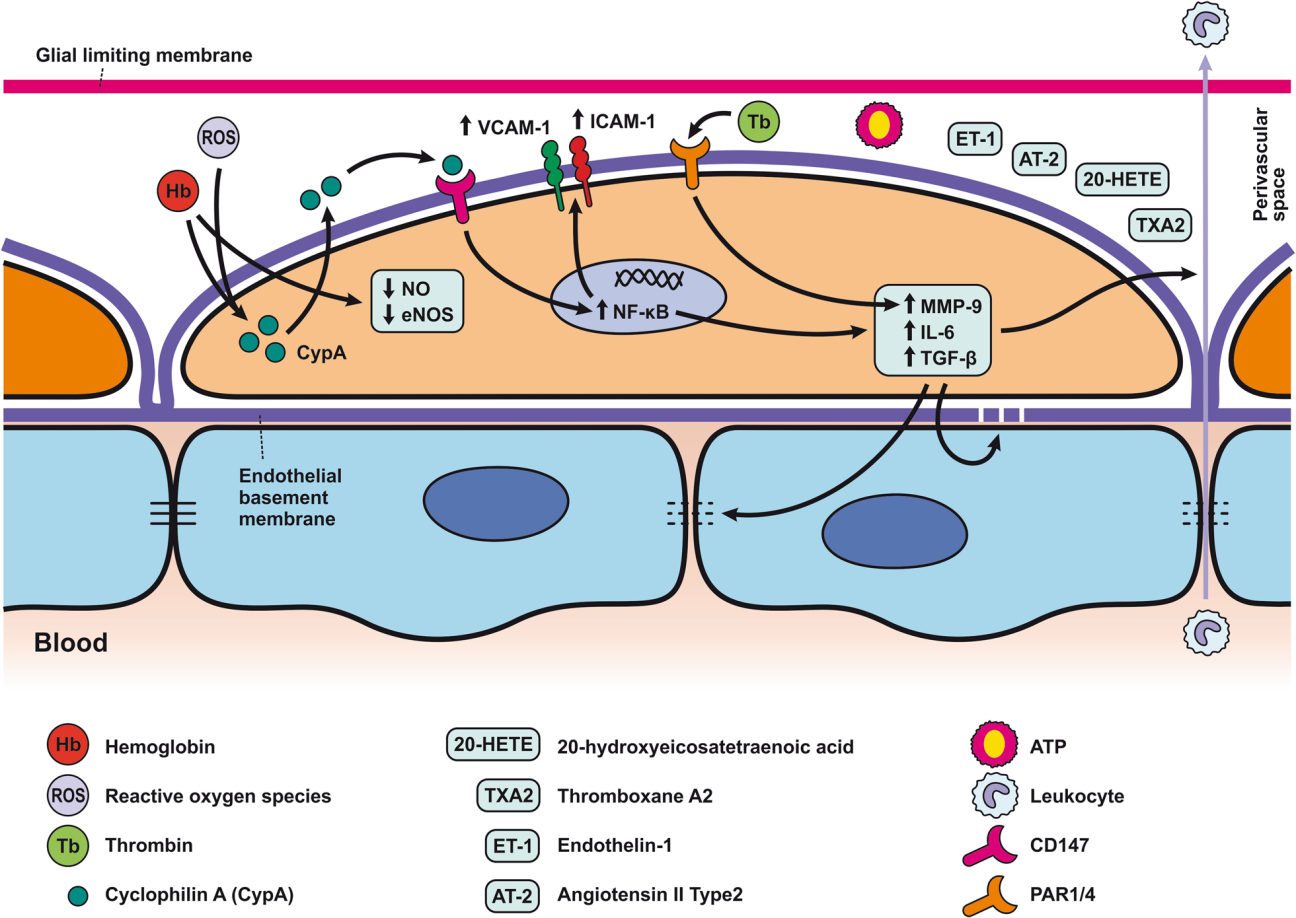
Reaction of pericytes to SAH. Pericytes are in direct communication with ECs, and thus, pericyte contraction in response to SAH can alter BBB integrity. Pericytes are exposed to high concentrations of Hb and other substances such as ET-1, AT2, 20- 20-HETE, TX2, and ATP present in the CSF after SAH that have a predominant constrictor effect. CypA release, caused by Hb, and ROS, can activate CD147, which activates the NF-κB pathway, causing increased expression of MMP-9 and pro-inflammatory cytokines such as IL-6 and TGF-β, as well as upregulation of adhesion molecules (ICAM-1 and VCAM-1). MMP-9 is also upregulated via activation of PAR1/4 by Tb. Upregulation of MMP-9 and elevated cytokines results in the degradation of basal lamina and TJ proteins, thus increasing BBB permeability, allowing leukocyte penetration into the CNS

Pericytes are one of the main BBB components localized between the endothelial cells and the astrocytic endfeet [254]. Pericytes are involved in the complex post-SAH pathophysiology due to their pleiotropic roles such as contractile function, immune or phagocytic function, stem cell potential, and angiogenesis (Fig. 4; Table 2) [136]. Pericytes regulate cerebral blood flow by controlling microvascular diameter at the capillary level [255]. Moreover, pericytes are able to transform into alpha-smooth muscle actin (α-SMA) under pathophysiological conditions such as after SAH and accelerate capillary lumen constriction [256, 257]. The α-SMA phenotype of pericytes regulates BBB integrity by secreting barrier integrity-reducing factors like vascular endothelial growth factor (VEGF), MMP-9, and MMP-2 [258, 259]. Hb released from lysed erythrocytes reaches the pericytes through perivascular spaces and causes microvascular constriction via NO scavenging early after SAH. NO acts as a pericyte dilator, and a decrease in NO levels contributes to pericyte contraction after SAH. However, pericyte contraction persists into the later phase of SAH and is caused by decreased eNOS expression [260, 261]. While pericyte contraction is followed by pericyte dilatation, dilated pericytes nevertheless do not reverse blood flow. We call this reaction of pericytes the “no-reflow phenomenon” [106].

**Table 2:**
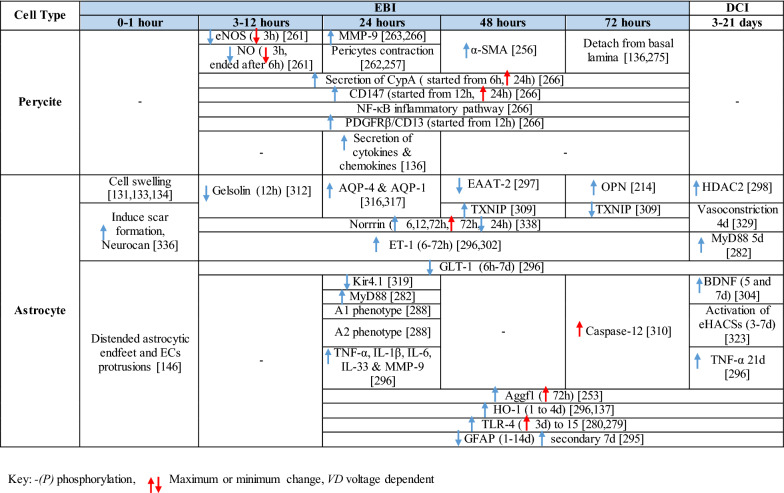
Reaction of pericytes and astrocytes to SAH

#### Inflammatory reaction in pericytes following SAH

The expression of MMP-9 by pericytes seems to be extremely high when compared to the high levels seen in astrocytes and endothelial cells in response to thrombin in the CSF following SAH [262–264]. Thrombin activates protease-activated receptors (PARs) on pericytes such as PAR1 and PAR4, leading to the activation of G coupled proteins and both the PKCθ-Akt and the PKCδ-ERK1/2 pathways resulting in increased expression of MMP-9 [263, 265].Apart from thrombin, reactive oxygen species (ROS) generated in the brain after SAH may also activate the NF-κB inflammatory pathway and induce MMP-9 expression. Cyclophilin A (CypA), secreted by pericytes, is likely to play a major role in this pathophysiological cascade. Increased expression of CypA was found between 12 and 72 h after SAH and was co-localized with pericyte markers such as lectin and PDGFRβ/CD13. Autocrine and paracrine activation of CD147 by CypA leads to the activation of the downstream NF-κB inflammatory pathway [266]. Oxidative stress and nitrative stress, including peroxynitrite formation induced by microvascular walls, together lead to a sustained increase in intracellular calcium level resulting in pericyte contraction, narrowing of capillaries, entrapment of erythrocytes, thus hampering microcirculation [257, 262]. Pericytes are exposed to high concentrations of Hb and other contractile substances in the CSF after SAH, such as endothelin 1 (ET-1), AT2, 20-hydroxyeicosatetraenoic acid (20-HETE), TX2, and ATP that have a predominantly constricting effect [146, 267–271]. On the other hand, prostacyclin, epoxyeicosatrienoic acid (EET), as well as adenosine released after SAH have a predominantly vasodilatory effect on pericytes [269, 272–274]. Capillary constriction occurs near the apoptotic mural cells considered to be pericytes based on their PDGFRβ expression, indicating an important role for them in regulating blood flow following SAH [262].

Excess of ferritin was co-localized within pericytes as well as endothelial cells and astrocytes 3 days after SAH. This suggests that pericytes store iron after SAH and thus contribute to low oxygen tension, high levels of reactive oxygen species (ROS), and acidosis. The non-heme iron can be released from ferritin only after reduction to Fe^2+^ under the acidic conditions that occur in extracellular fluid. In this form, Fe^2+^ accelerates ROS production. These conditions include SAH as well as ischemia as they lead to electrolyte imbalance and decreased pH in extracellular fluid [173]. It was found that pericytes could detach from the basal lamina and migrate into the perivascular space, where they are indistinguishable from perivascular macrophages and reactive microglia [136, 275]. DAMPs in the perivascular spaces act as antigens with the ability to activate pericytes. This leads to a local pro-inflammatory response characterized by the increased expression of intercellular adhesion molecule 1 (ICAM-1), vascular cell adhesion protein 1 (VCAM-1), cytokines, and chemokines, including IL-6 and TGF-β, which contributes to the infiltration of leukocytes and the degradation of TJs and other molecules such as sphingosine-1 phosphate (S1P) and glycosaminoglycans (GAG) [136].

### Response of astrocytes to SAH

**Fig. 5:**
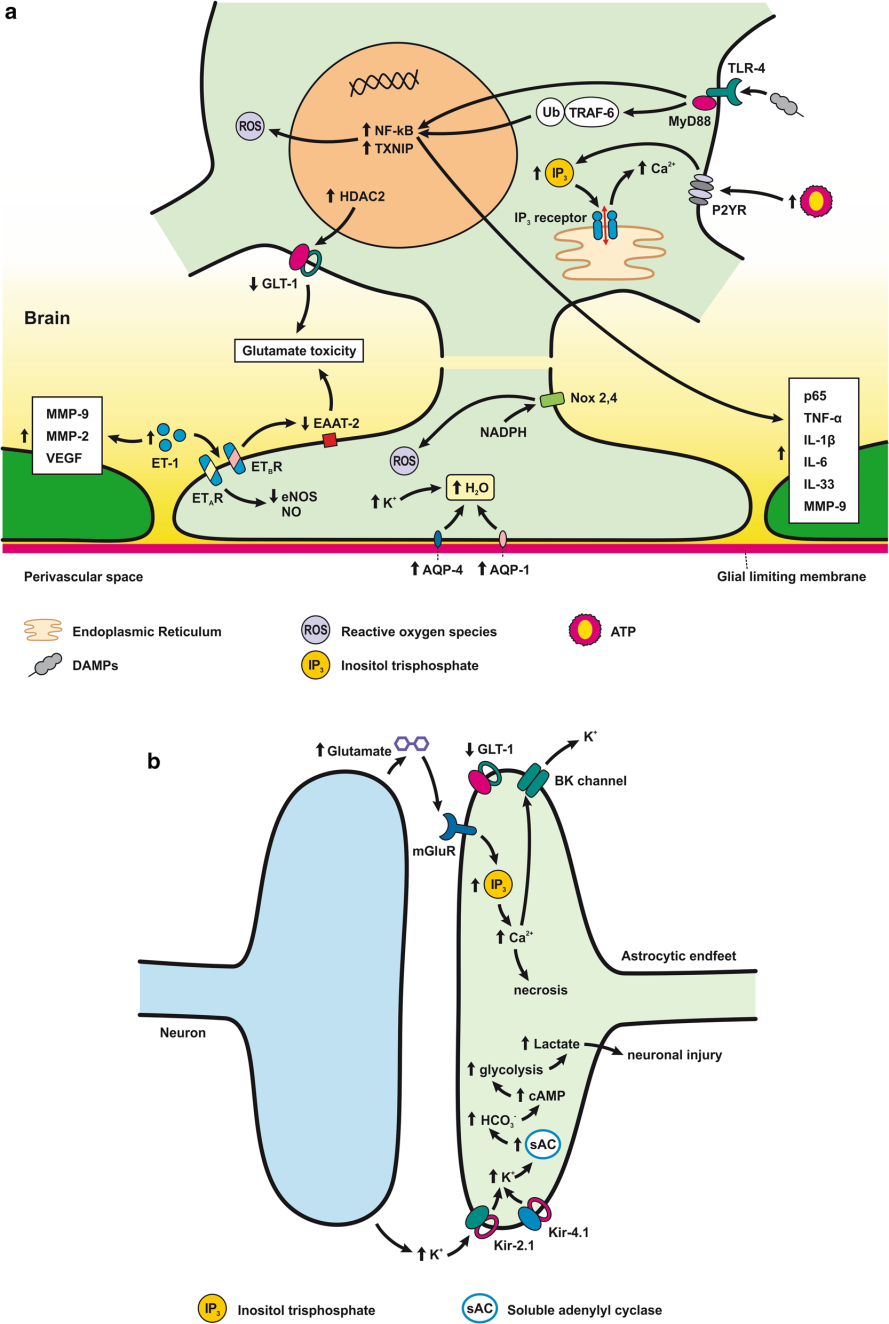
Reaction of astrocytes to SAH. **a** *Astrocyte-ECs interaction after SAH*. Extracellular ATP activates P2Y receptors leading to IP3-dependent Ca^2+^ release and astrocyte necrosis. Activation of TLR4/MyD88 pathway leads to TRAF6 ubiquitylation and NF-κB upregulation, promoting ROS production. TXNIP can also promote cell death by inducing ROS production. ROS is increased by upregulated NOX as well. Glutamate toxicity results from GLT-1 dysfunction due to upregulated HDAC2. ET-1 released by ECs activates ETB receptors, which downregulate the EAAT-2 transporter, causing glutamate toxicity. ET-1 activates ATA receptors and causes K^+^ channel dysfunction by decreasing eNOS and NO production. Another effect of ET-1 is to upregulate MMP-2, MMP-9, and VEGF, thus altering BBB permeability. Brain edema is caused by water accumulating inside the astrocyte. Accumulation of K^+^ and upregulated AQP-4 and -1 are mainly responsible for water accumulation, leading to cell swelling and apoptosis. **b** *Astrocyte-neuron interaction after SAH*. Neuronal activity following SAH increases the K^+^ level in the synaptic cleft. The released K^+^ activates Kir2.1 and Kir4.1 channels that then import K^+^ into the astrocytes. K^+^ increases VSMC contraction and cell swelling. sAC in astrocytes is activated by increased extracellular K^+^ and mediates HCO_3_^−^ entry into astrocytes. Increased HCO_3_^−^ levels trigger the cAMP cascade causing initiation of glycolysis and lactate formation, leading to neuronal injury. Inhibition of GLT-1 leads to glutamate toxicity. mGluR activation in astrocytes stimulated by neuronal activity leads to IP_3_ activation and elevated Ca^2+^ levels in the astrocyte and K^+^ efflux via BK channels. Increased levels of Ca^2+^ could lead to cell necrosis. Increased concentration of K^+^ and glutamate causes spreading depolarization

Morphological changes in astrocytes following SAH include distended astrocyte end-feet and endothelial protrusions that compress the capillary lumen (Fig. 5a; Table 2) [146]. Astrocyte deformations include distortion of the foot processes anchored to the basement membrane that leads to disruption of cerebral ultrastructure [276]. 4 days after SAH induction, hippocampal astrocytes showed cell body swelling, retraction of processes, and reduction in capillary coverage of AQP-4 positive astrocytic endfeet. Morphological changes in hippocampal astrocytes disrupt astrocyte-capillary interactions and thus contribute to the development of long-term cognitive dysfunction following SAH [277].

Like pericytes, astrocytes too respond to DAMPs from the perivascular space and promote the expression of pro-inflammatory cytokines, chemokines, growth factors, as well as recruitment and activation of peripheral immune cells [278].

TLR4 plays an important role in neuroinflammation progression. It appears that blood degradation products such as heme and probably other DAMPs from lysed blood cells are able to activate the TLR4 receptor, whose expression was found also in astrocytes [279–281]. Increased expression of TLR4 in astrocytes leads to worsened neuroinflammation, an observation confirmed by the overexpression of myeloid differentiation primary response protein 88 (MyD88). MyD88 acts as an adapter essential for TLR signal delivery down to NF-κB in astrocytes but also in microglia at 1 and 5 days following SAH induction [282]. Activation of the TLR4/MyD88 pathway leads to ubiquitylation of tumor necrosis factor receptor-associated factor 6 (TRAF6), which can then translocate to mitochondria and promote ROS production. Ubiquitination of TRAF6 may increase the degradation of ULK1, an enzyme important for autophagy, or reduce its phosphorylation, thus inhibiting autophagy, which can exacerbate brain injury after SAH [283, 284]. In addition to astrocytes, increased expression of TLR4 expression was found also in neurons, microglia and VSMC 24 h following SAH induction [208]. This finding suggests that not only astrocytes, but also other cellular components of the neurovascular unit play an important role in the development of TLR4-induced neuroinflammation.

In addition to TLR4/MyD88/TRAF6 pathway, specific enzymes from the NOX family also contribute to mitochondrial ROS formation. Increased expression of NOX 2 and NOX 4 proteins was found in astrocytes after SAH. These proteins transfer electrons from NADPH to oxygen molecules and generate ROS [172].

NF-κB activation via the MyD88-dependent TLR4 signaling pathway leads to the expression of p65, TNF-α, and IL-1β. Expression of these proinflammatory molecules may be reduced by activating the PI3K/Akt signaling cascade, a molecular pathway downstream of angiogenic factor with G-patch and FHA domain 1 (Aggf1) action. Increased level of Aggf1 was found mainly in astrocytes, endothelial cells as well as in microglia 24 h and peaked 72 h after SAH, indicating an important role for the PI3K/Akt signaling cascade in the first days after bleeding [253].

#### SAH induced polarization of astrocytes

In a stimulus-specific manner, astrocytes can be divided into the pro-inflammatory/harmful A1 phenotype and the anti-inflammatory/beneficial A2 phenotype [285]. Activated microglia following SAH induces A1 polarization by secreting proinflammatory cytokines such as IL-1α or TNFα [286, 287]. TNFα released from activated microglia activates in its turn the NF-κB pathway resulting in the differentiation of astrocytes into the harmful A1 phenotype. However, TNFα may also play a protective role by inducing neuronal-derived prokineticin 2 (PK2) expression that activates the STAT3 cascade, thereby promoting the beneficial A2 astrocytic phenotype [288]. Increased immunoreactivity of astrocytic TNFα was observed mainly in brain tissue in the vicinity of the cerebral arteries in the first 24 h following SAH induction [289].

Astrocytic as well as microglial activation was detected by the neuroinflammatory biomarker 18-kDa translocator protein (TSPO) using PET with a specific [18F]DPA-714 tracer. The degree of astrocytic and microglial activation and neuroinflammation correlated with the severity of SAH [290]. Long-lasting astrocytic activation is accompanied by a chronic inflammatory response and may contribute to the formation of scar tissue and neuronal dysfunction that were observed 21 days after SAH [291]. Scar formation is a cell-specific process involving astrogliosis characterized by increased expression of two astrocyte-specific proteins, S100 calcium-binding protein B (S100B) and glial fibrillary acidic protein (GFAP). S100B expression increases following SAH, and S100B binds to advanced glycation end products (RAGE), leading to the stimulation of NF-κB-dependent expression of proinflammatory molecules [292, 293]. In addition to neuroinflammation, S100B induces oxidative stress that can promote neuronal death and damage cerebral vascular reactivity [294]. GFAP is a highly specific marker for astrocytes, and its concentration in CSF was altered following SAH. GFAP levels decreased gradually over the first 14 days following SAH, with a temporary increase on day 7. This likely coincides with cerebral vasospasm and the subsequent ischemia and acidosis to which astrocytes are more vulnerable than neurons [295].

Glutamate reduction by astrocytes is impaired following SAH Reactive astrocytes have a reduced ability to detoxify glutamate from the synaptic cleft (Fig. 5b). This phenomenon is caused by the downregulation of glutamate transporter 1 (GLT-1) and EAAT-2 on the astrocytic membrane, which leads to neuronal damage [296].

Down-regulation of EAAT-2 in astrocytes following SAH is one of the important mechanisms causing glutamate excitotoxicity and neuronal damage. The SAH-induced decrease in Akt phosphorylation leads to lowered expression of astrocytic EAAT-2 [297], with histone deacetylase 2 (HDAC2) playing an important role in the alteration of GLT-1. Increased expression of HDAC2 in astrocytes after SAH causes histone deacetylation and inhibition of GLT-1 expression leading to long-term accumulation of glutamate in the synaptic space, which results in dephosphorylation of ionized glutamate receptors GluA1 as well as GluN2B on the postsynaptic membrane. These changes may negatively regulate hippocampal synaptogenesis and contribute to cognitive impairment, frequently occurring after SAH [298]. Despite the downregulation of astrocytic glutamate transporters after SAH, astrocytes are capable to take up glutamate and convert it to glutamine via the enzyme glutamine synthetase. Glutamine synthesis represents astrocytic metabolic activity, and this energy demand can be a hindrance during ischemia after SAH. Increased interstitial glutamine correlates with the interstitial pyruvate level. The levels of glutamine and pyruvate were associated with the metabolic activity of astrocytes, and increased interstitial concentration of these molecules was associated with increased cerebral perfusion pressure (CPP), low ICP, and good recovery after SAH [299–301].

#### Expression of endothelin 1, heme oxygenase 1 and GFAP is increased in astrocytes following SAH

Astrocytes contribute to brain damage also through increased expression of endothelin 1 (ET-1) following SAH [302]. In vivo and in vitro studies showed that astrocytes might be one of the major sources of ET-1 production [302, 303]. ET-1 activates ET_A_ and ET_B_ receptors which play an important role in the pathophysiology after SAH. Activation of astrocytic ET_B_ receptor leads to astrocyte hypertrophy and decreases EAAT-2 expression resulting in higher glutamate toxicity. Other effects of ET-1 like the increased expression of MMP-2, MMP-9, VEGF also contribute to the BBB alteration.

Nevertheless, there are some potential beneficial effects of ET-1, mainly the ability to produce BDNF, glial cell line-derived neurotrophic factor (GDNF), and neurotrophin-3 (NT3) [248]. Astrocytes secrete BDNF and other trophic factors in response to brain damage. BDNF was upregulated in astrocytes as well as microglia and neural stem cells of the subventricular zone between days 5 and 7 following SAH [304]. As was described above, ET-1 contributes to the development of cerebral vasospasm mainly through the activation of the endothelin A (ET_A_) receptor, which lowers the expression of eNOS expression as well as NO production through the PKC-dependent pathway. It leads to K^+^ channel dysfunction and subsequent hyperpolarization and vasodilatation [303, 305].

Increased expression of glial fibrillary acidic protein (GFAP) and heme oxygenase 1 (HO-1) expression was found in reactive astrocytes after SAH. Upregulation of GFAP is probably due to PDGF released by platelets crossing the endothelium and basal lamina into the brain parenchyma, as was described above [137, 296]. Astrocytes that rapidly upregulate HO-1 and ferritin increase their resistance to heme-mediated injury [306, 307].

Increased ferritin expression in astrocytes is cytoprotective as it attenuates neuronal Hb toxicity. Following SAH, however, haptoglobin-Hb complexes are taken up by CD163 receptors localized on microglia and neurons and thus move the iron away from astrocytes [307, 308].

#### Astrocyte cell death following SAH

Thioredoxin-interacting protein (TXNIP), a natural antagonist of thioredoxin (TRX), may play a role in promoting cell death after SAH. Increased TXNIP expression was found in astrocytes and microglia with a peak 48 h after SAH followed by a decrease 72 h after SAH induction. In addition to apoptosis induction, TXNIP is involved in the production of ROS and contributes to the development of inflammation after SAH [309]. Finding that apoptosis also occurs in astrocytes only corroborates the observation of increased caspase-12 peaking at 3 days after SAH [310]. Moreover, increased astrocytic, as well as neuronal cleaved caspase-3 immunoreactivity, was found in the hippocampus and cortex, but not in the brainstem 7 days following SAH. This suggests that astrocytes undergo apoptosis also at later stages following SAH [311].

Gelsolin (GSN), a protein found in astrocytes, neurons, and microglia, mediates the Ca^2+^-dependent severing, capping, and nucleating of actin filaments, and might be involved in the apoptotic process following SAH. Decreased GSN expression was found 12 h after induction of SAH, suggesting a role in the pathophysiology of EBI after SAH [312].

However, Rollins et al. observed that apoptosis seems to be a minor contributor to astrocytic cell death after OxyHb exposure. On the other hand, OxyHb induced necrosis was observed in a large proportion of cultured astrocytes suggesting that SAH leads predominantly to astrocytic necrosis rather than apoptosis [313]. Necrosis of astrocytes is accelerated by extracellular ATP, whose concentration is many times higher in CSF after SAH compared to normal conditions. ATP activates G-protein-coupled P2Y receptors leading to IP_3_-dependent intracellular Ca^2+^ release from ER. This massive Ca^2+^ release leads to the opening of mitochondrial permeability transition pores and subsequent astrocytic necrosis [314].

#### Transporters and ion changes in astrocytes after SAH

AQP-4 is the main aquaporin expressed in the circumvascular astrocytic endfeet. It facilitates interstitial fluid (ISF) circulation within the glymphatic system [315]. AQP-4 has been proposed to play a role in the development of inflammatory changes after SAH. Astrocytic AQP-4 channels are in contact with blood components and blood degradation products that enter the perivascular spaces after SAH. However, the deletion of *AQP-4* did not alleviate neuroinflammation following SAH [141]. Moreover, higher levels of AQP-4 and AQP-1 expression were found in the astrocytic processes after SAH [316]. This suggests that upregulation of AQP-4 expression contributes to reduced brain edema by elimination of excess water from the brain following SAH [317]. Transport and elimination of ISF along with toxic products following a hemorrhagic stroke from the brain through AQP-4 may thus be important in detoxifying brain tissue and mitigating both brain edema and EBI following SAH [315]. In contrast to this, Cao et al. suggested that AQP-4 expression in astrocytic endfeet may be involved in cerebral edema formation following SAH [318]. Swelling of pericapillary processes is believed to be a key component of cytotoxic brain edema following SAH. The normal route for water and potassium efflux from the neuron is through the perivascular astrocytic endfeet and through molecular channels on the astrocytic membrane, where AQP-4 and the inward-rectifying K^+^ channel 4.1 (Kir4.1) mediate the spatial K^+^ buffering action of astrocytes. Following SAH, K^+^ ions released from neurons are moved into astrocytes via K^+^ channels like Kir2.1. Since the Kir4.1 channel is impaired in astrocytic endfeet after SAH, K^+^ accumulates in astrocytes, and water molecules move passively through the more numerous AQP-4 into the astrocytes. It was found that AQP-4 and Kir4.1 channel expression is dependent on p53 protein activation as well as on the activity of the p38MAPK pathway [319].

Changes in K^+^, Ca^2+^, and glutamate concentrations caused by astrocytic endfeet alterations contribute to the disruption of neurovascular coupling after SAH. Neurovascular coupling is shifted from vasodilation under physiological conditions to vasoconstriction during SAH.

Moreover, it seems that uncoupling between neuronal cells and astrocytes occurs within the first hour of sustaining the injury. Astrocytes express soluble adenylyl cyclase, a HCO_3_^−^ sensor, that is activated by increased extracellular K^+^ after SAH and mediates the entry of HCO_3_^−^ into astrocytes. Increased HCO_3_^−^ levels trigger the cyclic AMP cascade leading to the initiation of glycolysis and formation of lactate and subsequent neuronal injury [320]. However, the Ca^2+^ concentration in the astrocytic endfeet isnot significantly different 4 h after SAH. This suggests that neurovascular coupling is altered by loss of CO_2_ reactivity, dependent on NO signaling rather than through increased Ca^2+^ concentration in astrocytic endfeet in the immediate aftermath of SAH [321, 322]. Increased intracellular Ca^2+^ levels are potentiated by the activation of metabotropic (P2Y) purinergic receptor expression in astrocytes. Extracellular purine nucleotides, like ATP, released after SAH activate endfeet G_q_-coupled P2Y receptors that contribute to endfeet high-amplitude Ca^2+^ signals (eHACSs), a mechanism that results in the inversion of neurovascular coupling [323].

The generation of eHACSs after SAH is likely due to the increased expression of IP_3_, but increased IP_3_ receptor sensitivity could also be behind the generation of eHACSs—SAH induced a high-amplitude Ca^2+^ signal following IP_3_ mediated Ca^2+^ release from the ER. Activation and production of IP_3_ is the result of G_q_-coupled receptor activation after SAH [324, 325].

Increased concentration of cyclooxygenase-1 (COX)-derived PGE_2_ that is released from astrocytes and neurons contributes to the alteration of neurovascular coupling through the EP1 receptor-mediated constriction of cerebral arterioles after SAH. The PGE_2_- driven vasoconstriction of cerebral arteries was observed only at high PGE_2_ concentrations. On the other hand, low amounts of PGE_2_ released under physiological conditions contribute to E-type prostanoid receptor 4 (EP4) receptor-mediated vasodilation. This vasodilatory effect of EP4 is mediated by the stimulatory G protein (Gs) dependent stimulation of adenylyl-cyclase and increased production of the vasoactive and neuroprotective cyclic adenosine monophosphate (cAMP) and subsequent protein kinase A (PKA). But activation of EP1 receptor also leads to an increase of intracellular Ca^2+^ level resulting in vasoconstriction of vascular smooth muscle cells [326–328].

Metabotropic glutamate receptors (mGluRs) are activated in astrocytic processes that are stimulated by neuronal activation associated with glutamate release, and this leads to activation of the IP_3_ cascade and elevated Ca^2+^ level in astrocytic endfeet. The increased amplitude of spontaneous Ca^2+^ oscillations in astrocytic endfeet engenders a K^+^ efflux via endfoot large-conductance Ca^2+^-activated K^+^ (BK) channels. When the threshold of perivascular K^+^ exceeds 20 mM, it induces the depolarization of the smooth muscle membrane potential and parenchymal arteriolar contraction [329]. Increased concentration of K^+^ and glutamate depolarize not only smooth muscle cells but also nearby neurons and cause spreading depolarization in the grey matter because of contiguity [330]. Elevated intracellular Ca^2+^ in astrocytes and this spreading depolarization together result in the release of vasodilators like ATP or NO. On the other hand, spreading depolarization also induces astrocytes to release molecules such as prostaglandins, thromboxane, or other cyclooxygenase products that have strong vasoconstrictive effects. Spreading depolarization after SAH may also increase the activity not just of neuronal but also astrocytic ATPase. This is associated with a prolonged period of elevated O_2_ utilization required for the recovery and reversibility of ion concentrations following spreading depolarization [331, 332].

#### Inflammatory response of astrocytes to SAH

Astrocytes also induce a potent inflammatory response after SAH. Increased expression of TNF-α, IL-1β, IL-6, IL-33, and MMP-9 was found in astrocytes after induction of SAH or stimulation by OxyHb [296, 333]. Increased expression of pro-inflammatory molecules stimulated by OxyHb results in increased activity of NF-κB. Nuclear factor-erythroid 2-related factor 2 (Nrf2) plays an important role in regulating the inflammatory response after SAH. Loss of Nrf2 in astrocytes enhanced the activity of NF-κB, resulting in the aggravation of the inflammatory response and apoptosis of astrocytes and the consequent poor prognosis [334].

Platelet-derived growth factor β subunit (PDGF-BB) levels increased in the CSF after SAH. Activation of astrocytic PDGFRβ leads to its phosphorylation and the activation of downstream pathways such as the mitogen-activated protein kinase (MEK)/ERK, STAT3, and PI3K/Akt pathways, which results in the expression of neurotrophic factors and synaptic recovery in the hippocampus after SAH [335].

Yet, reactive astrocytes are able to induce scar formation and neurocan upregulation. The mechanism of scar formation is probably due to leakage of fibrinogen-bound latent TGF-β interaction with reactive astrocytes after BBB disruption or vascular rupture. This interaction leads to active TGF-β formation and activation of the TGF-β/Smad signaling pathway in astrocytes which induce scar formation and neurocan production [336]. On the other hand, reactive astrocytes, as well as capillary endothelial cells, are also responsible for the protection of the NVU via delayed osteopontin (OPN) upregulation that increases MAPK phosphatase-1 (MKP-1) and decreases vascular endothelial growth factor-A (VEGF-A) levels in the brain after SAH [214]. OPN, as well as Tenascin-C, represent matricellular proteins involved in the pathophysiology of SAH [337].

Moreover, norrin, a small molecule protein secreted by astrocytes, may also play a role in this pathophysiology. Norrin acts through its receptor Frizzled-4, which promotes β-catenin nuclear translocation leading to increased expression of occludin, vascular endothelial cadherin (VE-cadherin), and ZO-1. In this way, astrocytes may affect surrounding endothelial cells as well as BBB [338].

### Response of microglia to SAH

**Fig. 6:**
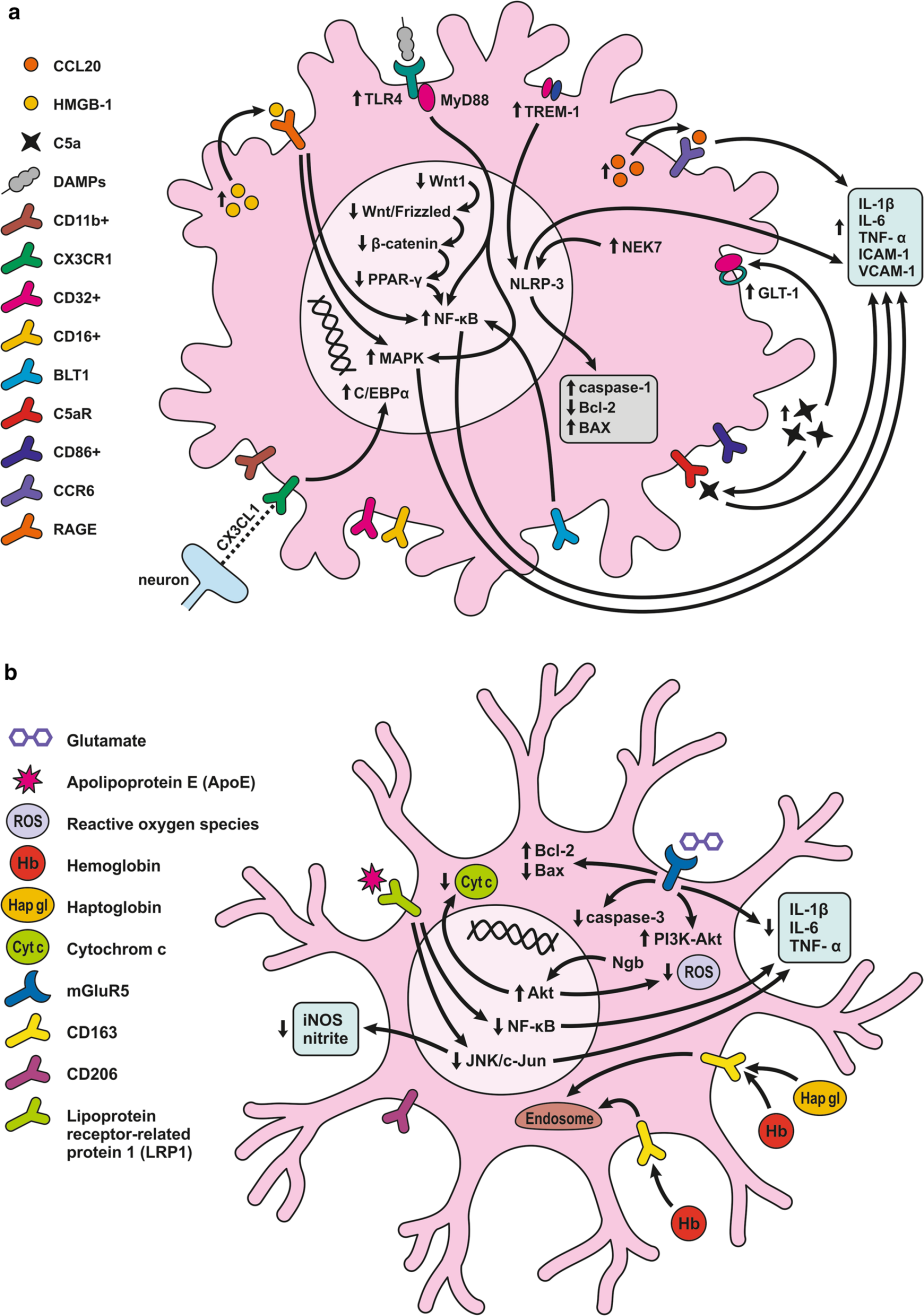
Reaction of microglia to SAH. **a** Activated microglia-induced inflammation. Presence of CD11b^+^/ CD16^+^/ CD32^+^/ CD86^+^ on microglia promotes inflammatory activation of M1 type. TLR4 and BLT1 activation upregulate NF-κB, initiating inflammatory cytokine production and resulting in EC and neuronal apoptosis. Upregulated NEK7 and TREM-1 activate NLRP3, promoting caspase-1 and IL-1β maturation, Bax upregulation, and Bcl-2 reduction. CCR6 also promotes inflammation after the increased release of CCL20. C5a receptor responds to released C5a and also contributes to the increased production of inflammatory cytokines. Downregulation of the CX3CL1/CX3CR1 axis causes an increase in C/EBPα, resulting in pro-inflammatory responses. RAGE is activated via HMGB1, causing MAPK upregulation, thus NF-κB activation, and brain inflammation. Wnt1 downregulation suppresses the Wnt/Frizzled signaling pathway, which leads to β-catenin reduction. Downregulated PPAR-γ then results in inflammatory responses by NF-κB activation. **b** Protective role of microglia after SAH. Interaction of CD163 with Hb results in Hb internalization to endosomes for degradation into heme, peptides, and amino acids. mGluR5 regulates glutamate detoxification and reduces pro-inflammatory cytokines IL-1β, IL-6, and TNF-α. Activation of mGluR5 also leads to Bcl-2 upregulation and the downregulation of Bax and active caspase-3. PI3K-Akt pathway activation and subsequent cell survival are regulated by mGluR5. The Akt signaling pathway is activated by neuroglobin functioning as a ROS scavenger and Cyt c release inhibitor. LRP1 activation by ApoE downregulates the NF-κB inflammatory cascade and inhibits the JNK/c-Jun pathway, suppresses microglial activation, and inhibits iNOS and nitrite accumulation

Microglial cells are key cells mediating neuroprotection, neuroinflammation, and neuronal apoptosis following SAH. The reaction of microglial cells on SAH is diffuse and results in a systemic response in brain tissue (Fig. 6a; Table 3) [296]. Generally speaking, neuroprotection is mediated through the detoxification of neurotoxic blood products or the expression of neuroprotective proteins (Fig. 6b).

**Table 3:**
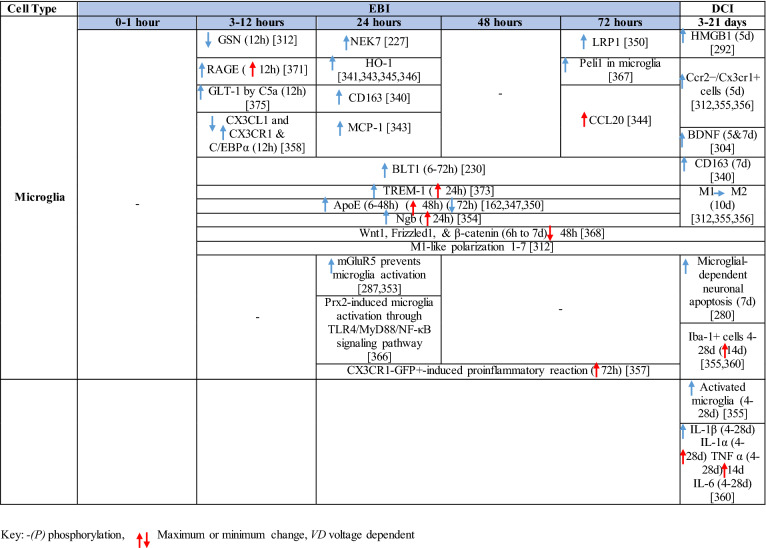
Reaction of microglia to SAH

#### Neuroprotection by microglia following SAH

Following SAH, extracellular Hb binds with high affinity to haptoglobin and haptoglobin/Hb complexes, and in the absence of haptoglobin, Hb can be taken up by microglia via the CD163 receptor with lower affinity. Hb is subsequently internalized through the interaction with CD163 and transferred to endosomes, where it is degraded to heme, peptides, and amino acids [339]. Heme oxygenase-1 (HO-1) plays a significant role in the degradation of pro-oxidant heme, and the Hb/haptoglobin-CD163-HO-1 system contributes to hematoma clearance and defense against Hb neurotoxicity as well as erythrophagocytosis. Overexpression of CD163 positive cells was associated with the severity of SAH. Apart from microglia, CD163 expression was also found in macrophages, neurons, and oligodendrocytes after SAH [340, 341]. Further, HO-1 expression was found in microglial cells throughout the brain, including the thalamus, striatum, hippocampus, cerebral and cerebellar cortex, forebrain white matter, as well as in the choroid plexus following SAH [342].

Microglial HO-1 dependent cytokines may influence neuron survival after hemorrhagic stroke [343]. Expression of HO-1 in microglia was associated with the upregulation of monocyte chemoattractant protein-1 (MCP-1/CCL2), which causes migration and proliferation of microglia without directly activating their inflammatory response [343]. However, CCL20 localized on microglia and neurons promotes inflammation via its cognate CCR6 receptor also expressed on microglia. CCL20/CCR6 induces microglial activation and pro-inflammatory mediator release, thereby increasing neuronal apoptosis [344]. There is some evidence that not only cytokines but also the release of carbon monoxide (CO), one of the products of heme catabolism by HO-1, contributes to the neuroprotective and antiapoptotic effects of HO-1 after SAH [341, 345]. A recent study confirmed the neuroprotective effect and the ability of CO to stimulate microglial phagocytosis of erythrocytes after hemorrhage [346].

One of the protective molecules, ApoE, supposedly has a beneficial effect on the pathological process via BBB preservation after SAH [162]. ApoE at least partially attenuates microglia-induced inflammation by suppressing the JNK/c-Jun signaling cascade [347, 348]. Moreover, inhibition of the JNK/c-Jun pathway suppresses microglial activation and inhibits iNOS and nitrite accumulation [349]. Low-density lipoprotein receptor-related protein-1 (LRP1), an endogenous ApoE receptor as well as ApoE were co-localized with microglia inside the white matter regions and were increased after 72 h following SAH.

LRP1 was expressed mainly in M2 microglia (CD206 + / CD163 + / Arg1 +) but less so in M1 microglia (CD11b + / CD16 + / CD32 + / CD86 +), thereby pointing to the immunosuppressive phenotype of M2 positive microglia [347, 350]. The anti-inflammatory response of LRP1 in microglia is due to the suppression of microglial activation by modulating the JNK/c-Jun and NF-κB signaling pathways [351]. The protective effect of ApoE may be also explained by the suppression of excessive activation of the LRP1/JAK2/STAT3/ NADPH oxidase 2 pro-inflammatory cascade that leads to M1 microglia transformation after SAH [352].

The neuroprotective mGluR5 receptor was expressed in activated ED1 (CD68 +) microglia after SAH. Experimental activation of mGluR5 reduces microglia activation and mRNA levels of the pro-inflammatory cytokines IL-1β, IL-6, and TNF-α after SAH. Activation of mGluR5 leads to neuroprotection by decreasing the number of apoptotic cells, the up-regulation of Bcl-2 expression, and the down-regulation of Bax and active caspase-3 expression [287]. Several molecular mechanisms have been proposed to explain the neuroprotective action of mGluR5, including activation of the PI3K-Akt pathway leading to cell survival, action through the GluA2 subunit of AMPA receptors, or reactive astrogliosis [353].

Another endogenous neuroprotective molecule, neuroglobin (Ngb), is located in microglia as well as in the neuronal cytoplasm. After SAH, Ngb activates the Akt signaling pathway, which functions as a reactive oxygen species (ROS) scavenger, and inhibitor of cytochrome c release from mitochondria, thus protecting against N-Methyl-D-aspartate (NMDA) toxicity and hypoxia re-oxygenation injury [354].

BDNF, an important member of the neurotrophic factor family, was also increased after SAH, its up-regulation of BDNF expression being found in microglia, astrocytes, and neural stem cells in subventricular zones 5 and 7 post-SAH [304].

#### SAH-induced inflammatory reaction of microglia following SAH

It was suggested that resident microglia rather than macrophages are responsible for the initial inflammatory reactions. Early on, from day 1 to day 5 after SAH, microglia underwent M1-like polarization, to they adopted an “activation” morphology with thicker, simpler, less branched processes and a generally swollen and more amoeboid form. Almost all of the Iba1 + microglia expressed gelsolin (GSN) 1 day after SAH. GSN is a protein that mediates Ca^2+^-dependent severing, capping, and nucleation of actin filaments, thus acting as a regulator of cell structure and metabolism and could play a role in changing morphology [312].

In the delayed phase (10 days after SAH), M1 microglia are converted to M2 phenotype characterized by scavenging debris, expression of anti-inflammatory molecules, and promoting angiogenesis. Despite the finding that BBB is altered after SAH, the majority of Iba1-positive cells 5 days following SAH are Ccr2 − /Cx3cr1 + cells, suggesting that resident microglia rather than peripheral immune cells are the cellular mediators of inflammation in the CNS after SAH [312, 355, 356]. Similar results were published by Xu et al., who found that CCR2 macrophages do not enter until at least 48 h following induction of SAH [357]. These results suggest that inflammatory changes are mainly caused by activated microglia rather than recruited peripheral monocytes early on after SAH.

CX3C-chemokine ligand 1 (CX3CL1)/ CX3C-chemokine receptor 1 (CX3CR1) may play an important role in microglial activation and EBI after SAH. CX3CL1 (fractalkine) expressed in neurons, and its receptor CX3CR1 located in microglia form a signaling pathway between neurons and microglia. CX3CL1 and CX3CR1 protein levels were significantly reduced 12 h after SAH, which was accompanied by increased protein CCAAT-enhancer-binding protein α (C/EBPα) expression [358]. C/EBPα is a key regulator of microglia quiescence, and increased levels of this protein were associated with higher numbers of activated CD45 + and MHC II + microglia resulting in a pro-inflammatory response [359]. A robust pro-inflammatory reaction induced by resident CX3CR1- green fluorescent protein (GFP) + microglial cells develop in the cortex as early as 24 h after SAH and reaches a maximum at 72 h [357]. However, it seems that microglial activation continues also later on after SAH [355]. Schneider et al. described that the number of Iba-1 positive (activated) microglia was highest 14 days after hemorrhage. Double immunostaining using Iba-1/GFP showed that most Iba-1 positive cells lacked GFP immunofluorescence, suggesting that the Iba‐1‐positive cells originate from the pool of resident microglia instead of peripherally derived myeloid cells [360]. Activated microglia begin to accumulate 4 days after SAH and decline gradually by day 28. However, there is some evidence that microglia may play a role in brain damage several months or even years after SAH [355].

Increased numbers of activated microglia occur following neutrophil-endothelial interaction induced by intercellular adhesion molecule 1 (ICAM-1) and P-selectin glycoprotein ligand-1 (PSGL-1) during the first 4 days after SAH. Intravascular inflammation and subsequent microglial activation were described as “cerebral spreading inflammation,” reflecting neuronal cell death after SAH [228, 360]. However, it remains questionable whether increased microglial activation following increased neutrophil infiltration is independent events mediated by parenchymal changes or whether microglia activation is somehow related to neutrophil recruitment [361].

Controversially, Gris et al. found increased numbers of activated resident microglia but also recruited monocytes 1 and 2 days after induction of SAH. This observation points to early intracerebral peripheral monocyte infiltration and innate immune activation [362].

Different signaling pathways downstream of TLR4 play a significant role in inflammatory neuronal injury. Various TLR4 ligands, including heme, methemoglobin, hemin, and OxyHb, are released from lysed erythrocytes and act as potent DAMPs [280, 281, 363]. Activation of microglial TLR4 by DAMPs was recently shown to cause SAH-induced pyrexia [364]. In the early phase of SAH (7 days after induction of SAH), neuronal apoptosis is largely TLR4-MyD88-dependent and microglial-dependent. On the other hand, in the later phase (15 days following induction of SAH), neuronal apoptosis was seen to be TLR4/ TLR4–associated activator of interferon (TRIF) dependent and microglia-independent [280]. Akamatsu et al. suggested that heme released to CSF after SAH acts as a potent DAMP and activates the microglial MyD88 cascade in the early phase while also activating the TRIF pathway in the later phase. The MyD88, as well as the TRIF cascades, lead to the expression of NF‐κB and MAPK, resulting in apoptosis, increased expression of pro‐inflammatory genes and adhesion molecules [365]. Another TLR4 ligand, peroxiredoxin 2 (Prx2), which is abundant in both erythrocytes and neurons, is considered a DAMP when it is released to the extracellular space. Prx2 was found to be the second most elevated protein in the CSF following SAH. Prx2 activates microglia to the M1 phenotype through the TLR4/MyD88/NF-κB signaling pathway. It promotes the synthesis and secretion of IL-1β and IL-6 from microglia, which leads to neuroinflammation and neuronal apoptosis [366]. Activation of the MyD88-dependent signaling pathway is facilitated by pellino homolog 1 (Peli1), which increases in microglia during the first 72 h after SAH. Peli1 acts by activating a cellular inhibitor of apoptosis proteins 1/2 (cIAP1/2). Up-regulation of cIAP1/2 facilitates phosphorylation of the MAPK pathway and promotes microglia polarization to the M1 phenotype, releasing pro-inflammatory cytokines [367].

These pro-inflammatory cytokines increase the expression or activity of proteolytic enzymes, which can alter vascular endothelial cadherin (VE-cadherin) and generate VE-cadherin fragments. These fragments interact with the MyD88/NFκB pathway and shift microglia towards a more pro-inflammatory state characterized by increased microglial cell size of Iba1 immuno-positive cells. Schneider et al. found microglia as the sole source of IL-6- as well as TNF-α in the later phase of SAH but the proportion of microglia expressing IL-6 was much lower compared with that expressing TNF-α (approximately 30%). Moreover, the corresponding cytokine receptors were also up-regulated, suggesting a paracrine/autocrine action of these cytokines [360].

Expression of NF-κB as a major regulator of inflammation, expression of genes for inflammatory cytokines, enzymes, and adhesion molecules may be affected by peroxisome proliferator-activated receptor gamma (PPAR-γ). Following SAH, expression and secretion of Wnt1 protein are decreased, which is accompanied by the suppression of the Wnt/Frizzled signaling pathway leading to reduction of β-catenin. Downregulation of β-catenin leads to decreased intranuclear PPAR-γ expression and results in ineffective antagonism of NF-κB [368]. Similarly, a recent paper revealed that soluble VE-cadherin fragments in the CSF might interact with the MyD88/NFκB pathway and shift microglia towards a more pro-inflammatory state [369].

Leukotriene B4 (LTB4) receptor 1 (BLT1) stimulates NF-κB-dependent inflammation and may promote the inflammatory response after SAH. BLT1 was mainly expressed in microglia, neurons, and endothelial cells. Its increased expression was found as early as 6 h and lasted up to 72 h after SAH [230]. Increased expression of HMGB1 may contribute to the maintenance of inflammation in the later stage. Cytosolic levels of HMGB1 were upregulated in activated microglia 5 days after induction of SAH. HMGB1 activates MAP kinase pathways through receptors for advanced glycation end products (RAGE); it also up-regulates the transcription factor NF-κB and promotes brain inflammation [292].

During the acute phase of SAH, a small number of microglia was found to release HMGB1 into the extracellular space. This suggests that microglial HMGB1 may be responsible for inflammation in the later stage following SAH [370]. Accumulation of RAGE was increased in microglia after SAH. However, in contrast to HMGB1, increased levels of RAGE were found in the early stage of SAH and reached their peak at 12 h after SAH [371].

Microglial cells are the main source of leucine-rich repeat (LRR)-containing protein 3 (NLRP3) inflammasome belonging to the NLR family—a group of innate immune proteins considered to be sensors of PAMPs and DAMPs. They are able to promote caspase-1 and IL-1β maturation and secretion as well as increase the level of pro-apoptotic protein Bax and decrease the expression of anti-apoptotic Bcl-2 protein. The serine/threonine protein kinase 7 (NEK7) is critical for NLRP3 activation; increased NEK7 expression was found in microglia and peaked 24 h after SAH [227].

Nonetheless, there are other activators of NLRP3, such as extracellular ATP, K^+^ ionophores, crystals, insoluble particles, certain pathogens, ROS, K^+^ efflux, and endolysosomal leakage [372]. Most of these activators are present in the brain after SAH. Therefore, NLRP3 inflammasome activation may play a key role in the development of inflammation and increased barrier permeability after SAH. Triggering receptor expressed on myeloid cells 1 (TREM-1), a transmembrane protein on microglial cells, is also involved in NLRP3 inflammasome activation after SAH. Increased expression of TREM-1 was found over 72 h, with a peak at 24 h after SAH [373].

The complement system also plays an important role in the innate inflammatory response after SAH. Activation of the complement system results in the cleavage of complement component 5 (C5), yielding C5a and the lytic membrane complex C5b-C9. Increased levels of C5a were found in the CSF over the first 5 days after SAH. C5aR, the receptor for this complement-activated product, is also expressed on microglia and contributes to the development of neuroinflammation and cerebral vasospasm [374]. Evidence shows that C5a increases microglial GLT-1 expression and removes extracellular glutamate, which plays an important role in BBB disruption and causes excitotoxicity following a stroke [375–377].

### Reaction of neurons to SAH

#### Neuronal protective mechanisms after SAH

**Fig. 7:**
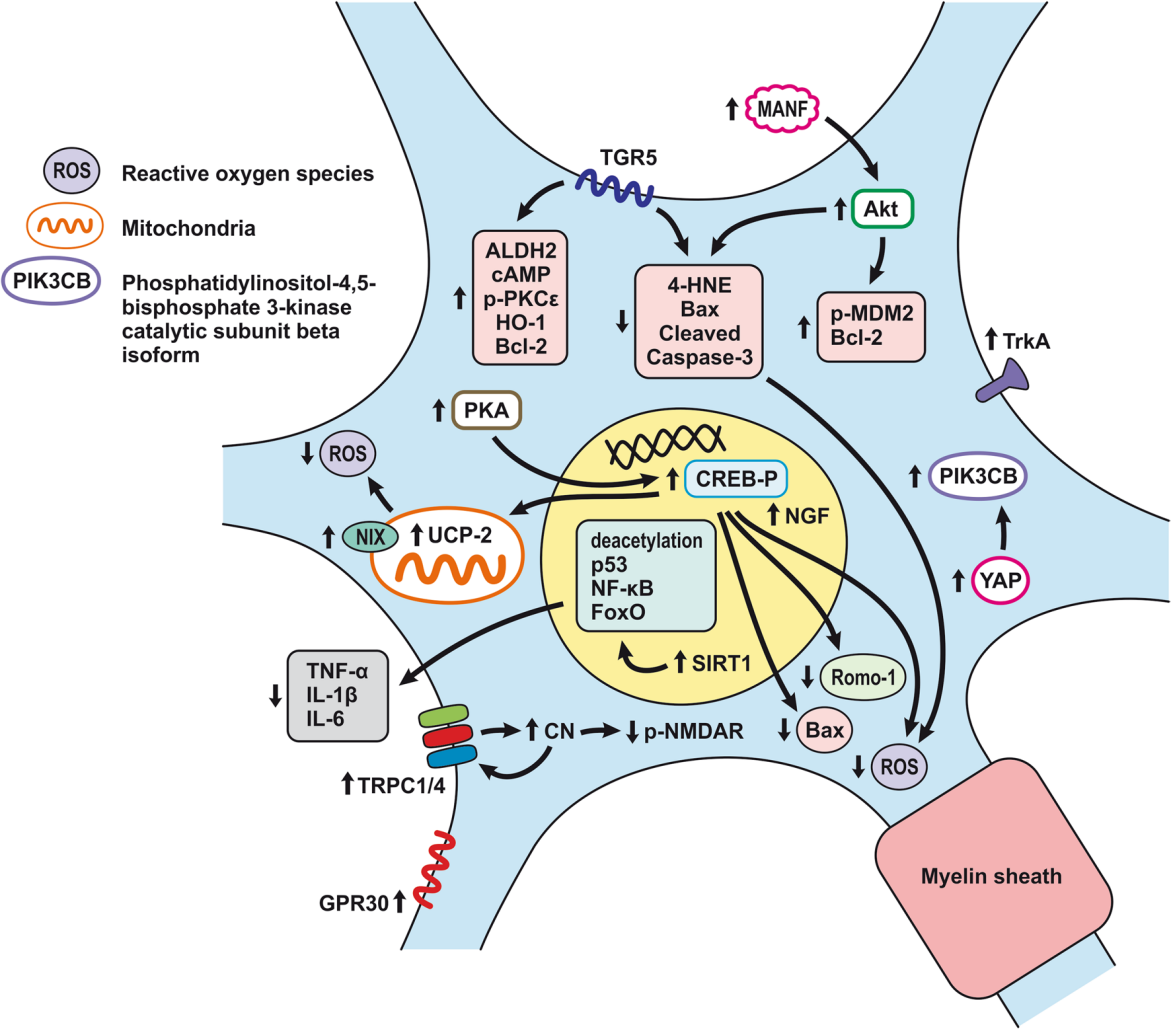
Neuronal protective mechanisms after SAH. SAH-induced neuroprotection involves decreased cell apoptosis, necrosis, neuronal regeneration, oxidative injury, inflammation, and removal of damaged mitochondria. Attenuation of neuronal apoptosis is supported by molecules up-regulated by TGR5, MANF, Akt, SIRT1, UCP-2, and GPR30. Increased expression of TGR5 leads to up-regulation of anti-apoptotic protein levels including cAMP, p-PKCε, ALDH2, HO-1, Bcl-2, and down-regulation of pro-apoptotic proteins such as 4-HNE, Bax, and cleaved caspase-3. SAH induced. MANF leads to activation of Akt, resulting in up-regulation of p-MDM2 and Bcl-2 and down-regulation of pro-apoptotic molecules. Up-regulation of SIRT1 contributes to the deacetylation of acetylated-forkhead box O, acetylated-NF-κB, and acetylated-p53, thus attenuating the oxidative, inflammatory, and apoptotic pathways. Increased UCP2 levels lead to decreased ROS generation. Moreover, an increase in PKA expression phosphorylates CREB, which results in UCP2 upregulation and downregulates Bax and Romo-1. Up-regulation of TrkA, NGF, and YAP/PIK3CB contribute to neuronal growth and regeneration and BBB maintenance (increased expression of occludin and claudin-5 induced by YAP/ PIK3CB pathway). Dephosphorylation of NMDAR via up-regulation of TRPC1/4 and subsequent CN protects neurons from excitotoxicity

One of the main causes of cognitive deficit after SAH is the decreased number of neuronal cells [202, 378–380]. On the other hand, SAH also triggers some healing cascades by which the brain is protected against the consequences of bleeding from a ruptured aneurysm (Fig. 7; Table 4). However, protective and harmful mechanisms can occur simultaneously, given the interaction between neurons, microglia, and astrocytes. This statement is supported by the observation that TNF-α activates astrocytes to a deleterious A1 type, whereas expression of prokineticin 2 (PK2) in neurons increases and subsequently promotes the differentiation of astrocytes into a protective A2 type by activating STAT3 [286, 288].

**Table 4:**
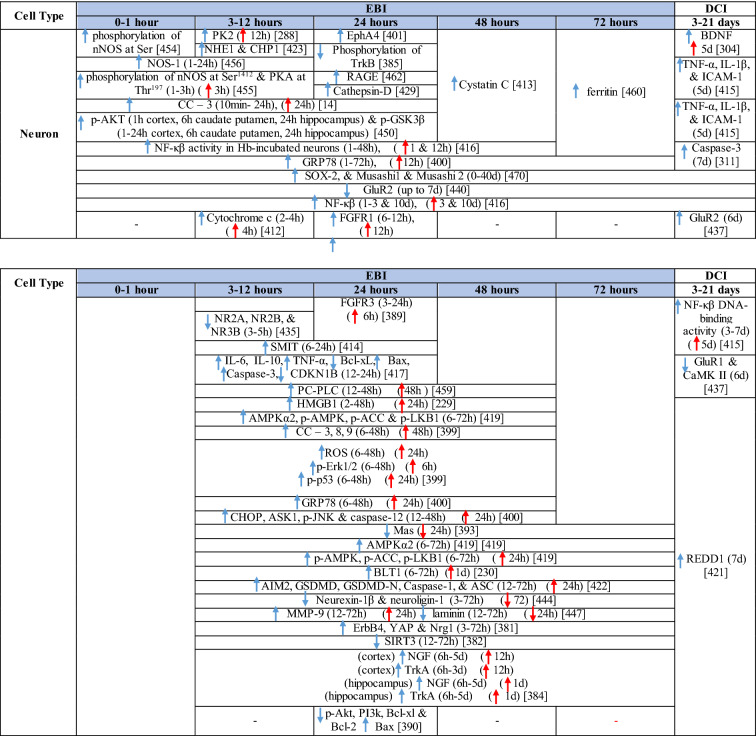

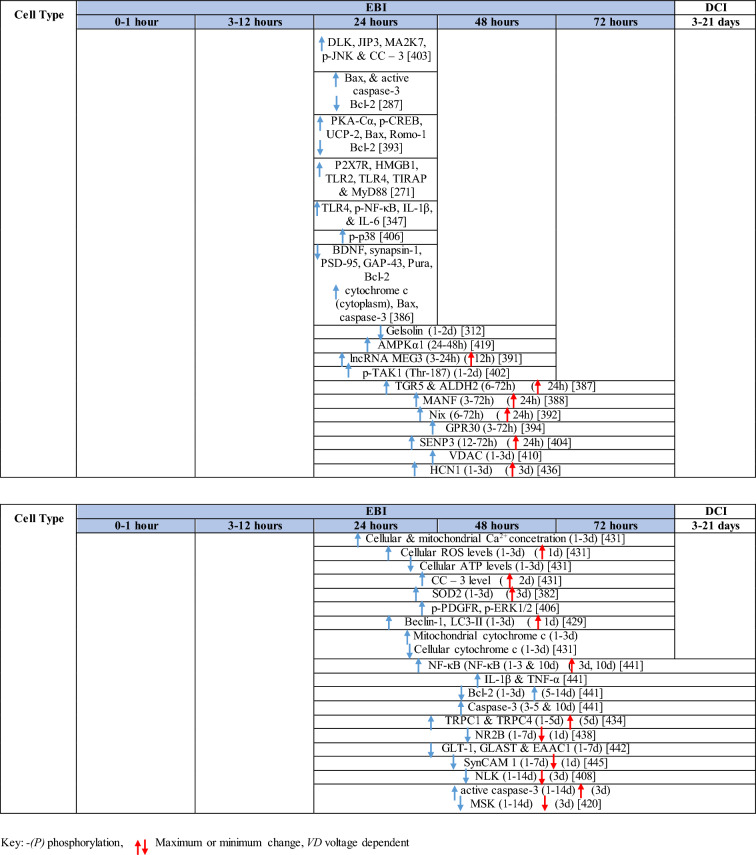
Reaction of neurons to SAH

Following SAH, blood degradation products in the paravascular space can stimulate endothelial cells and pericytes to secrete platelet-derived growth factor β subunit (PDGF-BB), which results in astrocytic activation by stimulation of PDGF-Rβ. The PDGF-BB/ PDGF-Rβ pathway leads to the expression of neurotrophic factors that mediate synaptic recovery after SAH [335]. Increased neuronal survival is also associated with up-regulation of the EGFR family member v-erb-b2 avian erythroblastic leukemia viral oncogene homolog 4 (ErbB4) and yes-associated protein (YAP and both are highly expressed in neurons 72 h after SAH. YAP promotes cellular growth probably through the phosphatidylinositol-4,5-bisphosphate 3-kinase catalytic subunit beta (PIK3CB), a catalytic subunit of phosphoinositol-3-kinase [381]. On the other hand, in neurons, the expression of some proteins such as sirtuin 3 (SIRT3), a member of the family of highly conservative NAD-dependent enzymes—is decreased after SAH. Downregulation of SIRT3 is associated with increased ROS generation as well as cellular apoptosis [382]. However, SAH causes an increased expression of another sirtuin, SIRT1, that is also found predominantly in neurons. SIRT1 expression peaks 24 h after SAH and remains elevated up to 72 h after SAH. Expression of SIRT1 in neurons leads to the deacetylation of acetylated-forkhead box O (ac-FoxO), acetylated-NF-κB, and acetylated-p53, resulting in the attenuation of oxidative, inflammatory, and apoptotic pathways and consequently EBI development [383]. Dynamic changes of nerve growth factor (NGF) expression also contribute to brain recovery following SAH. The highest expression of NGF occurs in the cortex and brainstem 12 h after SAH and after 24 h in the hippocampus. NGF expression dynamics are similar to its functional receptor tropomyosin receptor kinase A (TrkA), with a recovery at 5 days after SAH. These findings suggest that the NGF-TrkA interaction contributes to neuronal protection, regeneration, and axonal growth that are altered mainly in the acute phase of SAH [384]. However, phosphorylation and activation of tropomyosin receptor kinase B (TrkB), a BDNF receptor, is reduced during the first 24 h after SAH resulting in increased apoptosis of neuronal cells [385]. On the other hand, increased BDNF expression is seen in the subventricular zone (SVZ) 5 and 7 days after SAH, indicating proliferation, differentiation, and migration of neural stem cells in the later phase of SAH [304]. In addition to BDNF, cerebral expression of synaptic proteins such as synapsin-1, postsynaptic density protein-95 (PSD-95), and growth-associated protein 43 (GAP-43) or neuronal differentiation factors like purine-rich binding protein-alpha were decreased following SAH [386].

Expression of other endogenous proteins such as trans-membrane G protein-coupled receptor-5 (TGR5) and mitochondrial aldehyde dehydrogenase 2 (ALDH2) gradually increases, peaking at 24 h after SAH. TGR5 leads to upregulation of protein levels, including cAMP, p-PKCε, ALDH2, HO-1, Bcl-2, and the downregulation of 4-Hydroxynonenal (4-HNE), Bax, and cleaved caspase-3. ALDH2 contributes to this protective effect by decreasing ROS accumulation, inhibition of mitochondrial apoptosis, and reversing mitochondrial membrane depolarization resulting in the attenuation of neuronal apoptosis after SAH [387]. Increased expression of mesencephalic astrocyte-derived neurotrophic factor (MANF) also contributes to the reduction in the number of apoptotic neuronal cells after SAH. MANF exerts its anti-apoptotic effect through the Akt- dependent pro-survival pathway mainly at 3 h and peaking 24 h after SAH. Activation of Akt leads to up-regulation of p–mouse double minute 2 homolog (p-MDM2) and Bcl-2, and the down-regulation of P53, Bax, cleaved caspase-3, and MMP-9 [388]. However, Okada et al. found that expression of PI3k and p-Akt is not elevated 24 h after SAH. Activation of the PI3k/Akt pathway is partially through the fibroblast growth factor-2 (FGF-2), which binds to the fibroblast growth factor receptors FGFR-1, -2, and -3. However, endogenous FGF-2 may not be high enough to activate the Akt cascade 24 h after SAH [389]. Moreover, there is evidence showing significantly decreased expression of p-Akt as well as PI3k, Bcl-xl, and Bcl-2, along with increased Bax expression following SAH [390]. Overexpression of the long non-coding RNA (lncRNA) maternally expressed 3 (MEG3) contributes to the inhibition of the PI3k/Akt pathway resulting in decreased Bcl-2 expression and increased expression of the pro-apoptotic Bax protein up to 72 h after SAH [391].

Regulation of quantitative and qualitative control of mitochondria is important to maintain neuronal cell stability. Nix, a B-cell lymphoma 2 -interacting protein 3 like protein (Bnip3L), plays a role in the removal of injured mitochondria and amelioration of brain injury. Expression of Nix peaked 24 h after SAH and then gradually decreased. This finding supports the assumption that mitochondrial damage occurs mainly during the first day after SAH onset [392]. The cyclic adenosine monophosphate (cAMP) response element-binding protein (CREB) plays an important role in memory function, synaptic plasticity, regeneration, and cell survival under various stress conditions, including those after SAH. CREB can be phosphorylated by protein kinase A (PKA), which is accompanied by an increase in uncoupling protein 2 (UCP2) level leading to decreased production of ROS and proapoptotic proteins such as Bax and reactive oxygen species modulator (Romo)-1 [393]. Interestingly, SAH induces the expression of G protein-coupled receptor 30 (GPR30), a membrane estrogen receptor, mainly in neuronal cells of male rats at 3 h, with a maximum at 24 h and declining by 72 h after bleeding. Activation of GPR30 attenuates apoptosis of neuronal cells through the src/EGFR/stat3 signaling pathway [394].

#### Neuronal injury and inflammation after SAH

**Fig. 8:**
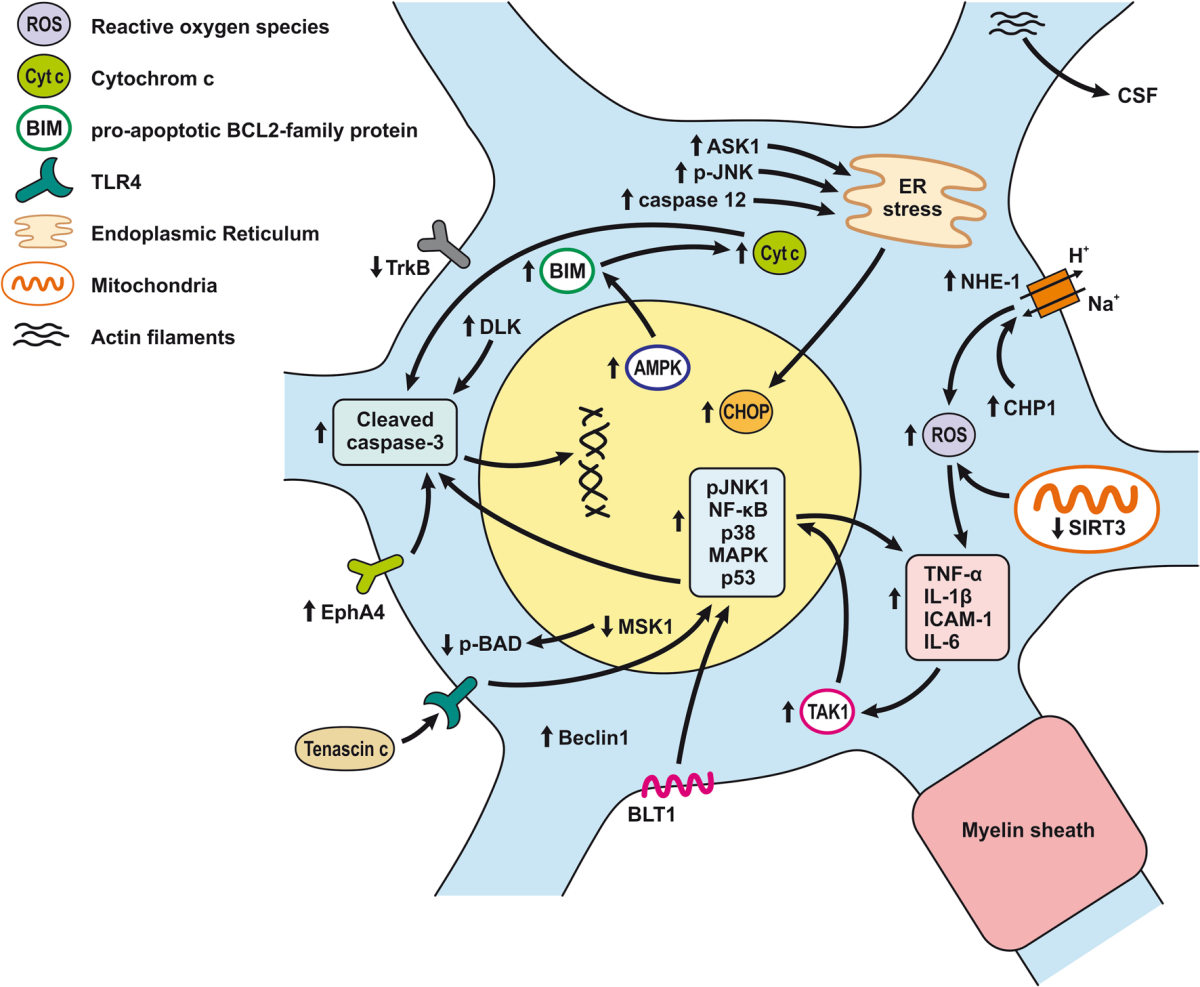
Neuronal injury and inflammation after SAH. Schematic illustration of pathophysiological cascades leading to the development of inflammation and neuronal injury after SAH. Induction of NF-κB via activation of TLR4 (tenascin C), TAK1 (TNF-α and IL-1β), and BLT1 (LTB4) leads to overexpression of proinflammatory molecules such as TNF-α and IL-1β as well as ICAM-1. In addition to pro-inflammatory molecules, NF-κB, JNK, and p38 activate the mitochondrial apoptotic pathway and upregulate cleaved-caspase3 resulting in DNA fragmentation and neuronal death. Prolonged activation of AMPK leads to induction of Bim, which promotes Cyt c release from mitochondria and subsequent maturation of caspase-3. Increased expression of Eph receptor A4 (EphA4) and DLK also contributes to caspase-dependent neuronal death. Down-regulation MSK1 leads to decreased phosphorylation of Bcl2-associated agonist of cell death (Bad) and thus promotes neuronal death. Decreased activation and phosphorylation of TrkB reduces neuronal protection and thus increases neuronal apoptosis. Similarly, increased expression of CHP1 activates Na^+^H^+^-exchanger 1 (NHE1), which contributes to oxidative stress resulting in neuronal death. Down-regulation of SIRT3 contributes to the increased generation of ROS. The endoplasmic reticulum stress-related apoptotic proteins such as C/EBP homologous protein (CHOP), caspase-12, ASK1, and p-JNK are increased following SAH. The induction of these proteins contributes to neuronal apoptosis during the SAH

Diffuse and irreversible neuronal damage develops within several hours after SAH in the cerebral cortex, and they correlate with the severity of SAH (Fig. 8; Table 4) [395].

Despite the protective mechanisms of neuronal cells, SAH leads to morphological abnormalities in cortical and hippocampal neurons, including cytoskeletal and nuclear changes [396]. Increased expression of cleaved caspase-3 has been observed after SAH [362, 397, 398]—with caspase-3-mediated DNA fragmentation being found as early as 10 min after SAH [220]. However, a significant increase of neuronal cleaved caspase-3 was also found 7 days following induction of SAH, indicating neuronal apoptosis also in the later phase [311]. The levels of cleaved caspase-8, caspase-9, and caspase-3 gradually increased in the hippocampus over a time-course of 6, 12, 24, and 48 h following SAH [399]. The expression of glucose-regulated protein 78 (GRP78) supports the observation that apoptosis is highest in the first days after SAH [400].

Caspase-dependent neuronal death was associated with the upregulation of Eph receptor A4 (EphA4) after SAH. EphA4 is also able to induce neuronal death through Ephexin-1, Ras homolog family member A (RhoA), and the ROCK2 signaling pathway, all of which are increased 24 h after SAH [401]. Pro-inflammatory molecules, including TNF-α and IL-1β, could increase TGFβ-activated kinase 1 (TAK1) expression and contribute to neuronal apoptosis 24 h after SAH. Increased TAK1 activity in neuronal cells led to the activation of NF-κB, JNK, and p38, resulting in cleaved caspase-3 expression and neuronal death [402]. Upregulation of neuronal cleaved caspase-3 expression is also affected by dual leucine zipper kinase (DLK), which is increased 24 h after SAH. DLK contributes to neuron apoptosis through the downstream JIP3/MA2K7/JNK pathway resulting in increased cleaved caspase-3 [403].

Cleaved caspase-3 expression in neurons was positively correlated with the amount of small ubiquitin-like modifier-specific protease 3 (SENP3) during the first few days after SAH. This finding suggests a role for SENP3 in inducing apoptosis following SAH [404]. It was found that the MAPK signaling pathway is responsible for the upregulation of caspase activity and neuronal apoptosis [405]. SAH activates PDGF that upregulates tenascin-C (TNC) and subsequently activates MAPKs. The terminal MAPKs, extracellular signal-regulated kinase 1/2 (ERK1/2), and p38 are activated at 24 h, and ERK1/2 is activated at 72 h following SAH [406]. TNC plays also a role as an endogenous TLR4 activator. Upregulation of TNC following SAH contributes to the activation of TLR4/NF-κB/ IL-1β and IL-6 pathway leading to caspase-3 activation and neuronal apoptosis [407]. Nemo-like kinase (NLK) was downregulated 3 days after SAH with a gradual increase over 14 days. Decreased expression of NLK was associated with a peak time-point for cell apoptosis, indicating a role for this kinase in the caspase-3 activation pathway [408].

It has been suggested that neuronal apoptosis is the major contributor of morbidity and mortality after SAH. Neuronal cell loss probably continues into the later phase, and the majority of cells that die after SAH are neurons [380]. At the same time as apoptosis, necrosis and autophagy may occur in neurons. Extensive crosstalk has been described between the pathways for autophagy and apoptosis [409]. Mitophagy, the selective removal of mitochondria by autophagy, is induced by mitochondria itself. Induction of pro-autophagic ROS leads to the formation and elongation of phagophores (LC3) and avoiding autophagy. However, increased expression after SAH of voltage-dependent anion channels (VDAC) interacts with LC3 on altered mitochondria and induces mitophagy [410].

Moreover, it seems that apoptosis via the mitochondrial pathway (associated with reduced cytochrome c release) plays a protective role in EBI after SAH [411]. It was found that cytochrome c is present in the cytosol of neuronal cells 3 h after hemolysate application into the subarachnoid space. Cytochrome c in the neuronal cytosol leads to DNA fragmentation resulting in neuronal death after SAH [412]. Caspase-independent, as well as mitochondrial pathways, play a major role in the pathophysiology of apoptotic cascades, mainly during the first few days following SAH [378]. However, Açıkgöz et al. observed lower neuronal loss during the acute phase of SAH than in the later phase (7 days after induction of SAH). This finding can be explained by Cystatin C, a cysteine protease inhibitor, which was increased 2 days after induction of SAH [413]. Brain structures that are in direct contact with the blood clot can also be altered after SAH. Diffusion of blood degradation products into surrounding brain tissue may cause local hyperosmolarity. Areas like the hypothalamus, the amygdala, and the temporal cortex are directly affected by blood-borne materials. In these areas, expression of Na^+^/myo-inositol cotransporter (SMIT) reflecting osmotic stress is upregulated between 6 to 24 h after SAH [414].

The development of neuroinflammation after SAH is associated with the loss of neurons. The pro-inflammatory changes in neurons mainly involve the activation of NF-κB. NF-κB activity peaked on days 3 and 5 and remained increased 7 days after SAH. Activation of NF-κB contributes to EBI and DCI through the increased expression of pro-inflammatory molecules such as TNF- α, IL-1β, and intercellular adhesion molecule 1 (ICAM-1), mainly in neurons after SAH [415]. You et al. found biphasic NF-κB activity peaks 1, 3, and 10 days after SAH. The early peak in NF-κB activity was associated with decreased number of neurons and increased lactate dehydrogenase released from damaged neurons. On the other hand, the late peak did not aggravate neuronal damage and even might be beneficial for neuronal survival [416]. Activation of the NF-κB inflammatory pathway might be potentiated by BLT1. Expression of both BLT1 mRNA and BLT1 protein was increased; they both peaked on day 1 and remained increased on day 3 after SAH. Moreover, the leukotriene B4 (LTB4)-BLT1 axis might be involved in neutrophil recruitment [230].

PPARγ/NF-κB signaling pathway could be involved in neuronal cells death after SAH. Overexpression of cyclin-dependent kinase inhibitor 1B (CDKN1B) could suppress apoptosis and inflammation. The beneficial effect of CDKN1B manifests through the suppression of NF-κB/p65 and enhanced expression of PPARγ [417].

Gelsolin plays a role in the apoptotic process after SAH. The expression of gelsolin was found mainly in the neurons, microglia, and astrocytes in the brain cortex. The decreased expression of gelsolin reached a minimum of 12 h after SAH and is probably caused by apoptosis-induced gelsolin cleavage [312]. Neurofilament light chain, the significant component of the axonal cytoskeleton, was detected in the CSF 24 h after SAH. The amount of neurofilament light chain in the CSF may reflect cerebral ischemia and disruption of axonal membrane integrity, leading to the release of neurofilament proteins [418]. The caspase-dependent apoptosis of neuronal cells can be promoted by prolonged AMP-activated protein kinase (AMPK). Its activation was associated with the induction of the BH3-only protein Bim (Bcl-2 Interacting Mediator of cell death), which can promote cytochrome C release from mitochondria. Numerous p-AMPK positive neuronal cells were found in the cortex 24 h after SAH [419].

MSK1 (mitogen- and stress-activated protein kinase 1) was decreased, reaching a minimum at day 3 after SAH. MSK1 expression in neurons as well as astrocytes enhances phosphorylation of Bcl2-associated agonist of cell death (Bad) and promotes cell survival [420]. REDD1 (Regulated in development and DNA damage responses 1) may play an essential role through apoptosis and ROS induction in neuronal damage after SAH. Primary cortical neurons treated with blood hemolysate showed a dose-dependent increase in REDD1 expression. Elevated expression of REDD1 in neurons was correlated with increased levels of REDD1 in CSF from patients after SAH [421].

Pyroptotic neuronal cell death, a form of cell death associated with proinflammatory signals, was recently described after SAH. Up-regulation of absent in melanoma 2 (AIM2), a protein that mediates pyroptosis, was found in cortical neurons exposed to OxyHb. Pyroptosis could occur through the AIM2/caspase-1/gasdermin D (inducer of pyroptosis) pathway, mainly during the first 3 days after SAH [422].

Ion homeostasis is altered and contributes to neuronal death after SAH. The level of Na^+^H^+^-exchanger 1 (NHE-1), which plays an important role in maintaining intracellular pH homeostasis, gradually increased and peaked 24 h after SAH. The activity of NHE-1 is regulated through interaction with a calcineurin-like EF hand protein 1 (CHP1) that is increased to a peak at 24 h after SAH. Up-regulation of NHE-1 probably via CHP1 interaction contributes to the development of brain edema, oxidative stress, inflammatory response, neuronal cell death, and cognitive dysfunction [423].

The endoplasmic reticulum (ER) plays an important role in cortical neuronal apoptosis during the first 48 h after SAH. Upregulation of endoplasmic reticulum (ER) stress-related apoptotic proteins like C/EBP homologous protein (CHOP), caspase-12, apoptosis signal-regulating kinase 1 (ASK1), and p-JNK peaked at 24 h and decreased at 48 h after the SAH [424]. Increased expression of phosphorylated JNK1, p38, NF-κB, and p53 promotes the activation of the mitochondrial apoptotic pathway and pro-inflammatory cellular signaling, thus contributing to EBI after SAH [425].

Moreover, direct oxidative damage of RNA leads to wrongly folded or truncated proteins that cause endoplasmic reticulum (ER) stress and activation of the unfolded protein response, resulting in neuronal dysfunction and death after SAH [426]. Despite the induction of pro-apoptotic proteins, ER stress predominantly acts as a pro-survival pathway [409]. Alterations of other subcellular organelles, including mitochondrial dysfunction, the autophagy-lysosomal system, and transcription factors (e.g., Nrf2, NF-κB, and HIF-1), are also involved in the pathophysiology of neuronal injury after SAH [427, 428]. Neurons in deep cortical layers of the fronto-basal cortex displayed numerous autophagosomes and autolysosomes following SAH. The autophagic activity destroys cellular components and may lead to altered cell function. Increased expression of Beclin-1, a Bcl-2 interacting protein required for autophagy, peaked 1 day after SAH and remained elevated for 3 days after SAH. Numerous apoptotic cells were found in the superficial cortical layers in contrast to deep cortical layers. Similarly, cathepsin-D, a hydrolytic enzyme, increased immediately after SAH and peaked 24 h after SAH with a subsequent reduction [429].

#### Glutamate induced neurotoxicity after SAH

Neuronal mitochondria that are disrupted subsequently suppress the formation of N-acetylaspartate (NAA), mainly in patients with impaired perfusion or infarction. Interestingly, in patients without impaired perfusion or infarction after SAH, levels of glutamate and glutamine produced in the mitochondrial matrix were significantly decreased. Therefore, this might be a consequence of impaired energy metabolism in neurons [430]. An essential event for the initiation of neuronal death is cytosolic and mitochondrial Ca^2+^ overload caused by SAH [431]. Accumulation of Ca^2+^ is the consequence of excessive glutamate-mediated excitotoxicity, which activates extra-synaptic GluN1/GluN2B containing N-Methyl-D-aspartate receptors (NMDARs), leading to Ca^2+^ influx. Activation of inositol trisphosphate (IP_3_) through mGluR1 releases Ca^2+^ from the ER and also increases the intracellular level of the Ca^2+^ [432]. Sur1-Trpm4 channels that have a protective effect against excessive intracellular calcium spiking are upregulated in neurons, astrocytes, oligodendrocytes, and microvascular endothelial cells following SAH. These channels mediate depolarization, but if unchecked, the ion flow through them might end in cytotoxic edema and necrotic cell death [433]. Na^+^H^+^-exchangers (NHE1) increased in neurons with a peak at 24 h after SAH and can also contribute to increased intracellular level of Ca^2+^. Excessive activation of NHE1 may lead to intracellular Na^+^ overload, which subsequently causes Ca^2+^ entry via Na/Ca exchanger (NCX), resulting in excessive cytosolic Ca^2+^ accumulation. However, the upregulated interaction with calcineurin-like EF hand protein 1 (CHP1) may also play a role in neuronal death related to NHE1 [423]. The intracellular accumulation of Ca^2+^ leads to the activation of pro-oxidative pathways, including phospholipases, xanthine oxidase as well as nitric oxide synthase. These changes are responsible for pathological changes such as lipid peroxidation as well as protein and DNA oxidation that contribute to neuronal cell death [171].

The N-Methyl-D-aspartate receptors (NMDARs) play an important role in EBI pathophysiology after SAH. One of the self-defense mechanisms targeted at NMDAR is the expression of transient receptor potential channels 1 and 4 (TRPC1/4), members of the voltage-sensitive calcium ion channel family. Increased TRPC1/4 expression following SAH up-regulates the activity of calcineurin (CN) which promotes NMDAR dephosphorylation and protects neurons from excitotoxicity. Moreover, activation of CN promotes nuclear transfer of activated T cells nuclear factor resulting in increased TRPC1/4 expression [434].

The neuroprotective mechanism against decreased cerebral blood flow induced in the acute phase of SAH was apparent in the dentate gyrus but not in the CA1 hippocampal region. This neuroprotective mechanism acts by altering the expression of N-Methyl-D-aspartate receptor subunit 2A (NR2A), NR2B, and NR3B, which contain the binding site for glutamate—thereby reducing NMDAR function and subsequent neuronal death 3 and 5 h after SAH [435]. However, Hb inhibits hyperpolarization-activated/cyclic nucleotide-gated (HCN) channels on CA1 pyramidal neurons and induces hyperexcitability. Inhibition of HCN channels is probably caused by Hb released from the blood clot and the silencing of NO signaling [436].

The number of synapses in the hippocampal CA1 area was lower after SAH, and this decrease has been associated with the loss of long-term potential (LTP) responsible for synaptic plasticity, memory, and learning. Several proteins involved in LTP were reduced following SAH, including Ca^2+^/calmodulin- dependent protein kinase II (CamK II), myelin basic protein (MBP), along with a marked trend towards reduced GluR1 and increased glutamate receptor type 2 (GluR2) [437]. NMDAR may play an important role in the pathogenesis of cognitive dysfunction. Further, the decreased expression of NMDAR subunits such as NR2B in the hippocampus may contribute to the learning deficit after SAH [438].

Platelet aggregation in parenchymal vessels leads to the formation of microthrombi and the release of glutamate—the platelet signaling molecule [439]. Platelet-derived glutamate around the microthrombi reduces glutamate receptor type 2 (GluR2) expression on the neuronal surface and thus contributes to neuronal glutamate receptor dysfunction [440].

**Fig. 9:**
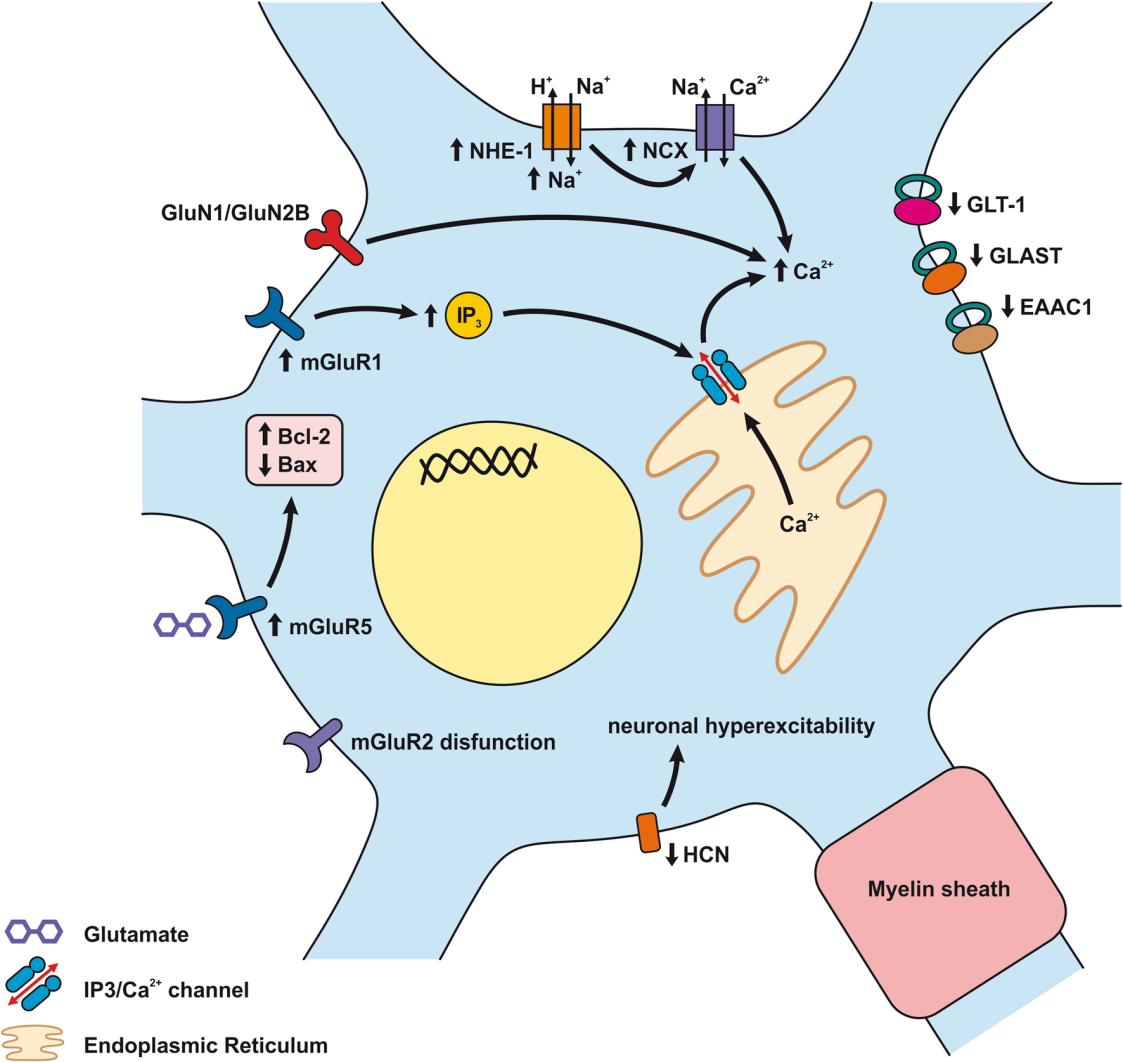
Glutamate induced neurotoxicity after SAH. Schematic illustration of channels and receptor dysfunction after SAH. Increased extracellular glutamate concentration generated after SAH leads to downregulation of GLT-1, GLAST, and EAAC1, resulting in neuronal degeneration. Dysfunction of mGluR2 may play a role in cognitive deterioration. Activation of extra-synaptic GluN1/GluN2B containing N-Methyl-D-aspartate receptors (NMDARs) leads to increased intracellular Ca^2+^ accumulation. Another glutamate receptor, mGluR1, activates IP3 and releases Ca^2+^ from the endoplasmic reticulum. Increased expression of NHE1 leads to intracellular accumulation of Na^+^, which causes Ca^2+^ entry via Na/Ca exchanger and thus contributes to excessive cytosolic Ca^2+^levels. Increased intracellular Ca^2+^levels lead to increased ROS generation resulting in neuronal injury following SAH. Extracellular glutamate activates mGluR5 and resulting in neuroprotection via increased expression of Bcl-2 and downregulation of Bax. Inhibition of HCN channels probably via exhausted NO signaling facilitates neuronal excitability after SAH

Activation of mGluR5, which seems to be neuroprotective, reduces caspase-3/NeuN-positive neurons in the cortex, up-regulates expression of Bcl-2, and down-regulates expression of Bax at 24 h after SAH [287]. However, You et al. found decreased Bcl-2 gene expression on days 1 and 3 and increased expression at 5 and 14 days after SAH [441]. Changes in extracellular glutamate levels were also found after SAH. Increased extracellular glutamate concentration was associated with downregulation of glutamate transporter 1 (GLT-1), glutamate/aspartate transporter (GLAST), and excitatory amino acid carrier 1 (EAAC1). These changes in glutamate transporters were accompanied by hippocampal neuronal degeneration during the first 7 days after SAH [442]. The mechanism of glutamate-induced neurotoxicity is summarized in Fig. 9 and Table 4.

#### Hippocampal damage after SAH

Despite the loss of long-term potential (LTP) at the Schaffer collateral-CA1 synapses in the hippocampus, there was no neuronal or other structural damage after SAH [443]. Neurexin-1β and postsynaptic membrane protein neuroligin-1 play an important role in synapse formation in the CNS. Decreased expression of neurexin-1β and neuroligin-1 in hippocampal and cortical neurons can contribute to cognitive dysfunction following SAH. Downregulation of neurexin-1β and neuroligin-1 was observed as early as 3 h after SAH, with the lowest expression at 72 h after SAH [444]. During the early stage of SAH, synaptic cell adhesion molecule 1 (SynCAM 1), a homophilic cell adhesion molecule at the synapse, was downregulated on days 1 and 3 but was back to normal on day 14 [445]. These findings are in accordance with the suggestion that there are mechanisms other than neuronal death responsible for LTP loss and learning deficit after SAH [446]. However, Guo et al. reported higher numbers of apoptotic neurons in the hippocampus 24 h after SAH, and this increased neuronal death was associated with elevated levels of MMP-9 [202]. Increased MMP-9 levels last up to 72 h after SAH but show a decreasing tendency. During the first 3 days after SAH in the hippocampus, neuronal anoikis was observed, which is a form of programmed cell death where cells lose their attachment to the extracellular matrix caused especially by laminin cleavage [447].

In contrast, Thal et al. found no neuronal loss or changes in cellular morphology in the hippocampal CA1-3 region up to 72 h after SAH and suggested that these changes are more pronounced in later time points after SAH [448]. This observation is in accordance with decreased cholinergic basal forebrain neurons as well as hippocampal and neocortical cholinergic terminals at the later stage between 4- and 14-days following application of blood into the subarachnoid space. Saline injection produced no significant changes [449].

Increased phosphorylation of Akt (on serine-473) and GSK3β (on serine-9) in the late phase was found in the hippocampus, confirming hippocampal neuronal injury in the later stage of SAH. However, phosphorylation of these proteins was accelerated and was correlated with acute brain injury in the cortex [450].

These observations partially correlate with the observation that blood and its degradation products mainly cause neuronal damage in the basal frontal and temporal lobes. On the other hand, vasospasm, hypoxemia, hypotension, as well as low CPP lead to neuronal apoptosis that, although is widely distributed in the brain, occur mainly in the hippocampus in the later phase of SAH [451]. It has been reported that the hippocampal CA3 region is the most sensitive to SAH-induced neuronal damage [452]. Neuronal death in the granule cell layer of the hippocampal dentate gyrus is predominantly apoptotic, whereas the hippocampal pyramidal cells usually showed necrotic death [453].

Changes in the hippocampus are induced not only by blood products after SAH but also by increased ICP. A sudden increase in ICP activates calmodulin-dependent protein kinase IIα in the hippocampus, which leads to phosphorylation of the Ser-847 of neuronal NOS 1 h after SAH. The consequent decrease in the enzymatic activity of neuronal NOS prevents excessive production of harmful NO [454]. Rapid ICP increase may also affect neuronal survival in the first seconds after SAH. It was found that rapid blood injection decreased immunoreactivity of NeuN 6 h after SAH in the dentate gyrus but not in CA3/CA4, CA1, and cortical neurons. Compared to slow application, a sudden ICP increase produced by rapid saline injection leads to decreased numbers of NeuN positive cells in the hippocampal CA1 region. This finding suggests that there might be some association between neuronal damage and the ICP spike after SAH [379].

Moreover, a sudden ICP increase induces neuronal NOS (nNOS) and protein kinase A (PKA) phosphorylation at Ser1412 in the hippocampal dentate gyrus already 1 h after either SAH induction or saline application. The excess NO produced by Ser1412 phosphorylated nNOS gets converted to peroxynitrite, causing neuronal cell damage [455]. A triphasic pattern of change was observed in nNOS-1 and inducible NOS-2 over 96 h after experimental SAH. The number of NOS-1 positive cells increased initially (between 1 and 3 h), gradually decreased to below control values between 6 to 72 h, and got back up to control values 4 days after SAH. Similarly, the number of NOS-2 positive cells increased at 1 h, decreased to control values at 24 h, and increased above control values 4 days after SAH [456].

#### Blood induced neurotoxicity

In vitro studies confirmed that Hb at clinically relevant concentrations is toxic to cultured neuronal cells [457, 458]. Following SAH, the release of OxyHb induces ERK phosphorylation and increases proapoptotic p53 by upregulating c-Myc [399]. OxyHb could increase phosphatidylcholine-specific phospholipase C (PC-PLC) in cultured neurons, which mediates neuron apoptosis probably by activating the NF-κB signaling pathway [459]. Most non-heme iron in the brain is bound to ferritin as Fe^3+^ and is localized mainly in the neurons and microglial cells after SAH. Iron deposition causes oxidative injury leading to brain edema and neuronal death, and brain atrophy after SAH [460]. OxyHb released from a subarachnoid clot can scavenge NO and destroy nNOS expressing neurons due to its neurotoxic effect. Reducing the availability of NO leads to vasoconstriction [178]. During the first hour after SAH, when NO is depleted, NOS synthetic potential remains stable but subsequently increases over the next few hours [456].

Toll-like receptors (TLRs) play an important role in the development of pro-inflammatory and pro-apoptotic responses after SAH. TLR4 may be necessary for neuronal apoptosis marked by TLR4–associated activator of interferon (TRIF), mainly in the late phase of SAH [280]. TLR-associated inflammation may be amplified by cortical spreading depolarization (CSD) after SAH. CSD induces the release of HMGB1, a TLR ligand, potentiating the inflammatory reaction [461]. HMGB1 is released passively from necrotic cells and actively from cortical neurons as early as 2 h after induction of SAH [370]. The extracellular HMGB1 serves as a DAMP and potentiates inflammation through its interaction with TLR2, TLR4 as well as with the receptor for advanced glycation end products (RAGE). HMGB1 activates the RAGE and the downstream MAPKs and NF-κB signaling pathways [370, 462].

The release of ATP and other molecules known as DAMPs is facilitated by Pannexin-1 after SAH. Pannexin-1is a membrane channel-forming protein and is expressed mainly on neurons and microglia. DAMPs such as ATP and other molecules released after Pannexin-1 stimulation are recognized by TLR2/TLR4 and mediates the NF-κB inflammatory response. Moreover, ATP as a danger signal binds to the P2X7 receptor (P2X7R), which is upregulated after SAH, leading to the activation of NLR family pyrin domain containing 3 (NALP3), caspase-1 as well as HMGB1 release, and thus contributes to the development of inflammatory response after SAH [271].

#### Cortical spreading depolarization after SAH

Several studies have shown that CSD contributes to neuronal death after SAH [463, 464]. CSD leads to microvascular spasms instead of vasodilation, and the subsequent ischemia delays cortical repolarization resulting in widespread cortical necrosis after SAH [465]. It has been found that CSD begins 1 ± 2.2 min after SAH induction of and repolarization occurs within 2.3 ± 1.2 min. The direction of CSD is from the frontal lobe cortex in the direction of the occipital lobe cortex at a rate of 3 mm per minute [466]. CSD is a multifactorial phenomenon with some degree of contribution from glutamate, ATP, extracellular K^+^ release, intracellular Ca^2+^ accumulation, as well as local anoxia [332].

It was experimentally found that local application of K^+^ and hemoglobin to the cortex to mimic hemolysis after SAH caused CSD, which led to spreading ischemia and widespread neuronal necrosis [467, 468]. Similarly, when fresh blood is applied, sulcal clot thicknesses were associated with greater CSD and the probability of recurrence [469]. Neuronal hyperexcitability in the hippocampal CA1 region contributes to CSD development. Increased neuronal excitability may be induced by Hb and the inhibition of hyperpolarization-activated/cyclic nucleotide-gated (HCN) channels. It was suggested that exhausted NO signaling in the CA1 region inhibits HCN channels and facilitates neuronal excitability after SAH [436].

**Fig. 10:**
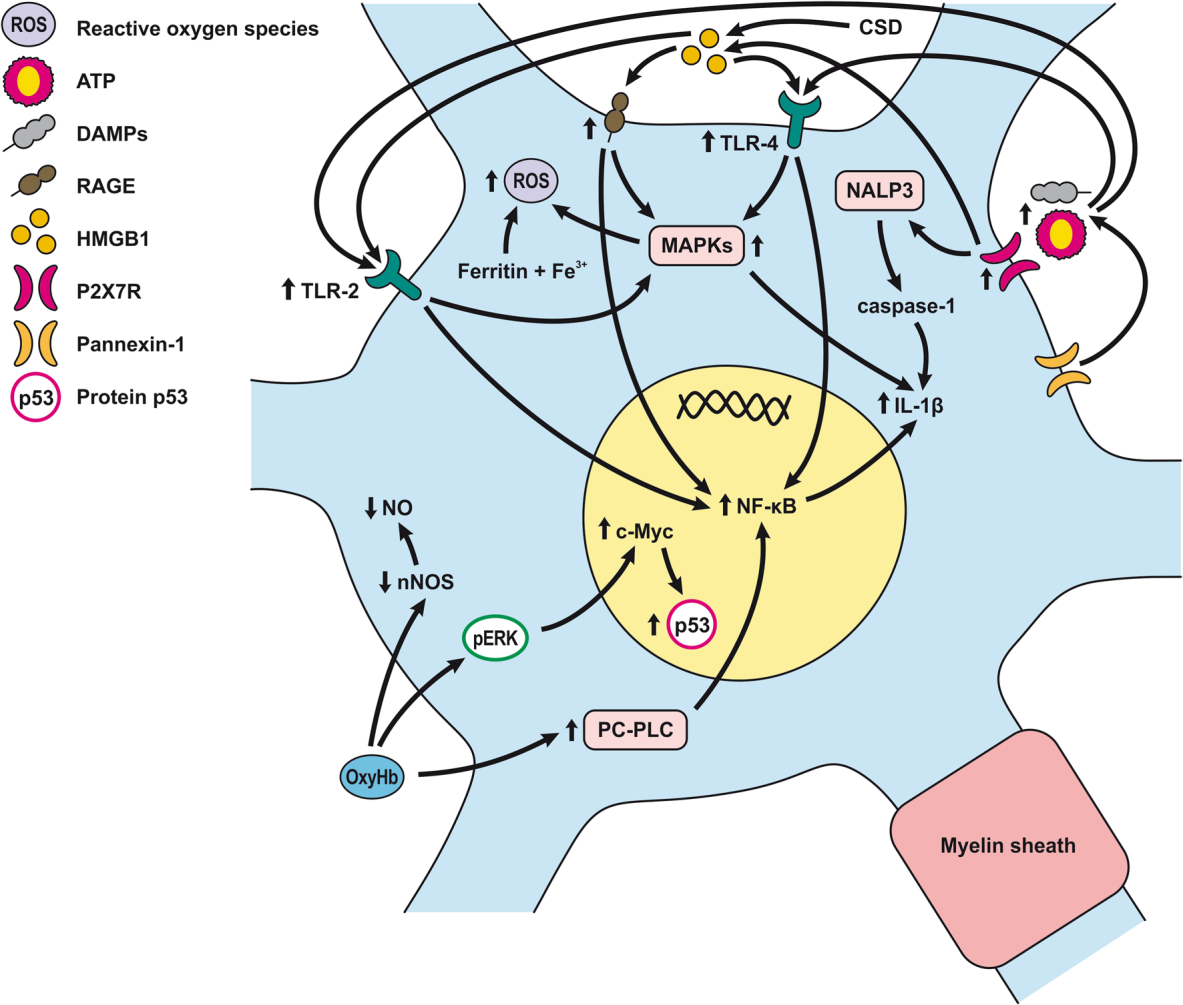
Blood induced neurotoxicity. Schematic illustration of blood and DAMPs induced neurotoxicity. OxyHb leads to phosphorylation of ERK that increases the pro-apoptotic p53 through the up-regulation of c-Myc. Other effects of OxyHb, such as increased PC-PLC mediating the NF-κB signaling pathway and scavenging of NO and destruction of nNOS expressing neurons, contribute to neuronal injury and vasoconstriction of blood vessels after SAH. DAMPs, including ATP and HMGB1, interact with TLR2, TLR4, and RAGE that activate the NF-κB and the MAPK pathways resulting in neuroinflammation, development of ROS, and neuronal death. HMGB1 is released due to CSD, passively from necrotic cells and actively from cortical neurons through the stimulation of P2X7R receptors. Activation of P2X7R receptors by ATP released from Pannexin-1 leads to the activation of NALP3 and caspase-1, contributing to the development of neuroinflammation. The iron storage protein ferritin binds most non-heme iron as Fe^3+^ ions. This iron deposition contributes to oxidative stress and neuronal death after SAH

The activation of neural progenitor cells can promote spontaneous recovery following SAH. Brain tissue samples from patients after SAH are positive for neural proliferation markers like SRY-Box transcription factor (SOX)-2 and Musashi. However, it remains questionable whether the newly formed neurons are functional [470]. On the other hand, neurogenesis decreased 1 day after experimentally induced SAH, reaching a minimum at day 3 and then increased gradually. The progenitor cells migrate from the subgranular zone to the granule cell line in the hippocampus and differentiate into mature neurons as early as 14 days after SAH induction [471]. These neuronal progenitors were also detected in the subventricular zone and the dentate gyrus, where they were decreased at 3 days and recovered to control numbers at 7 days after SAH. The majority of newly proliferated cells differentiated into neurons and migrated into the granular cell layer of the dentate gyrus within 30 days [472]. Blood-induced neurotoxicity is summarized in Fig. 10 and Table 4.

### Reaction of vascular smooth muscle cells to SAH

#### Morphological changes in vascular smooth muscle cells following SAH

Vascular smooth muscle cells (VSMC) are key cellular components involved in SAH pathophysiology. Vasospasms induced by VSMC contraction lead to hypoperfusion of brain tissue and result in the development of ischemic brain injury [473]. Morphological examination after SAH showed that VSMC have shortened, rounded and dystrophic morphology with numerous vacuoles and condensed chromatin [474]. Moreover, upon application of OxyHb as one of the degradation products of erythrocytes, VSMC lose their shape to become irregular, and the plasma and nuclear membrane disintegrate, leading to loss of the complex internal structure. These changes, along with the apoptosis and necrosis caused by blood and its degradation products, result in reduced VSMC density following SAH [475, 476].

#### SAH induced changes in calcium and potassium content of vascular smooth muscle cells

Despite their lowered numbers, VSMC play a major role in vasoconstriction, and the development of vasospasm as one of the main complications of SAH.

The predominant physiological consequence of SAH is the accumulation of intracellular Ca^2+^ leading to VSMC membrane depolarization which results in contraction and subsequent vasospasms. Membrane ion channels and G protein-coupled receptors are involved in Ca^2+^ dependent contraction [477, 478].

One of the many mechanisms of blood-induced increase in calcium level following SAH is activation of matrix metalloprotease and production of heparin-binding EGF-like growth factor, which activates the EGFR tyrosine kinase. This then leads to the internalization of voltage-gated potassium (K_v_) channels and the suppression of voltage-dependent outward K^+^ currents in cerebral artery VSMC. Decreased K_v_ channels may cause membrane depolarization and enhance Ca^2+^ influx via voltage-dependent Ca^2+^ channels (VDCCs), resulting in vasoconstriction [479–482]. The mRNA and protein of K_V_2.1 and K_V_2.2 channels were decreased 3 and 7 days following SAH [483, 484]. However, several types of potassium channels are involved in the excitability of VSMC after SAH. The function of ATP-sensitive potassium channels (sKATP) and the currents through them are inhibited after SAH. Modification of sKATP makes the channel open at a higher level of suprathreshold stimulation, which leads to a reduction in their activity followed by VSMC contraction. sKATP contains two subunits, an inward-rectifying K^+^ channel (Kir)-6.1 and a sulphonylurea receptor (SUR2B). These channels maintain basal VSMC tension and increased blood flow during hypoxia or ischemia. However, SAH leads to alteration of Kir6.1 72 h after SAH promoting the contraction of smooth muscle [485]. Large conductance calcium-activated potassium (BK) channels expressed in the VSMC also play a role in vasoconstriction after SAH [486]. Kv7 (KCNQ) channels are suppressed by different vasoconstrictors associated with vasospasms like serotonin, endothelin, and vasopressin. Kv7 current suppression is probably mediated by the activation of PKC that activates Gq/11-coupled receptors resulting in the inhibition of Kv7 channels [487, 488].

BK channels respond to depolarization as well as increased intracellular Ca^2+^ by opening and releasing K^+^ to the extracellular space [489]. However, mRNA expression of the BK channel β1 subunit decreases following SAH. Reduction in the mRNA for the BK-β1 subunit and also Kv 2.2 was correlated with the degree of vasospasm [483]. It seems that SAH-induced reduction of β1-subunit could reduce the sensitivity of BK channels to Ca^2+^, shift its voltage dependence to more depolarized potentials and thus contribute to vascular contraction [490]. BK channels, as well as astrocytic endfeet, increase K^+^ in the perivascular space. Once the concentration of K^+^ exceeds an approximately ∼20 mM threshold, vasoconstriction occurs instead of vasodilation [329]. Several potent vasoactive molecules, including angiotensin II, thromboxane A2, 5-hydroxytryptamine (5-HT), as well as heme and bilirubin oxidation end products (BOXes), are able to inhibit BK channels and are released following SAH [491, 492].

Nevertheless, the frequency of Ca^2+^ release-spikes from the sarcoplasmic reticulum is reduced after SAH. Altered Ca^2+^ spikes lead to reduced BK channel activity and increased voltage-dependent Ca^2+^ channels (VDCCs) activity resulting in elevated global cytosolic Ca^2+^ and membrane depolarization [493]. Reduction of Ca^2+^ spike frequency results from reduced expression of ryanodine receptor type 2 (RyR-2) Ca^2+^-release channels located on the sarcoplasmic reticulum and increased RyR-2- stabilizing protein, FKBP12.6 [494]. Moreover, Ca^2+^ activated K^+^ channels are inhibited by 20-hydroxyeicosatetraenoic acid (20-HETE) via activation of PKC and Rho kinase. Angiotensin II, endothelin, ATP, and serotonin are released following SAH and induce the formation of 20-HETE. On the other hand, the formation of 20-HETE is inhibited by NO, CO, and superoxide radicals that are also generated after SAH [495]. However, it has been reported that 20-HETE blocks K^+^ channels and increases cerebral vascular tone after SAH [496]. Synthesis of 20-HETE is stimulated by 5-HT_1B_ activation by 5-HT. This cascade potentiates the vasoconstriction response of 5-HT in VSMC after SAH [497].

In addition to K^+^, the intracellular level of Ca^2+^ plays a determining role in smooth muscle contraction. It is known that VDCCs such as L-type Ca^2+^ channels are involved in the pathophysiology of vasospasm after SAH [498, 499]. VDCC currents are increased 72 h after SAH and may play a significant role in the development of cerebral vasospasm following SAH [500]. L-type Ca^2+^ channels could be inhibited by NO, and this suppression of Ca^2+^ current may be one of the mechanisms of NO-induced relaxation of VSMC contraction [501]. Moreover, the NO-cGMP pathway also plays an important role in smooth muscle dysfunction following SAH, and the alteration in this pathway is due more to changes in cGMP levels rather than to the disruption of the NO-cGMP downstream pathway [502].

Interestingly, the expression of high voltage-activated L-type VDCCs subunits was decreased following SAH. On the other hand, the expression of low voltage-activated T-Type VDCCs subunits was increased. This reduction in L-type currents after SAH may be one of the reasons for the low efficacy of L-type channel antagonists such as nimodipine [503]. In addition to L-type VDCC, OxyHb released after SAH induces the expression of R-type Ca^2+^ channels, reduces the sensitivity of L-type Ca^2+^ channels, and also contributes to reduced function of L-type VDCC antagonists [504, 505]. Despite these observations, Nystoriak et al. report an increase in L-type VDCC mediated Ca^2+^ influx in parenchymal arteriolar VSMC after SAH [506]. Vasoconstriction following SAH may also be enhanced by vascular superoxide that increases Ca^2+^ entry probably through L-type channels [507]. In addition to ROS, elevation in intravascular pressure leads to greater membrane potential depolarization and pressure-dependent contraction through the increased activity of L-type Ca^2+^ channels [506]. Besides L-type Ca^2+^ channel-induced entry of Ca^2+^ and the metabotropic Ca^2+^ release from the sarcoplasmic reticulum, Ras homolog family member A (RhoA)/ROCK activation also contributes to VSMC contraction. OxyHb activates RhoA/ROCK, which enhances VSMC contraction. Increased intracellular Ca^2+^levels, along with ROCK activity, may participate in the observed increase in tonic VSMC contraction on day 5 after SAH [508, 509].

The ROCK family promotes smooth muscle contraction by phosphorylation of myosin light chain phosphatase (MLCP) at the myosin-binding subunit and inhibition of its enzymatic activity [151, 510, 511]. Inhibition of VSMC phosphatase was suggested as one of the mechanisms contributing to SAH-induced vasoconstriction [512]. Bilirubin oxidation products (BOXes), and biliverdin that are present in the CSF after SAH, activate the alpha and delta PKC isoforms as well as RhoA in arterial VSCM leading to the activation of ROCK which phosphorylates and inactivates MLCP [513]. Moreover, BOXes increases contractile protein myosin ATPase that contributes to VSMC contraction and luminal constriction [514]. Apart from the development of vasospasm, BOXes may play a role in vascular remodeling after SAH [515]. Apart from BOXes, OxyHb is also involved in activating PKCε and, to a lesser extent of PKCα [516]. It was found that PKCα and PKCδ play a pivotal role in the development of vasospasm after SAH [517].

Sphingosyl-phosphorylcholine (SPC) is another potent vasoconstrictor that is increased after SAH in the CSF, where it also contributes to the ROCK-mediated phosphorylation and inactivation of MLCP [518, 519]. Further, SPC activates p38MAPK and increases the activity of proinflammatory NF-κB and CCAAT-enhancer-binding proteins (C/EBP), a family of transcription factors in VSMC [520].

#### Inflammatory response of vascular smooth muscle cells after SAH

Stress, along with several molecules associated with SAH such as OxyHb, BOXes, endothelin 1 (ET-1), ATP, EGF, PDGF, IL-1β, TNF-α, activate membrane receptors leading to MAPK activation in the VSMC. The substrates for MAPK are caldesmon and calponin, that block myosin binding to actin and inhibit actin-dependent myosin ATPase activity in the VSMC. Phosphorylation of these proteins by MAPK reverses the inhibitory effect on VSMC contraction after SAH [521]. In line with this, calponin degradation following SAH was reported, indicating a role for it in the regulation of VSMC contraction [522].

NF-κB, an ancient protein transcription factor, and C/EBP proteins are involved in the regulation of inflammation, cell proliferation, and cell survival following SAH. Activation of these transcription factors leads to increased expression of the chemokine monocyte chemoattractant protein-1 (MCP-1/CCL2) as well as other pro-inflammatory molecules like TNF-α and IL-1β, which contribute to the development of inflammation following SAH [520, 523].

It has been suggested that the initial drop in CBF induced by SAH triggers molecular cascades that result in vasoconstriction. The presence of blood in the subarachnoid space under higher pressure following SAH leads to the activation of integrins, mechanoreceptors, and plasma membrane receptors that activate the downstream Raf-mitogen-activated protein kinase (MEK1/2)–ERK1/2 pathway and consequently transcription factors including STAT3.

The decreased arterial wall tension reduced blood flow. The presence of blood in the subarachnoid space following SAH results in the activation of integrins, mechanoreceptors, and plasma membrane receptors that activate the downstream Raf-mitogen-activated protein kinase (MEK1/2)–ERK1/2 pathway and consequently transcription factors, including STAT3. These transcription factors then induce increased expression of pro-inflammatory cytokines like TNF-α, IL-1β, IL-6, as well as MMPs, iNOS, and receptors for angiotensin II type 1 (AT1) endothelin B (ET_B_), 5-HT_1B,_ and TX_2a_ [524–527].

The increased expression of endothelin A (ET_A_) led to VSMC contraction after SAH [218, 528]. Stimulation of the ET_A_ receptor activates PKC and Ras homolog family member A (RhoA)/Rho kinase, leading to increased phosphorylation of MLCP, contributing to cerebral vasospasm development [529]. Expression of other contractile receptors for ET_B_, 5-HT_1B,_ and AT1 reached a maximum at 48 h after SAH. Along with the expression of the 5-HT_1D_ receptor in VSMC contributing to increased contractility after SAH [530], these changes in receptor expression were associated with cerebral blood flow reduction [267].

Pro-inflammatory cytokines, growth factors, and oxygen radicals cause dedifferentiation of VSMC, giving rise to the so-called phenotypic transformation that contributes to cerebral vasospasm [531]. Phenotypic transformation changes VSMC from the contractile phenotype (under normal physiological conditions) to the synthetic phenotype in which vascular tone becomes difficult to regulate. The synthetic phenotype is characterized by increased proliferation of extracellular matrix components, including an excess of collagen leading to vascular wall thickening and stenosis, narrowing of the lumen, and reduced expression of contractile genes [532, 533]. Expression of embryonic smooth muscle myosin heavy chain (SMemb), a marker of the synthetic phenotype, increased at 24 h after SAH [534, 535]. These phenotypic changes may explain the sustained VSMC contraction that can be seen for more than 2 weeks after SAH [536, 537].

Mammalian target of rapamycin (mTOR) and proliferating cell nuclear antigen (PCNA) may play a role in regulating growth, proliferation, survival, and protein synthesis in VSMC. This suggestion is supported by increased expression of mTOR and PCNA in contracted VSMC seen at 7 days after SAH induction [538]. Moreover, PCNA expression increased simultaneously with p-ERK1/2 and peaked on day 7 after SAH indicating a prolonged inflammatory response [539].

Remodeling the vascular wall during phenotypic transformation was also associated with the increased tenascin-C (TNC) levels found mainly in the VSMC layers. The extent of this increase was higher in patients with vasospasm [540]. The TNC protein level was elevated on day 1 and decreased on day 3 after SAH. Immunoreactivity of another matricellular protein, osteopontin (OPN), was decreased on day 3 after induction of SAH [541]. The beneficial effect of OPN is probably mediated upon VSMC phenotypic transformation through the integrin-linked kinase (ILK) and Rac-1 that preserves VSMC phenotype [535, 542]. Remodeling VSMC during phenotypic transformation was also characterized by the expression of beta-actin rather than alpha-actin. Vascular remodeling after SAH, as well as cellular growth, could be mainly attributed to increased beta-actin mRNA expression [543]. The decreased alpha-actin intensity in VSMC probably contributes to the VSMC shift towards a more synthetic (less differentiated) phenotype [544].

#### Remodeling of vascular smooth muscle cells following SAH

SAH stimulates receptors, including platelet-derived growth factor receptor β (PDGFR-β), that are expressed on VSMC. The PDGFR-β signaling cascade activates IRF9/SIRT-1/NF-κB pathway and contributes to VSMC phenotypic transformation during EBI after SAH [545]. Platelets, macrophages, and endothelial cells release PDGF-β that peaks 7 days after SAH and activates the PDGF receptor on VSMC. Moreover, activation of PDGF receptor leads to activation of cellular proliferation pathways such as MAPK, ERK1/2, PI3K, and Rho-ROCK resulting in the intracellular accumulation of Ca^2+^, and the hyperplasia and hypertrophy of VSMC after SAH [546, 547]. However, it seems that increased PI3K activity (rather than elevated PI3K protein expression) contributes to VSMC contraction on day 7 following SAH induction [548]. Prolonged increases in PDGF-β upregulates the PDGF-β receptor, which increases VSMC contractility in response to PDGF-β [216]. Upregulation of Rho kinase activity after SAH also contributes to the contraction of VSMC and the increased sensitivity of myofilaments to Ca^2+^, especially to ET-1 [549]. Rho kinase also augmented contraction in response to serotonin in the VSMC following SAH [550]. Borel et al. found that vascular and perivascular proliferation associated with PDGF protein was mainly associated with regions affected by thrombi [551]. PDGF-induced contraction is dependent mainly on Ca^2+^ elevation through phospholipase C-γ. Activation of phospholipase C-γ and subsequent IP_3_ production leads to increased MLC phosphorylation and contraction of VSMC [552]. PDGF receptor activation could be inhibited by caveolin-1, a primary caveolae protein, which shows strong anti-mitogenic and anti-proliferative effects. Decreased expression of caveolin-1 in VSMC was found at 7 days, which then recovered on day 14 after SAH induction [553]. Phenotypic switching of VSMC from the contractile to the synthetic phenotype and consequent vascular remodeling is partly regulated by HMGB1, which acts by activating the PI3K/ Akt pathway. HMGB1 expression peaked at 72 h and remained elevated 5 days after SAH [554].

It seems that signaling cascades downstream of EGFR also play a role in the pathophysiology of SAH. Vasoactive molecules like ET-1, thrombin, and angiotensin II activate EGFR and its intracellular protein tyrosine kinase via G protein-coupled receptors, resulting in ERK1/2 activation and vasoconstriction [555]. Thrombin is one of the major activators of protease-activated receptors (PAR) after SAH [556]. Among other effects, thrombin activates PAR-1, -3 and, -4 and induces contraction of VSMC mainly through PAR-1 [557]. SAH was accompanied by up-regulation of PAR-1 and hyper-responsiveness to thrombin. Moreover, thrombin leads to prolonged contraction of VSMC by persistent activation of PAR-1 caused by impaired feedback inactivation of PAR [558]. The mitogen-activated protein kinase (MEK)/ERK1/2 pathway plays an important role in VSMC structural changes. Activation of the MEK/ERK cascade results in increased expression of contractile receptors such as angiotensin II type 1 (AT1), ET_B_, 5-HT1_B_, TX2_a_ and thus potentiates vasoconstriction [559–561]. ERK1/2 could also be stimulated by endothelin 1 (ET-1) through transactivation of EGFR protein tyrosine kinase leading to ERK1/2 stimulation, which contributes to VSMC contraction [562]. Stimulation of VSMC by endothelin A (ET_A_) receptor and ET_B_ activated by ET-1 results in myosin light-chain kinase (MLCK) activation and VSMC contraction [563].

SAH increases the expression of ET-1 and enhances myofilament Ca^2+^ sensitization via protein kinase C*α* (PKCα) and the ROCK2 signaling pathway. It seems that PKCα is associated with transient phosphorylation, whereas ROCK2 mediates prolonged phosphorylation of MYPT1 at T853 and possibly also at T696 [564]. PKCα and PKCδ are activated and involved in ET_B_ and 5-HT1_B_ receptor upregulation following SAH [565]. ET-1 stimulates store-operated Ca^2+^ channel (SOCC) and nonselective cation channels-2 (NSCC-2) via phospholipase C leading to Ca^2+^ influx. Extracellular Ca^2+^ influx thus contributes to ET-1 induced VSMC contraction after SAH [566]. Further, it was reported that ET-1 transactivates EGFR protein tyrosine kinase resulting in ERK1/2 stimulation [562].

P2 nucleotide/purinergic receptors play a role in the accumulation of intracellular Ca^2+^. The expression of P2 receptor subtype P_2X1_ mRNA was lowered 3 days after SAH and recovered between day 5 and 7 after SAH. On the other hand, expression of the P2Y1 subtype was increased 5 days after SAH returning to normal values at day 7 following SAH [567]. Continuously elevated intracellular Ca^2+^ levels result in vasospasm after SAH probably via the activation of μ-calpain and Ca^2+^/calmodulin-dependent MLCK phosphorylation of the myosin light chain (MLC) [478]. The contractile phenotype (more differentiated) of VSMC is partially maintained following SAH by peroxisome proliferator-activated receptor β/δ (PPARβ/δ) that induces PI3K/ Akt activation. However, Hb causes a decrease in PPARβ/δ expression and thus contributes to vascular remodeling [568]. The beneficial effect of PPARγ is also mediated by blocking TLR4 activation and cytokine release from VSMC [569]. Increased expression of TLR4 in VSMC occurs 3 and 5 days after SAH induction. Elevated expression of TLR4 in VSMC likely contributes to inflammation of the vascular wall and thus influences VSMC contraction [570].

Moreover, there is evidence that increased MMP-9 expression and decreased expression of collagen IV and V may enhance contractility of VSMC, resulting in vasospasm after SAH [571]. Expression of MMP-9, as well as that of tissue inhibitors of metalloproteinase -1 (TIMP-1), was elevated 48 h after SAH. The imbalance between MMP-9 and TIMP-1 expression is probably caused by the activation of the MEK/ERK1/2 pathway after SAH [572]. Activation of the MEK/ERK signaling pathway was associated with increased expression of TNF-α, TNF receptor (TNF-R)-1 and -2. Stimulation of these receptors by TNF-α leads to activation of MAPK, and the subsequent transcription factors NF-κB and activator protein-1 (AP-1) induce the expression of pro-inflammatory molecules in VSMC such as IL-1α, IL-1β, and IL-8 [573, 574]. TNF-α is one of the major cytokines involved in the pathophysiology of SAH. In addition to its pro-inflammatory properties, it also enhances vascular tone by affecting the sphingosine-1-phosphate (S1P) signaling pathway. Increased bioavailability of S1P enhances its pro-constrictive effects. TNF-α activates sphingosine kinase 1 (Sphk1) gene expression that encodes S1P and inhibits S1P degradation partially via downregulation of the cystic fibrosis transmembrane conductance regulator (CFTR) [575].

Adenosine 1 (A1) receptor on the VSMC of the cerebral vasculature is considered to be the mediator of the adenosine response and is associated with vasodilation probably by maintaining normal iNOS and eNOS expression, opening K_ATP_ channels, or inhibiting N-, P- and Q-type Ca^2+^ channels [272]. However, Sehba et al. found that inhibition of adenosine A2 (A2_A_) receptors decreases ICP and the constriction of major vessels while increasing CPP and microvascular collagen-IV [576]. Therefore, its affecting adenosine receptors in order to attenuate brain injury after SAH remains controversial.

#### Vascular smooth cells contribute to neuroprotection following SAH

Apart from the predominantly negative and some controversial effects after SAH, there are also some mechanisms that contribute to neuroprotection. The increased expression of relaxin-1 (RLN1) following SAH causes vasodilatation, antifibrosis, anti-inflammation and probably also dilates arteries. Expression of RLN1 was increased on day 7 after SAH induction. However, expression of relaxin/insulin-like family peptide receptor-1 (RXFP1) was downregulated on day 3 after SAH and caused functional RLN1 reduction in cerebral VSMC [577]. On the other hand, OxyHb also induces VSMC expression of Nrf2 that upregulates expression of HO-1 and NAD(P)H: quinone oxidoreductase-1 (NQO1) after 48 h of OxyHb exposure. HO-1 and NQO1 have anti-inflammatory and antioxidative effects and maintain redox homeostasis [578]. When exposed to Hb and its breakdown products, cultured rat basilar artery VSMC responded by increasing HO-1 protein expression after 6 h. Increased expression of ferritin was observed up to 72 h which might be related to suppressing iron toxicity [579]. Transferrin, an iron-binding glycoprotein, may also play a role in the pathological cascades leading to vasospasm. Increasing transferrin concentration in the CSF following SAH induces iNOS mRNA in VSMC. This suggests that there might be some relationship between transferrin and cerebral vasospasm [580].

VSMC, as well as endothelium, contain D2-dopamine receptor (D2R) that mediate eNOS and iNOS as well as decreased intracellular Ca^2+^ concentrations after its stimulation by a D2R agonist following SAH [581]. Another receptor potentially involved in the pathophysiology of vasospasm is parathyroid hormone receptor-1 (PTH-R1). Expression of PTH-R1 mRNA was downregulated after 3 days and upregulated 14 days from induction of SAH. Stimulation of PTH-R1 on VSMC by PTH leads to the activation of adenylate cyclase, accumulation of cyclic adenosine monophosphate, and activation of PKCα, thereby decreasing Ca^2+^ influx through voltage-gated Ca^2+^ channels and resulting in vascular relaxation [582].

### The role of gender and sex hormones after SAH

Numerous studies have focused on the potential therapeutic effect of sex hormones such as 17β-estradiol (estrogen; E2), progesterone and testosterone. However, the impact of gender.

on pathophysiological cascades after SAH has not been extensively studied [583].

Endothelium-dependent vasodilatory response is greater in women than in men. Moreover, endothelial cells in women are more resistant to the effects of various signals mediating vasoconstriction. These differences in vasoreactivity are partially mediated by sex hormones and include increased eNOS synthesis, decreased levels of vasoconstrictor signals such as ET-1 and TXA2 [584]. In addition to vasoreactivity, female brain endothelial cells are more resistant to ischemic injury. The mechanism of endothelial cell resistance to ischemic damage is due in part to lower expression of soluble epoxide hydrolase and higher expression of epoxyeicosatrienoic acids (EETs) in females. A higher level of EETs inhibits ROCK activation induced by oxygen–glucose deprivation following ischemic injury [585].

Other positive effects of EETs may play a role in the pathophysiology of SAK, such as the inhibition of platelet aggregation and apoptosis, regulation of neurovascular coupling, decreased expression of PLA2, COX2 and PGE2, as well as increased expression of BDNF [273].

Vascular reactivity is important in patients after SAH. A few studies looked at vascular reactivity using sex hormones. The majority of them focused on functional outcome which is related to vasoreactivity and found no differences between women and men [583, 586]. However, an experimental study focused on gender differences in SAH pathology in rats found greater collagen-IV loss in males, which may be the result of a more severe inflammatory reaction. While the number of apoptotic cells increased (as revealed by caspase-3 activity), their number was greater in males when compared to females. This finding suggests that the initial impact of SAH is more severe in males than in females [587].

The major female sex hormone, E2, exhibits vasodilatory, anti-inflammatory, and neuroprotective properties. E2 maintains normal expression of eNOS and reduces expression of iNOS and ET-1, thus contributing to vasodilation. It was found that NFκB activation leads to increased iNOS expression. E2 induced downregulation of iNOS expression may act through disruption of NFκB/iNOS binding activity. Administration of E2 not only attenuates vasospasm and secondary ischemic damage but also reduces mortality after SAH [588–591].

The anti-inflammatory response of E2 is mediated by suppression of JNK activity and the subsequent decreased expression of TNFα. Moreover, E2 binds to estrogen receptor β (ERβ) leading to decreased expression of ICAM-1, VCAM-1, MCP-1, P-selectin, and cytokine-induced neutrophils chemoattractant-2β (CINC-2β) resulting in decreased leukocyte chemotaxis. The neuroprotective action of E2 is mediated via the increased expression of thioredoxin (Trx) which leads to reduced lipid peroxidation and caspase-3 activation. E2 neuroprotection is also mediated by its activating ERβ and the subsequent action of Ngb, which contributes to maintaining oxygen homeostasis in neurons. Ngb also offers protection against ROS-induced oxidative damage. Ngb has an anti-apoptotic effect as it inhibits the release of cytochrome c from mitochondria, and the increased expression of adenosine A2a receptor (A2aAR) and ERK1/2, as well as activating estrogen receptor α (ERα). Activation of ERα prevents suppression of the Akt signaling cascade in the dentate gyrus after SAH [590, 592–594]. Moreover, Akt induces phosphorylation of mTOR which promotes cell growth through the up-regulation of anti-apoptotic Bcl-2 [595, 596]. Despite increased Bcl-2 expression, the amount of pro-apoptotic Bax is not affected by E2 treatment [597].

Progesterone was shown experimentally to play a role in preventing vasospasm induced by SAH. Vasospasm is alleviated through the increased expression of eNOS via the upregulation of phospho-Akt protein expression [598]. In addition to increased eNOS, administration of progesterone also reduces synaptic injury by restoration of synaptic GluR1 levels and reduces microglial activation as measured by Iba-1 expression [599]. Progesterone contributes further to neuroprotection by reducing pro-inflammatory molecules such as IL-β, TNF-α, IL-6, ICAM-1 and MCP-1. Decreased expression of pro-inflammatory molecules is brought about by attenuating the TLR4/NF-κB signaling pathway in the cortex following SAH [600]. BBB disruption and edema formation after SAH may also be alleviated by progesterone and its down-regulating effect on MMP-9 and caspase-3 [601]. Despite these beneficial effects of female hormones inferred from experimental studies, it appears that elderly women have a higher risk of developing DCI than men. However, studies are not unanimous about whether developing of DCI is caused by menopause or not [602–605].

A neuroprotective effect in the central nervous system of testosterone, the main male sex hormone, has also been observed previously [583, 606]. Testosterone acts through ion channels and leads to the inhibition of L-type voltage-dependent Ca^2+^ channel (VDCC), and the opening of a voltage-dependent K^+^ channel following SAH. Their combined action leads to VSMC relaxation and vasodilation. Other possible mechanisms of testosterone vasodilatory activity are via reduced ROS formation, an anti-inflammatory effect and increased NO synthesis [607].

In males, increased expression of some beneficial genes such as Nos3 and Thbd contributes to the neuroprotective effect. Nos3 encodes eNOS and thus contributes to vasodilatation. Thbd encodes thrombomodulin which is expressed on the surface of endothelial cells and mediates anti-inflammatory and anti-coagulant effects [608, 609].

On the other hand, testosterone also increases TXA2 receptors in VSMC which may contribute to vasoconstriction [584].

## Potential drugs used in SAH treatment

Although SAH is currently considered treatable, it remains a condition associated with a high mortality rate [1]. In current practice, pharmacological treatment is limited to nimodipine, which should be administered to all patients after aneurysmal SAH as recommended in the 2012 guidelines [2]. However, continuous intra-arterial nimodipine infusion is associated with side effects such as increased levels of ICP, reduction of systolic and diastolic blood pressure, increase in infectious complications, and worsening of gastrointestinal tract motility [3, 4].

Therefore, over the past few years, preclinical research has been focused on potential active substances that could have beneficial effects on pathophysiological processes after SAH.

Endothelial cells, astrocytes, microglia, neurons, and VSMCs can be targeted by synthetic and semi-synthetic molecules as well as herbal substances, as was proved in experimental animal studies. Some of these drugs are commonly used in clinical practice to treat various pathological conditions, and some are only used experimentally (Tables 5, 6, 7, 8, 9). Obviously, different cells respond to SAH differently, and a single drug cannot sufficiently affect all components of the vasculo-neuronal-glial triad. On the other hand, some substances appear to act through different molecular mechanisms and may have a wide range of effects. In general, the purpose of these substances is to affect the main pathophysiological cascades after SAH that either contribute to neuroprotection or lead to neuroinflammation, BBB disruption, ROS formation, vasospasm, or cell death.

**Table 5:**
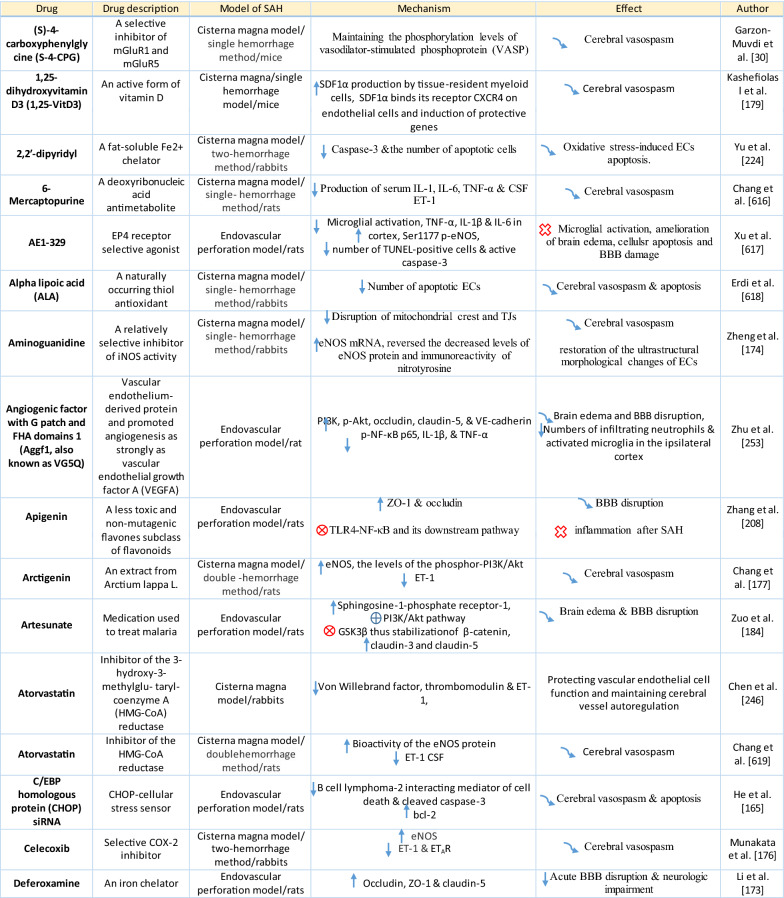

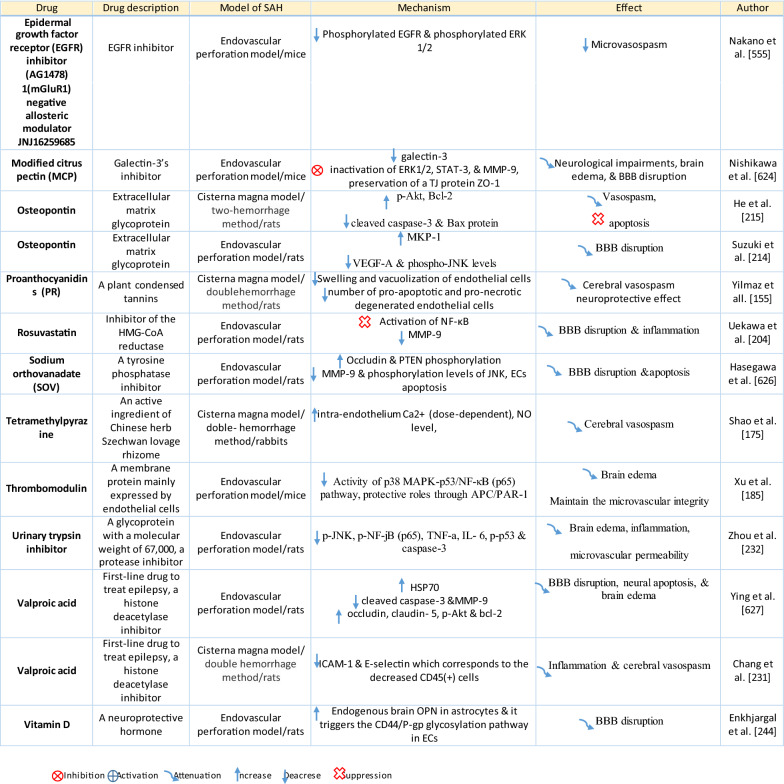
Potential drugs affecting endothelial cells after SAH—from 2010 to 2021

**Table 6:**
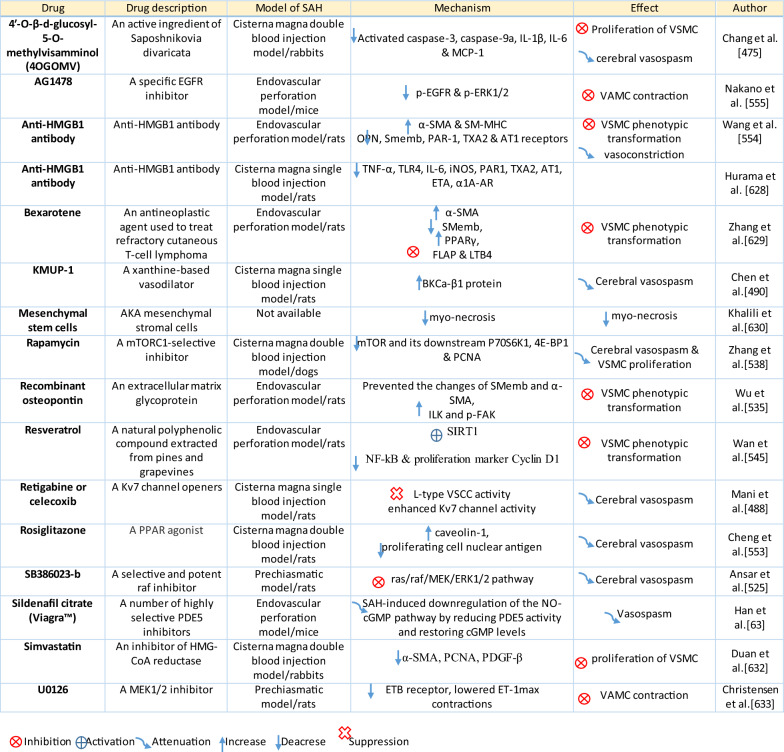
Potential drugs affecting vascular smooth muscle cells after SAH—from 2010 to 2021

**Table 7:**
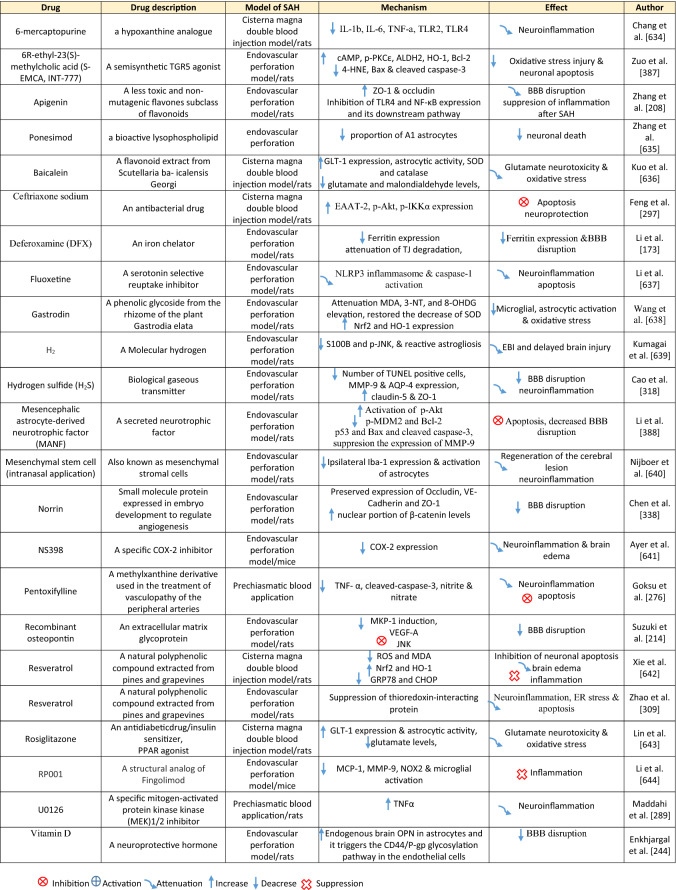
Potential drugs affecting astrocytes after SAH- from 2010 to 2020

**Table 8:**
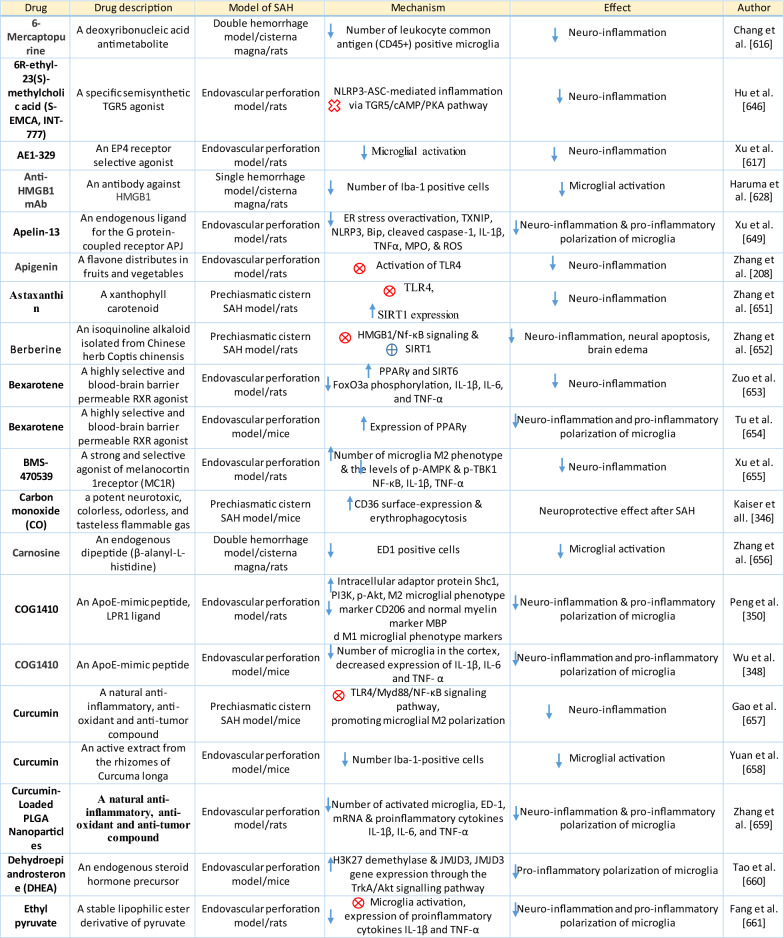

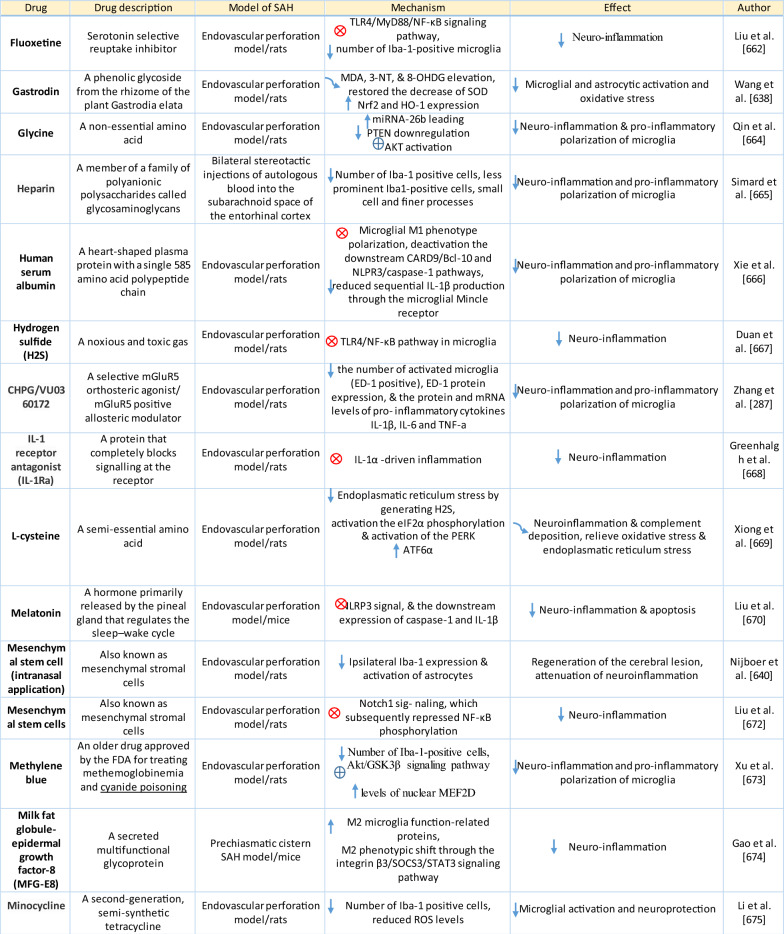

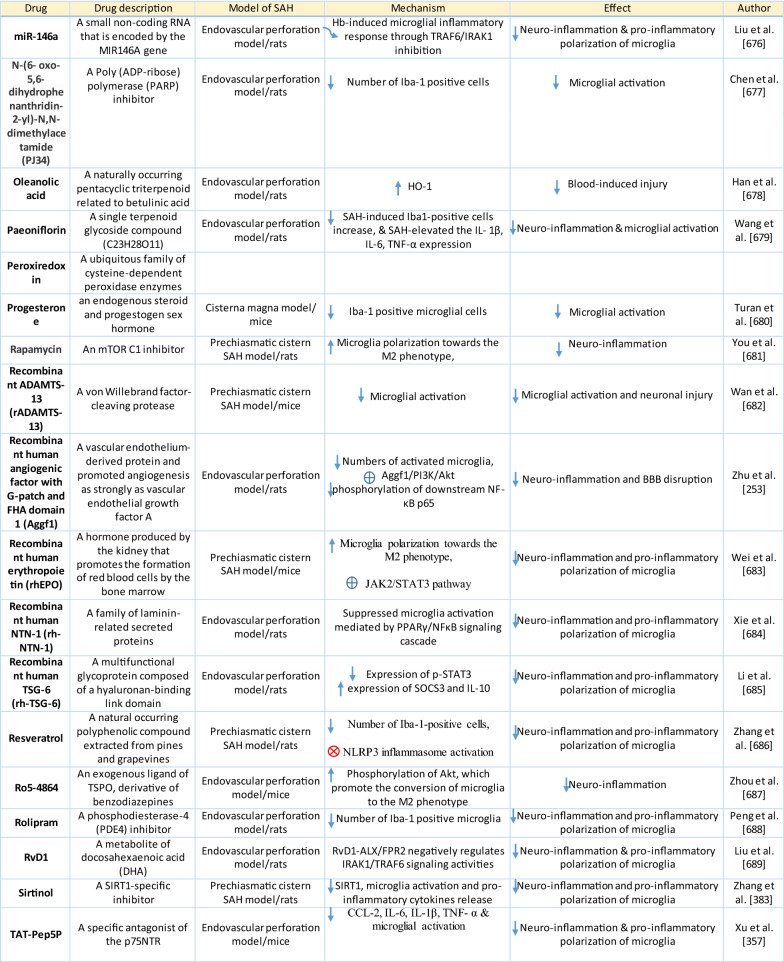

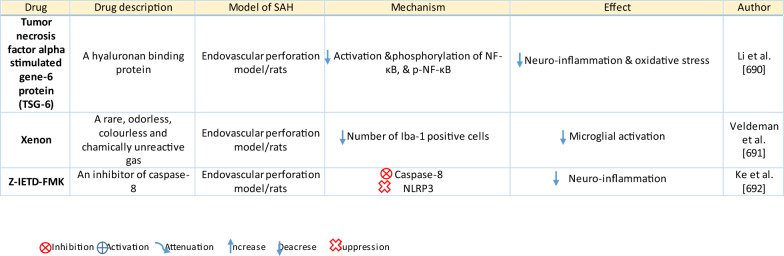
Potential drugs affecting microglia after SAH—from 2010 to 2021

**Table 9:**
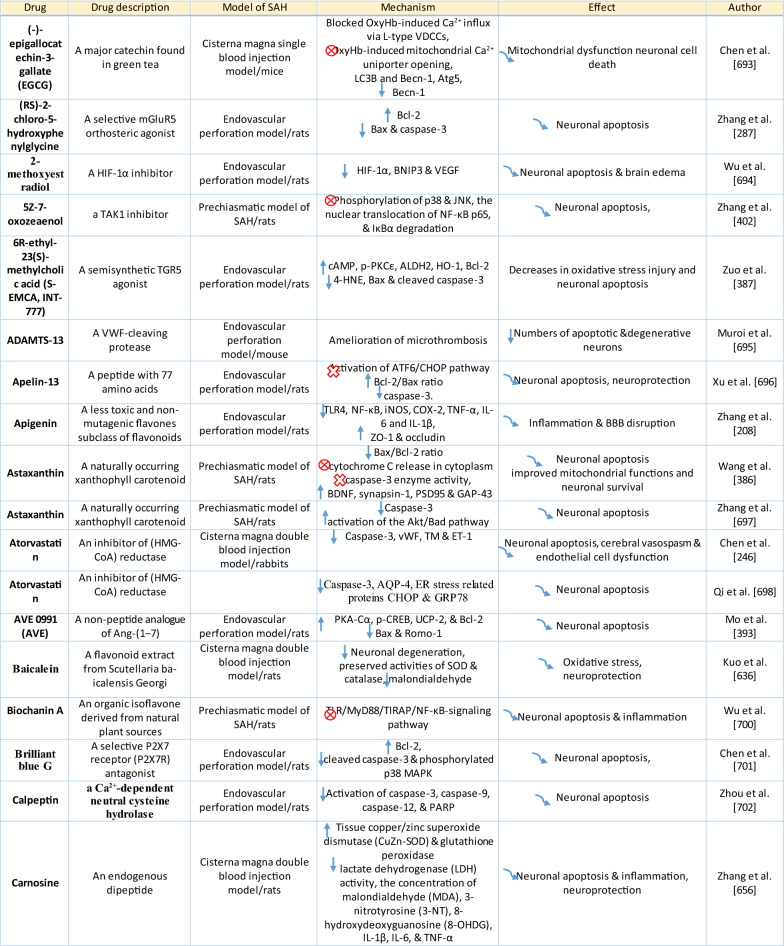

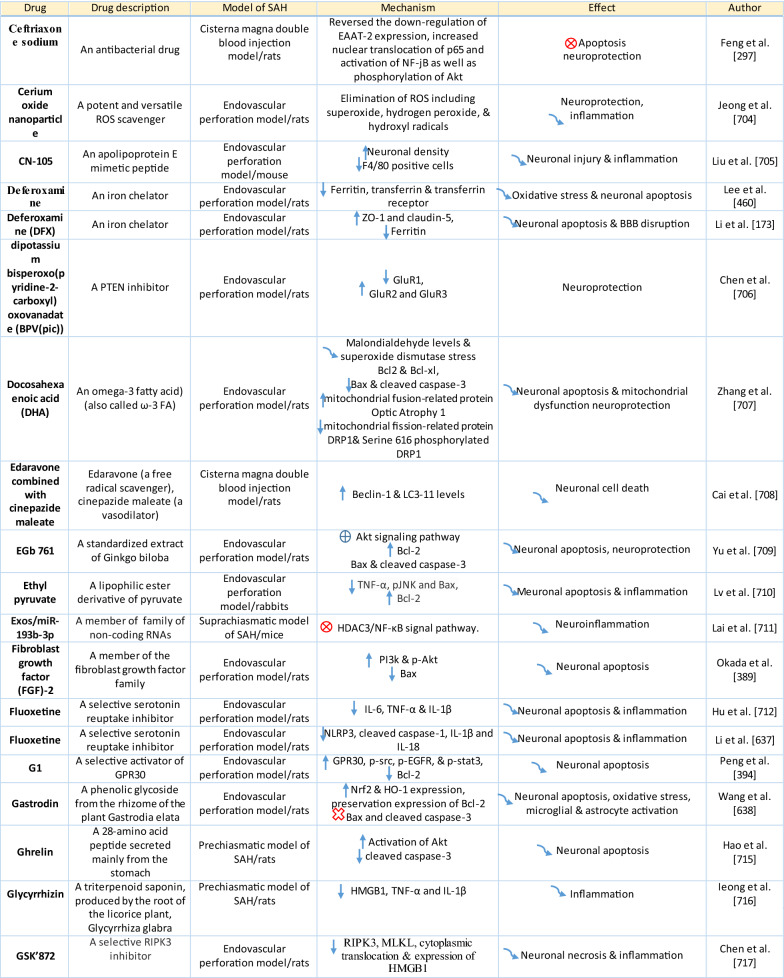

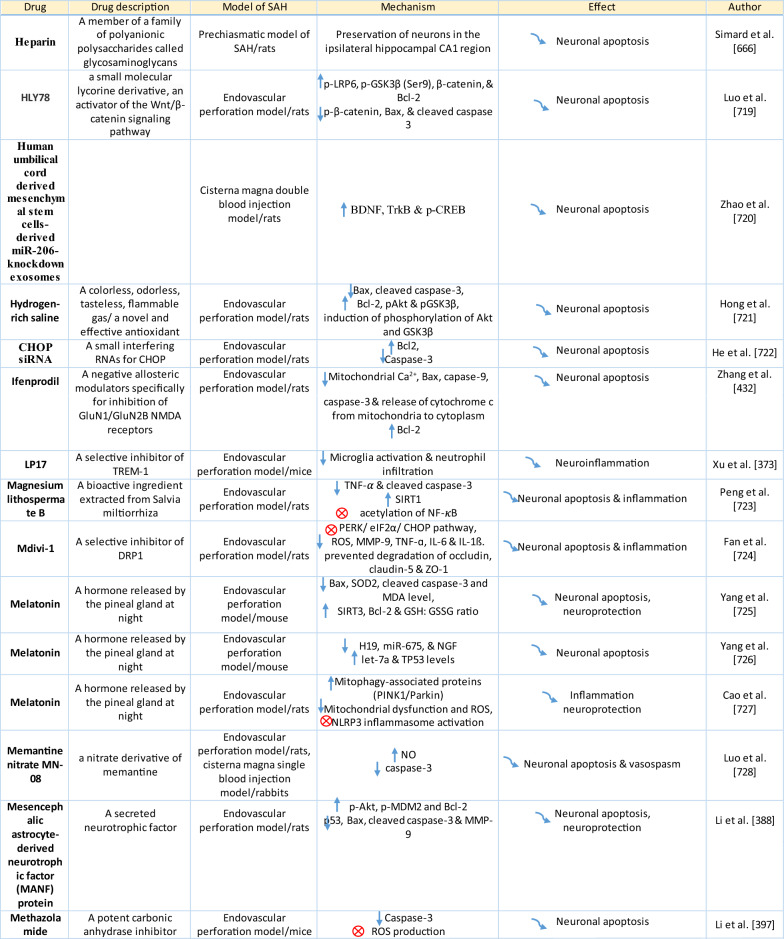

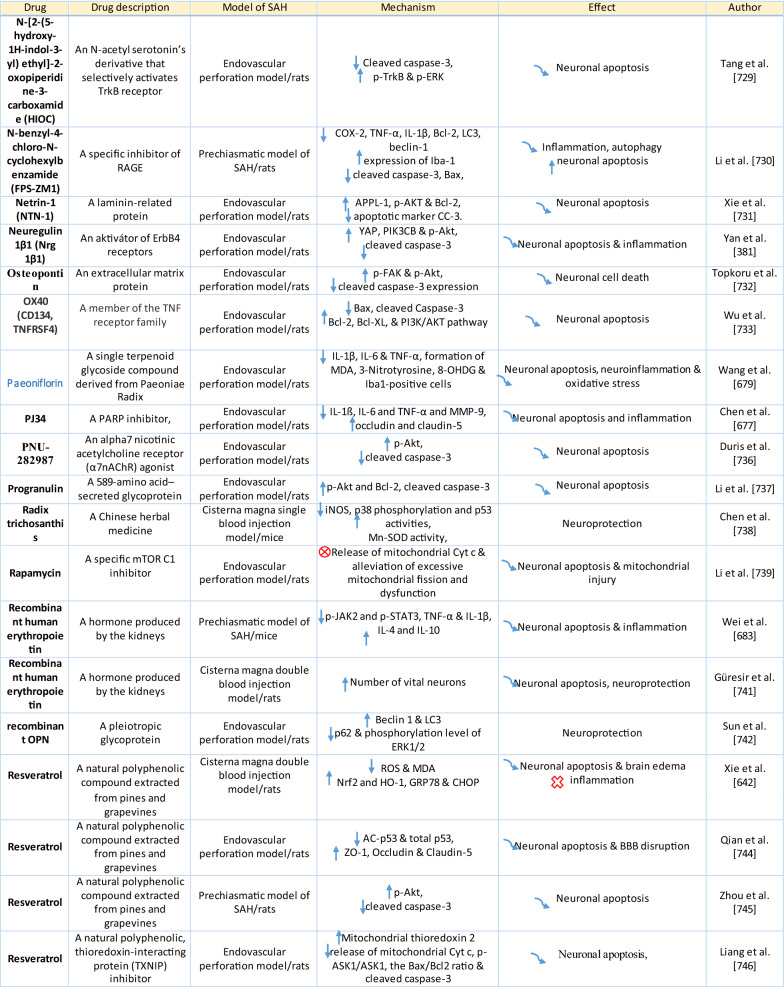

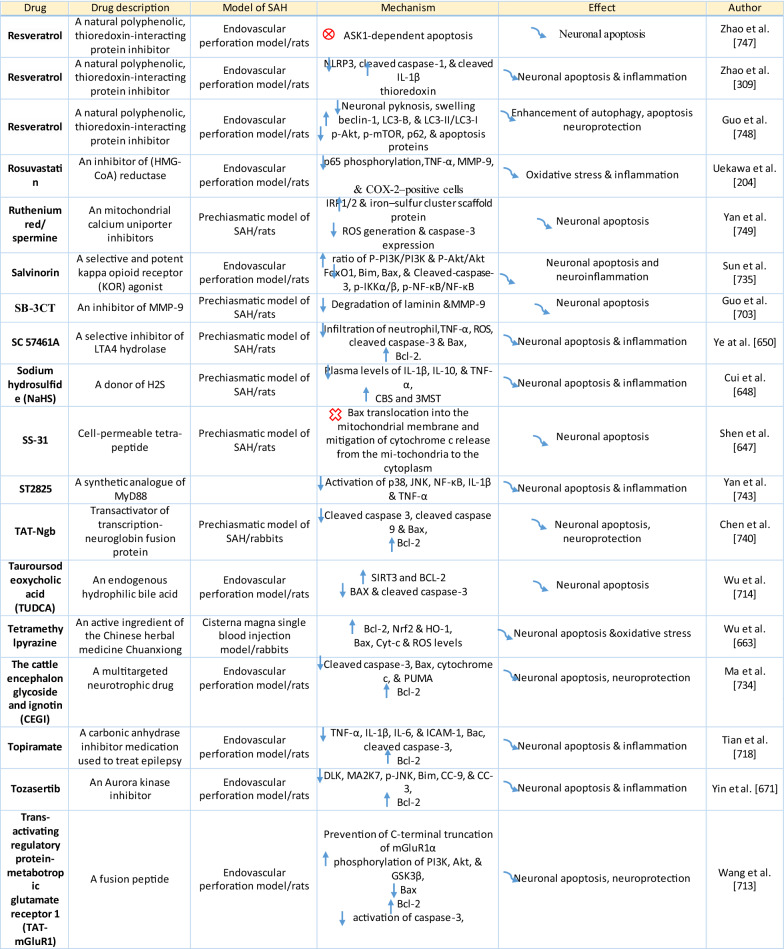

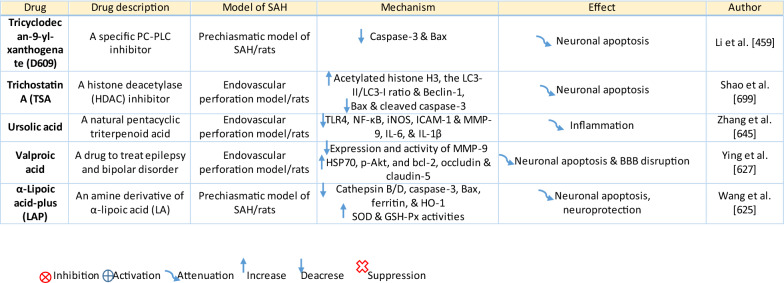
Potential drugs affecting neurons after SAH—from 2010 to 2021

The effects of some drugs that elicited good results in experimental studies have been evaluated in clinical trials. Although experimental data showed that administration of statins alleviates vasospasm and BBB disruption after SAH, randomized clinical trials did not demonstrate any benefit of simvastatin after SAH [610, 611].

The ALISAH (Albumin in Subarachnoid Hemorrhage) Pilot Clinical Trial evaluating the effect of albumin in patients after SAH showed a possible neuroprotective effect including a lower incidence of vasospasm, DCI, and cerebral infarction in a dose-dependent manner 90 days after SAH [612, 613]. Reducing the pro-inflammatory polarization of microglia can contribute to the beneficial effect of albumin after SAH.

Inhibiting IL-1α results in the attenuation of neuroinflammation after SAH, and this was achieved in an experimental SAH model using an IL-1 receptor antagonist. The SCIL-SAH (The subcutaneous Interleukin-1Ra in SAH) clinical trial using the IL-1 receptor antagonist showed suppression of the IL-1–mediated response and inflammation following SAH but did not demonstrate an effect on outcome [614].

Experimental evidence shows that heparin decreases the number of Iba1-positive microglia and reduces neuroinflammation after SAH. Therefore, promising results can be expected from the ongoing ASTROH (Aneurysmal Subarachnoid Hemorrhage Trial RandOmizing Heparin) trial aimed at evaluating the effect of continuous low-dose intravenous unfractionated heparin on the development of inflammation and outcome in patients after SAH [615].

Although hydroxyethyl starch stabilizes the BBB by increased ZO-1 and occludin expression, no beneficial effect has been demonstrated in clinical use. In line with this, a randomized clinical trial assessing the effect of euvolemia induced by hydroxyethyl starch did not show any effect on patient outcome after SAH.

The development of cerebrovascular inflammation is one of the main pathophysiological cascades after SAH. Many experimental studies have concentrated on alleviating inflammatory changes after bleeding. Despite the positive effect of various anti-inflammatory drugs, there are only a few clinical studies describing the effect of these drugs in clinical practice [619].

## Concluding remarks

As described in this review, every single component of the NVU has a significant role in SAH pathophysiology. One important observation from our review and other sources is that the strict classical division of main phases following SAH into EBI and DCI is obsolete. It is more likely that DCI is just a continuation of EBI. Nonetheless, the main pathophysiological event after SAH is the development of neuroinflammation in different components of the BBB and NVU.

We may conclude, that endothelial cells, by expressing tight junction proteins as well as regulating transporter systems, are responsible for the major barrier function. Hence, during SAH, alteration of BBB integrity and subsequent behavioral changes of endothelial cells could influence interactions in the NVU. Vascular smooth muscles are characterized by their contractile ability and their role in vasospasms. Vasospasms occur in the later phase of the SAH due to ion channel misregulation. SAH elicits a general inflammatory reaction in the CNS, predominantly affecting pericytes, microglia, and astrocytes. Blood and blood degradation products induce neuronal death by initiating apoptosis. Nevertheless, NVU components can modulate these outcomes through their protective mechanisms. Importantly, there seem to be some gender differences in how the NVU unit reacts to SAH, and this is driven by sex hormones. Nonetheless, their effects should be more carefully analyzed, mainly because of the wide use of contraceptives.

As we have seen, the pathophysiology of SAH is highly complex. Therefore, it is clear that treatment of the SAH should be similarly complex as well. We cannot expect one molecule to affect all components of the NVU in a positive direction. Future research should therefore focus on finding an ideal combination of drugs affecting the major pathophysiological aspects of SAH and should concentrate mainly on clinical practice by employing randomized clinical trials.

## Data Availability

Not applicable.

## Abbreviations

3MST: 3-Mercaptopyruvate Sulfur Transferase
4-HNE: 4-Hydroxynonenal
5-HT: 5-Hydroxytryptamine
8-OHDG: 8-Hydroxy-2’ -Deoxyguanosine
Aggf1: Angiogenic Factor with G Patch and FHA Domains 1
AIM: Absent in Melanoma
Akt: Akt Protein Kinase
ALDH: Mitochondrial aldehyde dehydrogenase
ApoE: Apolipoprotein E
AQP: Aquaporin
ARE: Antioxidant response element
ASC: Apoptosis-Associated Speck-Like Protein Containing a C-Terminal Caspase Recruitment Domain
ASK: Apoptosis signal-regulating kinase
AT1: Angiotensin II type 1
ATF: Activating Transcription Factor
Bax: Bcl-2-associated X protein
BBB: Blood–brain barrier
Bcl: B-cell lymphoma
BDNF: Brain-derived neurotrophic factor
Becn: Beclin
BK: Large-Conductance Ca^2^^+^-Activated K^+^ Channel
BKCa: Large-Conductance Ca-Activated K Channel
BLT: LTB4 receptor
BNIP: BCL2/adenovirus E1B 19 Kda interacting protein
C/EBPα: CCAAT-enhancer-binding protein α
C5a: Complement component 5a
CamK: Ca^2^^+^/Calmodulin-dependent Protein Kinase
CBS: Cystathionine b-synthase
CC-: Cleaved caspase
CCL: C–C Motif Chemokine Ligand
CCR: Chemokine receptor
CDKN1B: Cyclin-dependent kinase inhibitor 1B
CFTR: Cystic Fibrosis Transmembrane Conductance Regulator
CHOP: C/EBP homologous protein
CHP: Calcineurin-like EF hand protein
CNS: Central nervous system
COX: Cyclooxygenase
CREB: Camp response element-binding protein
CSD: Cortical spreading depolarization
CSF: Cerebrospinal fluid
CX3CL: CX3C-chemokine ligand
CX3CR: CX3C-chemokine receptor
CypA: Cyclophilin A
DAMP(s): Damage-Associated Molecular Pattern(s)
DCI: Delayed cerebral ischemia
DLK: Dual leucine zipper kinase
DRP: Dynamin-related gtpase
EAAC: Excitatory amino acid carrier
EAAT: Excitatory amino acid transporter
EBI: Early brain injury
ECs: Endothelial cells
EGFR: Epidermal growth factor receptor
eHACSs: Endfeet high-amplitude Ca^2^^+^ Signals
eIF2α: Eukaryotic initiation factor 2α
eNOS: Endothelial nitric oxide synthase
EphA4: Eph receptor A4
ErbB4: V-Erb-B2 avian erythroblastic leukemia viral oncogene homolog 4
ERK: Extracellular signal-regulated kinase
ET-1: Endothelin-1
ET_A_: Endothelin A
ET_A_R: Endothelin A receptor
ET_B_: Endothelin B
FAK: Focal Adhesion Kinase
FGFR: Fibroblast growth factor receptor
FKBP: FK506 binding protein
FLAP: 5-Lipoxygenase-Activating protein
FoxO3a: Forkhead Box Class O 3a
GAP: Growth-associated protein
GFAP: Glial fibrillary acidic protein
GFP: Green fluorescent protein
GLAST: Glutamate/aspartate transporter
GLT: Glutamate transporter
GluR: Glutamate receptor
GPR30: G protein-coupled receptor 30
GRP: Glucose-regulated protein
GSDMD: Gasdermin D
GSH: Glutathione
GSK3β: Glycogen Synthase Kinase 3ß
GSN: Gelsolin
GSSG: Oxidized Glutathione
H3K27: Histone H3 Lysine 27
Hb: Hemoglobin
HCN: Hyperpolarization-activated/cyclic nucleotide-gated
HDAC: Histone Deacetylases
HIF-1α: Hypoxia-inducible factor-1 α
HMGB1: High-Mobility Group Box 1 Protein
HMG-CoA: 3-Hydroxy-3-Methylglutaryl-Coenzyme A
HO-1: Heme-Oxygenase 1
HSP70: Heat Shock Protein 70
ICAM-1: Intercellular adhesion molecule 1
ICP: Intracranial pressure
IL: Interleukin
iNOS: Inducible nitric oxide synthase
IP_3_: Inositol trisphosphate
IRAK: Interleukin-1 Receptor-Associated Kinase
IRF: Interferon regulatory factor
JAK: Janus Kinase
JAM: Junctional adhesion molecule
JIP: JNK-Interacting Protein
JMJD: Jumonji Domain-Containing
JNK: Jun N-terminal kinase
Kir: Inward-rectifying K^+^ channel
LRP: Lipoprotein receptor-related protein
LXA: Lipoxin A
MA2K7: Member of IP3/MA2K7/JNK Pathway
MANF: Mesencephalic astrocyte-derived neurotrophic factor
MAPK: Mitogen-activated protein kinase
MCP: Monocyte chemoattractant protein
MDA: Malondialdehyde
MEF2D: Myocyte Enhancer Factor 2D
Mfsd2a: Major Facilitator Superfamily Domain-Containing Protein 2a
mGluR: Metabotropic glutamate receptor
MLCK: Myosin light chain kinase
MMP: Matrix metalloproteinase
MPO: Myeloperoxidase
MSC: Mesenchymal stem cells
MSK: Mitogen- and stress-activated protein kinase
mTOR: Mammalian target of rapamycin
MyD88: Myeloid differentiation primary response protein 88
NEK: Serine/threonine protein kinase
Ngb: Neuroglobin
NGF: Nerve growth factor
NHE: Na+H+-exchanger
NLK: Nemo-like kinase
NLRP: Leucine-Rich Rep0eat (LRR)-Containing Protein
nNOS: Neuronal nitric oxide synthase
NOX: NADPH oxidases
NQO1: NAD(P)H:Quinone Oxidoreductase
NR2A: N-Methyl-D-aspartate receptor subunit 2A
NR2B: N-Methyl-D-aspartate receptor subunit 2B
NR3B: N-Methyl-D-aspartate receptor subunit 3B
Nrf2: Nuclear factor-erythroid 2-related factor 2
NVU: Neurovascular unit
OPN: Osteopontin
OxyHb: Oxyhemoglobin
P2X7R: P2X7 receptor
p75NTR: Pan75 neurotrophin receptor
PAR: Proteinase-activated receptor
PARP: Poly (ADP)-ribose polymerase
PCNA: Proliferating cell nuclear antigen
PC-PLC: Phosphatidylcholine-specific phospholipase C
PDE-V: Phosphodiesterase Type V
PDGFR-β: Platelet-derived growth factor receptor Β
Peli1: Pellino homolog 1
PERK: Pancreatic Endoplasmic Reticulum Kinase
P-gP: P-glycoprotein
PI3k: Phosphoinositide 3-kinase
PK2: Prokineticin 2
PKA: Protein kinase A
PKC: Protein kinase C
PPAR: Peroxisome proliferator-activated receptor
Prx2: Peroxiredoxin 2
PSD-95: Postsynaptic density protein-95
PSGL: P-selectin glycoprotein ligand
PTEN: Phosphatase and Tensin Homolog Deleted on Chromosome 10
PTH-R1: Parathyroid hormone receptor-1
PUMA: P53 upregulated modulator of apoptosis
RAGE: Advanced glycation end products
REDD1: Regulated in development and DNA damage responses 1
RhoA: Ras homolog family member A
ROCK: Rho-associated protein kinase
ROMO: Reactive oxygen species modulator
ROS: Reactive oxygen species
RYR: Ryanodine receptor type
S1P1: Sphingosine-1-phosphate receptor-1
SAH: Subarachnoid Hemorrhage
SDF1α: Stroma-Cell-Derived Factor 1α
SENP: Small ubiquitin-like modifier-specific protease
SIRT: Sirtuin
SMemb: Embryonic smooth muscle myosin heavy chain
SMIT: Na+/Myo-inositol transporter
SM-MHC: Smooth muscle myosin heavy chain
SOD: Superoxide Dismutase
SOX: SRY-box transcription factor
SynCAM: Synaptic cell adhesion molecule
TAK: Tgfβ-activated kinase
TGR: Trans-membrane G protein-coupled receptor
TIMP: Tissue Inhibitors of Metalloproteinases
TIRAP: Toll/Interleukin-1 Receptor Domain-Containing Adapter Protein
TJ(s): Tight Junction(s)
TLR: Toll-like receptor
TM: Thrombomodulin
TNC: Tenascin-C
TNF-R1: TNF receptor 1
TNF-α: Tumor necrosis factor alpha
TP53: Tumor Protein P53
TREM: Triggering receptor expressed on myeloid cells
Trk: Tropomyosin receptor kinase
TRPC: Transient receptor potential channel
TSPO: Translocator protein
TXA2: Thromboxane A2
TXNIP: Thioredoxin-interacting protein
VASP: Vasodilator-stimulated phosphoprotein
VCAM-1: Vascular cell adhesion protein 1
VDAC: Voltage-dependent anion channels
VDCCs: Voltage-dependent Ca2+ channels
VE-cadherin: Vascular endothelial cadherin
VEGF: Vascular endothelial growth factor-A
VSCC: Voltage-Sensitive Ca2+ Channel
VSMC: Vascular smooth muscle cells
vWF: Von Willebrand factor
YAP: Yes-associated protein
ZO: Zonula occludens
α-SMA: Alpha-smooth muscle actin
